# Exploring the dominant endophytic pleosporalean fungi in *Poaceae* plants: taxonomic novelties within the suborder *Massarineae*

**DOI:** 10.1080/21501203.2025.2569933

**Published:** 2025-12-06

**Authors:** Keyu Chen, Lijuan Mao, Kelin Li, Chulong Zhang

**Affiliations:** a Ministry of Agriculture and Rural Affairs Key Laboratory of Molecular Biology of Crop Pathogens and Insect Pests, Zhejiang Key Laboratory of Biology and Ecological Regulation of Crop Pathogens and Insects, Institute of Biotechnology, Zhejiang University, Hangzhou, China; b Analysis Center of Agrobiology and Environmental Science, Zhejiang University, Hangzhou, China

**Keywords:** Endophytic fungi, genealogical concordance phylogenetic species recognition, *Massarineae*, new taxa, *Pleosporales*, taxonomy

## Abstract

The suborder *Massarineae*, within the order *Pleosporales* (class *Dothideomycetes*), includes many fungi that act as dominant endophytes in *Poaceae* plants. However, despite recent advancements, the phylogeny and taxonomy of endophytic *Massarineae* remain insufficiently resolved. In this study, comprehensive phylogenetic and morphological analyses were conducted for two key families within *Massarineae* – *Didymosphaeriaceae* and *Periconiaceae* – by incorporating collections of novel endophytic strains from *Poaceae* hosts. Multilocus phylogenetic analyses revealed distinct clades in both families, leading to the proposal of 10 new taxa within *Massarineae*. Within *Didymosphaeriaceae*, two new genera (*Proxiconiothyrium* gen. nov. and *Chlamydosphaeromyces* gen. nov.) and three new species (*Proxiconiothyrium yunnanense* sp. nov., *Neptunomyces yunnanensis* sp. nov., and *Chlamydosphaeromyces glomeratus* sp. nov.) are described; for *Periconiaceae*, three new species (*Periconia catenata* sp. nov., *P*. *neomacrospinosa* sp. nov., *P*. *longibrachiatum* sp. nov.), one new combination (*P*. *fueloephazae* comb. nov.), and one new sensu stricto taxon (*P*. *macrospinosa* s.s.) are introduced. Detailed morphological descriptions are provided for each newly established taxon. The results significantly improve the taxonomic and phylogenetic framework for *Massarineae* endophytes and provide a valuable reference for exploring the diversity and evolutionary differentiation within other taxonomic groups of *Pleosporales*.

## 1. Introduction

*Dothideomycetes*, belonging to *Ascomycota*, represent the largest and most ecologically diverse class of fungi (Hyde et al. 2013; Liu et al. 2017; Hongsanan et al. 2020). This class has attracted considerable attention from mycologists due to its significance in fungal diversity and classification. Among *Dothideomycetes*, *Pleosporales* is the largest order, comprising approximately one-fourth of the class and is being further divided into 94 families based on morphological and phylogenetic evidence (Hyde et al. 2013, 2024; Liu et al. 2017; Hongsanan et al. 2020). Species within *Pleosporales* exhibit highly diverse lifestyles, including saprotrophs, epiphytes, endophytes, pathogens, mycoparasites of fungi and insects, and lichenised fungi (Hongsanan et al. 2020). While researchers have intensively investigated saprophytic and pathogenic pleosporalean fungi – focusing on their classification, biological characteristics (Zhang et al. 2012; Crous et al. 2018; Wijayawardene et al. 2022; Yang et al. 2022), and pathogenic mechanisms (Leukel and Johnson 1948; Franco et al. 2017; Woudenberg et al. 2017; Seblani et al. 2023) – studies addressing endophytic fungi lag behind. Systematic isolation and identification of pleosporalean endophytes are essential to reveal their diversity, ecological functions, and interactions with host plants.

*Massarineae* is a major suborder within *Pleosporales* (Hyde et al. 2013). Tanaka et al. (2015) revised the taxonomy of *Massarineae* through analyses of small subunit rDNA (SSU), large subunit rDNA (LSU), and the translation elongation factor 1-alpha gene (*TEF1*). By integrating molecular data with morphological characteristics, their study recognises 12 families in this suborder, including *Didymosphaeriaceae* and *Periconiaceae*.

*Didymosphaeriaceae* was introduced by Munk (1953), with *Didymosphaeria* as the type genus. Members of this family include saprobic, endophytic, or pathogenic fungi, occurring on woody branches, herbaceous stems, leaves, and occasionally as human pathogens (Hyde et al. 2013; Hongsanan et al. 2020; Wanasinghe et al. 2024). This family is morphologically characterised by globose to subglobose, immersed or semi-immersed ascomata, producing one-septate ascospores and trabeculate pseudoparaphyses that predominantly anastomose above the asci (Aptroot 1995; Hyde et al. 2013; Ariyawansa et al. 2014a). Some genera, such as *Alloconiothyrium* (Verkley et al. 2014), *Neptunomyces* (Gonçalves et al. 2019), *Dictyoarthrinium* (Samarakoon et al. 2020), and *Pleoardoris* (Pinchi-Davila et al. 2023), are only known from their asexual morphs, which produce *fusicladium*-like, *phoma*-like, or irregular conidia (Verkley et al. 2014; Hongsanan et al. 2020; Du et al. 2021).

Despite advances in morphological studies, molecular research on *Didymosphaeriaceae* remains limited, resulting in ongoing taxonomic uncertainty within the family. For example, Zhang et al. (2012) recognised only three genera (*Appendispora*, *Didymosphaeria*, and *Phaeodothis*). Subsequently, Ariyawansa et al. (2014a) reassessed *Didymosphaeriaceae* using nrDNA sequences to evaluate its phylogenetic coherence. Wanasinghe et al. (2016) integrated all available *Didymosphaeriaceae* genera from GenBank to construct a more comprehensive phylogenetic framework for this family. Currently, 39 genera are accepted within *Didymosphaeriaceae* (Hyde et al. 2024; Pem et al. 2024). Nevertheless, a robust classification system still requires extensive sampling, particularly of underrepresented genera and species.

*Periconiaceae* was introduced by Nannizzi (1934), with *Periconia* designated as the type genus. Recognised as a sister taxon to *Massarinaceae* within *Massarineae*, *Periconiaceae* was originally composed of four genera: *Noosia*, *Bambusistroma*, *Flavomyces*, and *Periconia* (Crous et al. 2011; Adamčík et al. 2015; Knapp et al. 2015; Tanaka et al. 2015). Species of *Periconia* are cosmopolitan and frequently isolated from herbaceous plants (Ellis 1968; Crous et al. 2018; Fors et al. 2020; Tian et al. 2022; Wijayawardene et al. 2022; Su et al. 2023). While some species, such as *P. circinata* (causing milo disease) (Leukel and Johnson 1948) and *P. igniaria* (causing leaf spot) (Kolomiets et al. 2008), act as plant pathogens, most species are saprophytic fungi (Liu et al. 2017; Crous et al. 2018; Jayasiri et al. 2019) or endophytic fungi (Yuan et al. 2009; Knapp et al. 2019). Notably, endophytic fungi of *Periconia* commonly colonise *Poaceae* roots (Knapp et al. 2019; Liu et al. 2021) and have demonstrated significant potential in enhancing plant resistance and biological control (Knapp et al. 2015; Yuan et al. 2020; Høyer et al. 2022; Gaber et al. 2023).

Morphologically, *Periconia* is primarily known from its asexual morphs (Hongsanan et al. 2020). However, a few species, such as *P. homothallica* and *P. pseudodigitata*, can produce sexual morphs (Tanaka et al. 2015; Hongsanan et al. 2020). The sexual morphs are characterised by scattered, globose to obpyriform ascomata with a central ostiole and neck. The ascomatal wall consists of multiple cell layers, and each ascus contains eight well-arranged, hyaline, broadly fusiform ascospores with a complete sheath (Booth 1968; Tanaka et al. 2015; Yang et al. 2022). In the asexual morphs, *Periconia* is hyphomycetous, producing pigmented conidiophores that are septate, upright or slightly curved with smooth to echinulate, thick-walled surfaces, or sometimes lacking conidiophores entirely. Conidiogenous cells, which may be monoblastic to polyblastic, are borne on the stipe and branches, occasionally bearing small, pimple-like buds. The conidia are arranged in chains or singly, golden brown to dark brown, spherical or ellipsoidal, aseptate, with smooth or verruculose surfaces, and some species possess spines (Lefebvre et al. 1949; Ellis 1968; Hongsanan et al. 2020; Tian et al. 2022; Yang et al. 2022; Shen et al. 2024).

Recent phylogenetic studies indicate that *Periconia* is polyphyletic within *Periconiaceae*, as the three monotypic genera *Bambusistroma*, *Flavomyces*, and *Noosia* consistently clustered with other *Periconia* species (Knapp et al. 2015, 2019; Tanaka et al. 2015; Crous et al. 2018; Hyde et al. 2018; Phookamsak et al. 2019; Tian et al. 2022). Moreover, morphological and phylogenetic evidence has led to the synonymisation of *B. didymosporum* and *N. banksiae* with *P. didymosporum* and *P. banksiae*, respectively (Yang et al. 2022). Nonetheless, the taxonomic status of *F. fueloephazae*, characterised only by its mycelial morphology, still requires further investigation and revision (Tanaka et al. 2015; Yang et al. 2022).

Endophytic fungi are defined as “asymptomatic microbial partners that are intimately associated with and co-inhabit healthy internal plant tissues with the ability to confer benefits, co-evolve, and alter their lifestyle depending on plant life stages and adverse conditions” (Liao et al. 2025). Endophytism represents one of the primary ecological strategies adopted by fungi. These fungi exhibit substantial diversity and play crucial roles in ecosystems. In previous studies, endophytic fungi were collected and isolated from healthy *Poaceae* plants in China (Feng et al. 2021; Liu et al. 2021). Classification and characterisation of dominant endophytes, such as those in the order *Magnaporthales* (Feng et al. 2024) and the genus *Trichoderma* (Zhao et al. 2025), have uncovered numerous novel taxa associated with *Poaceae* plants. These findings highlight the rich and largely unexplored diversity of endophytes in *Poaceae*.

Traditional phylogenetic approaches relying on a limited number of gene regions often fail to provide sufficient resolution at the species level, hindering accurate taxonomic conclusions (Maharachchikumbura et al. 2021a). To overcome these limitations, the present study adopts an integrative approach to revise the taxonomy of dominant endophytic pleosporalean fungi associated with *Poaceae* plants. The objectives are to: (1) reconstruct multilocus phylogenies of *Didymosphaeriaceae* and *Periconiaceae* to inform taxonomic revisions according to genealogical concordance phylogenetic species recognition (GCPSR); (2) assess species delimitation within *Periconia macrospinosa* sensu lato based on phylogenetic analyses, using newly collected samples along with split network and pairwise homoplasy index (PHI) tests (Bruen et al. 2006); and (3) describe potential novel endophytic taxa within *Didymosphaeriaceae* and *Periconiaceae*, supported by both phylogenetic and morphological evidence.

## 2. Materials and methods

### 2.1. Sample collection and fungal isolation

Fungal endophytes were isolated following protocols described in previous studies (Feng et al. 2021; Liu et al. 2021). From 2015 to 2019, healthy *Poaceae* plant samples were collected from three nature reserves representing distinct climatic zones in China: Xishuangbanna National Nature Reserve (E 99°55′–101°50′, N 21°10′–22°40′) in Yunnan Province; Baishanzu National Nature Reserve (E 119°06′–119°15′, N 27°46′–27°58′) in Zhejiang Province; and Xilingol Grassland National Nature Reserve (E 110°50′–119°58′, N 41°30′–46°45′) in Inner Mongolia Autonomous Region. For endophytic fungal isolation, the healthy-looking samples were washed with tap water, and then sequentially surface-disinfected in 75% ethanol for 3 min, 1% sodium hypochlorite for 10 min, and rinsed with sterile water five times. The surface-disinfected tissues were cut into small sections (about 5 mm) and were placed on malt extract agar (MEA) plates supplemented with 50 mg/L ampicillin and streptomycin sulphate. The plates were then incubated at 25 °C in darkness, and the single-hyphal tips were transferred onto fresh MEA plates for purification and preservation. In total, 1,821 endophytic fungal strains were isolated from various *Poaceae* plants, of which 486 strains belonged to the *Pleosporales*.

The isolated strains were deposited in the Ministry of Agriculture and Rural Affairs Key Laboratory of Molecular Biology of Crop Pathogens and Insect Pests, Institute of Biotechnology, Zhejiang University, China. Additionally, holotype and ex-type living cultures were deposited in the China Guangdong Microbial Culture Collection Centre (GDMCC).

### 2.2. DNA extraction, PCR amplification, and sequencing

Fungal genomic DNA extraction, PCR amplification, and sequencing were performed as described in Feng et al. (2024). Partial regions of several genomic loci were amplified using polymerase chain reaction (PCR), including the large subunit rRNA (LSU), internal transcribed spacer (ITS), actin (*ACT*), RNA polymerase II second largest subunit (*RPB2*), translation elongation factor 1-alpha (*TEF1*), and β-tubulin (*TUB2*). PCR amplification was carried out in a 20 μL reaction mixture consisting of 10 μL Easy Flash PCR MasterMix (Easy-Do, China), 0.8 μL DNA template, and 0.8 μL of each forward and reverse primer. ITS and LSU were amplified with primers ITS1-F and LR3 (Vilgalys and Hester 1990; Gardes and Bruns 1993). *ACT* was amplified with ACT-512F and ACT-2Rd (Carbone and Kohn 1999; Groenewald et al. 2006). *RPB2* was amplified with fRPB2-7cR and fRPB2-5F (Liu et al. 1999). *TEF1* was amplified with EF1-983F and EF1-2212 R or EF1-2218 R (Rehner and Buckley 2005). *TUB2* was amplified with Bena-T1 and Bena-T22 (O’Donnell and Cigelnik 1997). The PCR protocols were followed as suggested by previous studies (Zhang et al. 2011; Fors et al. 2020; Yan and Zhang 2024). Annealing temperatures were set to 56 °C for the ITS-LSU partition, 61 °C for *ACT*, 53–58 °C for *RPB2*, 58 °C for *TEF1*, and 52–56 °C for *TUB2*. PCR products were sent to Sunya Biotechnology (Hangzhou, China) for purification and sequencing. Sequences of *Didymosphaeriaceae* and *Periconiaceae* generated in this study were deposited in the National Microbiology Data Centre (NMDC) and the GenBank nucleotide database, respectively.

### 2.3. Phylogenetic analysis

Phylogenetic analysis was carried out as described in Feng et al. (2024). Sequences of the ITS and LSU loci for all strains from this study were generated for initial identification, and representative strains were selected for further phylogenetic analysis. The sequence accession numbers for *Didymosphaeriaceae* and *Periconiaceae* are provided in Tables S1 and S2, respectively. Basic sequence alignment was conducted using BLAST (https://blast.ncbi.nlm.nih.gov), followed by multiple sequence alignment using MAFFT v. 7 (Katoh and Standley 2013), and trimming with trimAl v. 1.2rev57 (Capella-Gutiérrez et al. 2009). The trimmed sequences were concatenated with SequenceMatrix v. 1.7.8 (Vaidya et al. 2011), and the optimal nucleotide substitution model was determined using ModelFinder v. 2.2.0 (Subha et al. 2017). Phylogenetic congruence was assessed by comparing topologies among single-locus trees, and multilocus phylogenetic trees were then constructed using IQ-TREE v. 2.2.0 (Lam-Tung et al. 2015) with the maximum likelihood (ML) method. Additionally, MrBayes v. 3.2.6 (Ronquist et al. 2012) was used for Bayesian inference (BI) tree building. The phylogenetic analyses were conducted on PhyloSuite v. 1.2.3 (Zhang et al. 2020). The maximum likelihood bootstrap proportions (MLBP) ≥ 70% and Bayesian posterior probabilities (BIPP) ≥ 0.90 are presented at the nodes, with all values rounded to two decimal places.

To investigate species delimitation, the level of recombination among different strains in *Periconia macrospinosa* was determined using single-locus and concatenated sequence alignments. Split networks were constructed in SplitsTree (Huson and Bryant 2006) applying LogDet and NeighborNet methods, while the pairwise homoplasy index (Φw) was calculated to assess recombination levels among closely related species.

### 2.4. Morphological characterization

Representative strains identified as new taxa of *Didymosphaeriaceae* and *Periconiaceae* were collected for morphological observation. Purified strains were cultivated on potato dextrose agar (PDA) plates at 25 °C in 12/12 h light/dark cycles, and daily measurements of colony diameters were taken. To induce sporulation, a method described by Knapp et al. (2015) was followed, with strains being individually inoculated on MEA, modified Melin-Norkrans (MMN) media, synthetic nutrient-poor agar (SNA) supplemented with *Poaceae* leaves, oatmeal agar (OA), and potato sucrose agar (PSA) supplemented with wheat straw, and were then sealed with breathable tape. Mycelial wounding, cyclic UV-A irradiation (12 h/day at 365 nm), and a temperature gradient (18–28 °C) were also employed to induce sporulation. The colony characteristics and sporulation conditions were observed and documented after 2 weeks. At least 30 conidia per strain were measured, and the observations were conducted using a SOPTOP DMSZ8 stereomicroscope (Ningbo Sunny Instruments Co., Ltd.) and a Nikon Eclipse 80i microscope (Nikon Corp.).

## 3. Results

### 3.1. *Phylogenetic analysis of* Didymosphaeriaceae *reveals considerable taxonomic novelty*

The study employed the GCPSR concept (Taylor et al. 2000), which requires at least three noncontradictory, unlinked loci for reliable inference. Initially, individual locus trees were constructed for three loci: rDNA (SSU + LSU + ITS), *RPB2*, and *TEF1*, at the *Didymosphaeriaceae* family level, with the two *Massarinaceae* taxa (*Massarina eburnea* CBS 473.64 and *Byssothecium circinans* CBS 675.92) used as outgroups (Figures S1–S3). The phylogenetic trees inferred from rDNA, *RPB2*, and *TEF1* exhibited similar topologies for *Didymosphaeriaceae* and recovered the family as a monophyletic group with strong support (72% MLBP/1.00 BIPP for rDNA; 89% MLBP/1.00 BIPP for *RPB2*; and 91% MLBP/1.00 BIPP for *TEF1*). The family was subdivided into 35 clades (labelled D1–D35), with most clades corresponding to genera and receiving robust support, although some showed low support (D6 and D7 in the rDNA tree; D8 in the *TEF1* tree).

**Figure 1.**
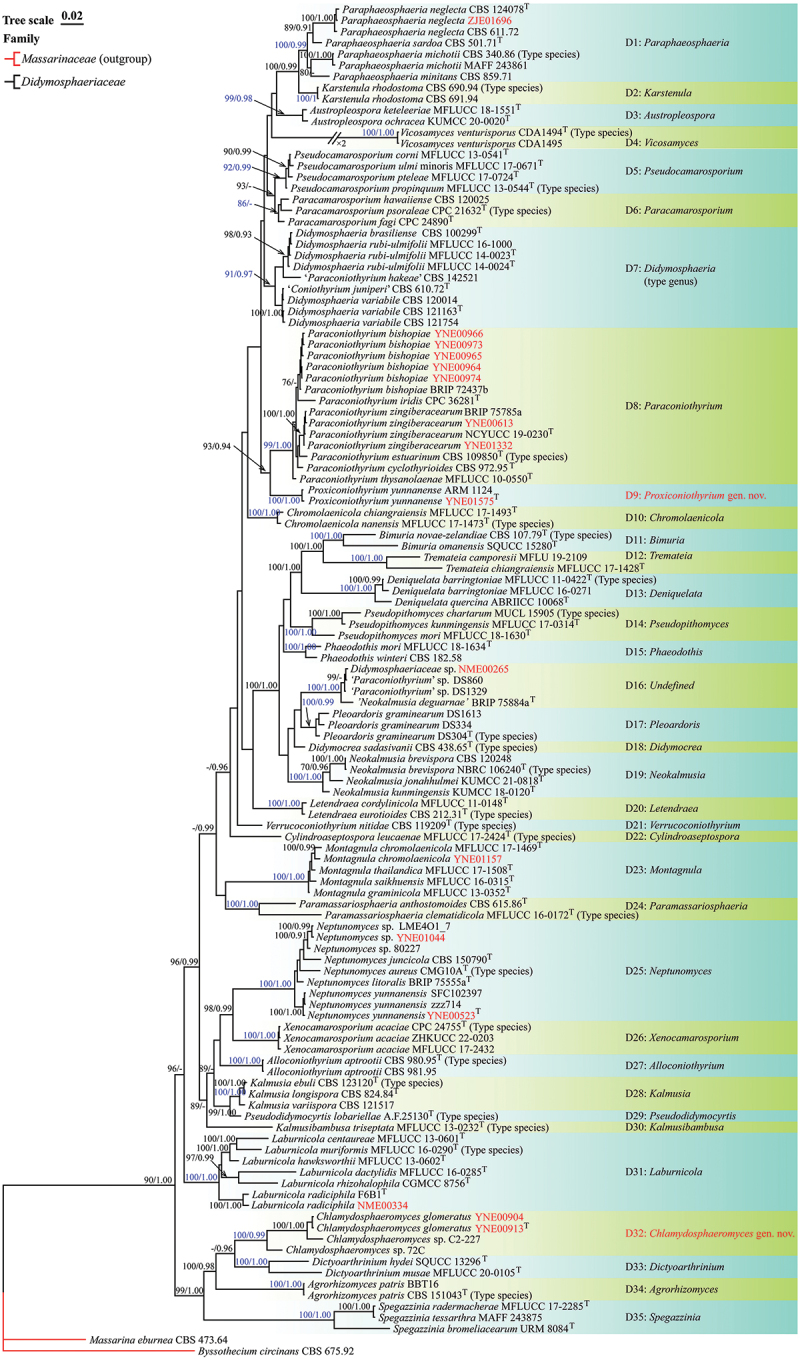
Phylogenetic tree based on the combined dataset (SSU, LSU, ITS, *RPB2*, and *TEF1*) of *Didymosphaeriaceae*. The tree is rooted to *Massarina eburnea* CBS 473.64 and *Byssothecium circinans* CBS 675.92. MLBP ≥ 70% and BIPP ≥ 0.90 are presented above the branch leading to that node (MLBP/BIPP). Strains in this study are indicated in red and the support values for each distinct major clade are displayed in blue. Holotype and ex-type strains are denoted by superscript T, and type species of the corresponding genus are marked in parentheses.

To improve clade-level resolution within the family, a concatenated phylogeny was inferred from the three loci (rDNA, *RPB2*, and *TEF1*). The resulting multilocus tree (Figure 1) was well resolved and recovered *Didymosphaeriaceae* as a strongly supported monophyletic group (90% MLBP/1.00 BIPP). Thirty-five clades were recognised, most corresponding to genera with robust support, although clade D6 (Figure 1) showed low support. Among these 35 clades, 32 correspond to previously described genera in *Didymosphaeriaceae*, including ‘*Agrorhizomyces*’, *Alloconiothyrium*, *Austropleospora*, *Bimuria*, *Chromolaenicola*, *Cylindroaseptospora*, *Deniquelata*, *Dictyoarthrinium*, *Didymocrea*, *Didymosphaeria* (type genus), *Kalmusia*, *Kalmusibambusa*, *Karstenula*, *Laburnicola*, *Letendraea*, *Lineostroma*, *Montagnula*, *Neokalmusia*, *Neptunomyces*, *Paracamarographium*, *Paracamarosporium*, *Paraconiothyrium*, *Paramassariosphaeria*, *Paraphaeosphaeria*, *Phaeodothis*, *Pleoardoris*, *Pseudocamarosporium*, *Pseudodidymocyrtis*, *Pseudopithomyces*, *Pseudotrichia*, *Spegazzinia*, *Tremateia*, *Verrucoconiothyrium*, *Vicosamyces*, and *Xenocamarosporium*. Additionally, strains YNE01044 and YNE00523 clustered with known *Neptunomyces* species within clade D25 (Figure 1) with robust support; YNE01044 clustered with an unidentified *Neptunomyces* strain (LME4O1_7), and YNE00523 grouped with two unidentified *Neptunomyces* strains (SFC102397 and zzz714). These two distinct subclades potentially represent two new species of *Neptunomyces*.

The remaining three clades represent undescribed genera within *Didymosphaeriaceae*. First, strain YNE01575, along with a closely related strain ARM1124, formed a distinct clade (D9, Figure 1) with robust support, representing a new genus and species. Second, the type species of *Paraconiothyrium* clustered with other *Paraconiothyrium* species in clade D8 (Figure 1), and the type species of *Neokalmusia* clustered with other *Neokalmusia* species in clade D19 (Figure 1). Meanwhile, strain NME00265 clustered with three misidentified strains (‘*Paraconiothyrium*’ sp. DS860, ‘*Paraconiothyrium*’ sp. DS1329, and ‘*Neokalmusia deguarnae*’ BRIP 75884a), forming a distinct clade (D16, Figure 1) with robust support; this clade potentially represents an undefined genus. Third, *Pseudopithomyces chartarum* (syn. *Leptosphaerulina chartarum*), the type species of *Pseudopithomyces*, clustered with *Pseudopithomyces kunmingensis* and *Pseudopithomyces mori*, forming a distinct clade (D14, Figure 1). Meanwhile, strains YNE00904 and YNE00913 clustered with two misidentified strains (‘*Leptosphaerulina chartarum* C2-227’ and ‘*Leptosphaerulina* cf. *chartarum* 72C’), forming a distinct clade (D32, Figure 1) with robust support; this clade potentially represents a new genus, with YNE00904, YNE00913, and C2-227 constituting a new species.

Based on combined molecular phylogeny and morphological characters, two new genera and three new species within *Didymosphaeriaceae* are proposed and are detailed in the taxonomy section.

### 3.2. *Phylogenetic analysis and species delimitation of* Periconiaceae *propose classification updates*

Initially, individual locus trees were constructed for five loci: rDNA (SSU + LSU + ITS), *ACT*, *RPB2*, *TEF1*, and *TUB2* at the *Periconiaceae* family level, with two *Massarinaceae* taxa (*Byssothecium circinan*s CBS 675.92 and *Helminthosporium solani* CBS 640.85) used as outgroups (Figures S4–S8). In the rDNA tree (Figure S4), *Periconiaceae* was recovered as a strongly supported monophyletic group (98% MLBP/1.00 BIPP). However, *Periconia macrospinosa* sensu lato was not resolved, and several *Periconia* species showed weak support with indistinct boundaries. In the *ACT* and *TUB2* trees (Figures S5, S8), species-level clades were generally well supported, whereas support for *Periconiaceae* monophyly remained low (< 70% MLBP/1.00 BIPP). In the *RPB2* tree (Figure S6), species-level support was consistently strong except for *P. chengduensis*, while *Periconiaceae* monophyly received only moderate support (75% MLBP/1.00 BIPP). In the *TEF1* tree (Figure S7), support for *Periconiaceae* monophyly was again low (< 70% MLBP/1.00 BIPP), several species (e.g. *P. cookei*, *P. delonicis*, *P. kumingensis*, *P. palmicola*, and *P. verrucosa*) were not distinguished, and support for several *Periconia* species was weak.

Subsequently, a concatenated five-locus dataset (rDNA, *ACT*, *RPB2*, *TEF1*, and *TUB2*) was analysed. The resulting tree (Figure 2) recovered *Periconiaceae* as a strongly supported monophyletic clade (100% MLBP/1.00 BIPP). Most described and putative novel *Periconia* species were resolved as distinct, well-supported clades, whereas a few taxa (e.g., *P. ananasi*, *P. byssoides*, *P. celtidis*, *P. citlaltepetlensis*, *P. dicranopteridis*, and *P. yantingensis*) showed relatively weak support or unclear boundaries.

**Figure 2.**
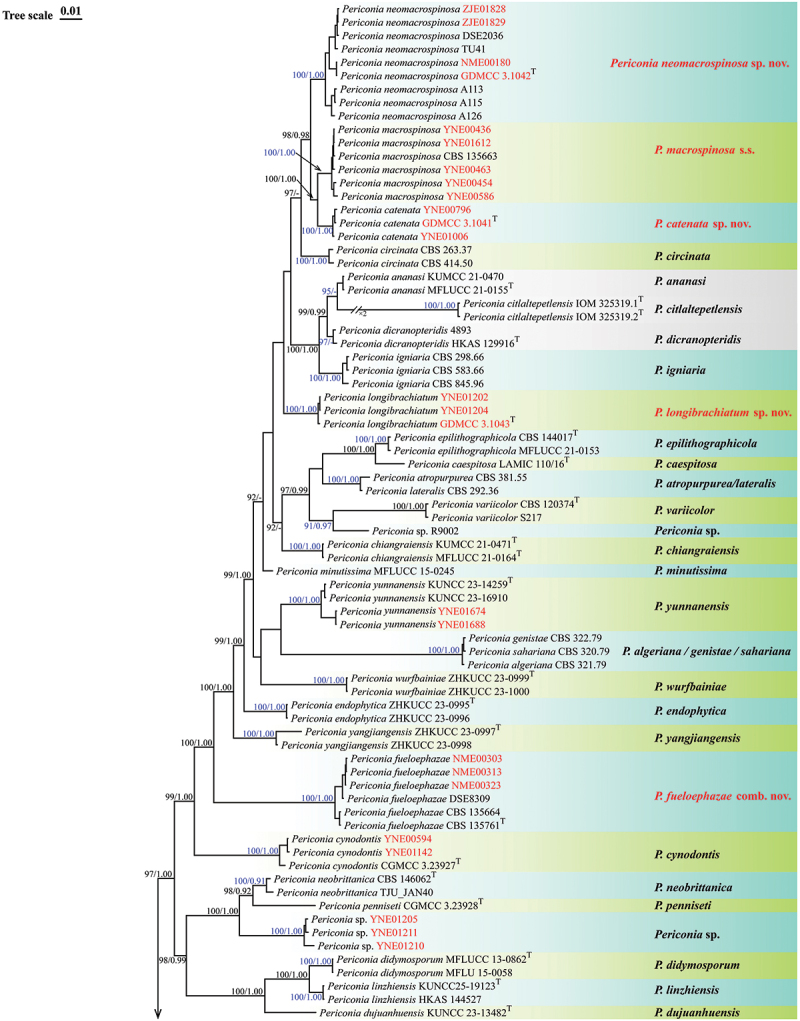
Phylogenetic tree based on the combined dataset (SSU, LSU, ITS, *ACT*, *RPB2*, *TEF1*, and *TUB2*) of *Periconia*. The tree is rooted to *Byssothecium circinans* CBS 675.92 and *Helminthosporium solani* CBS 640.85. MLBP ≥ 70% and BIPP ≥ 0.90 are presented above the branch leading to that node (MLBP/BIPP). Strains in this study are indicated in red and the support values for each distinct major clade are displayed in blue. Holotype and ex-type strains are denoted by superscript T.

**Figure 2.**
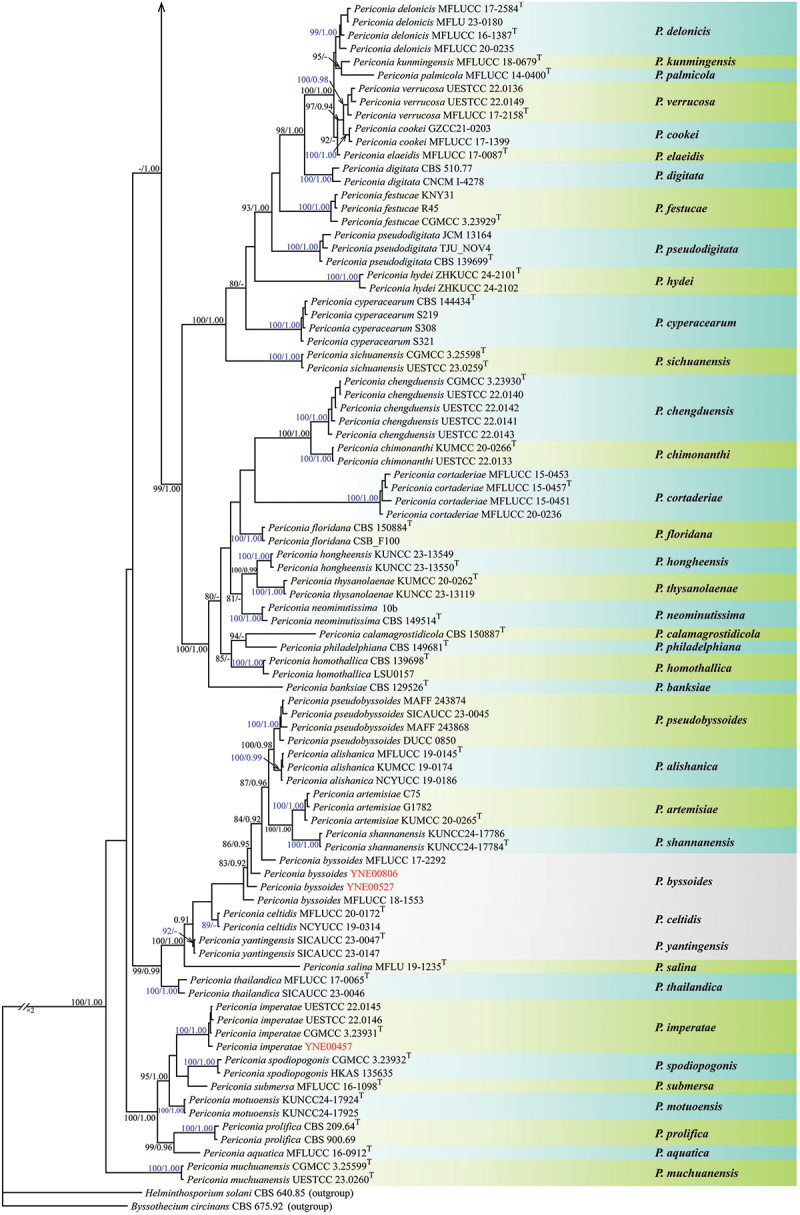
(Continued).

**Figure 3.**
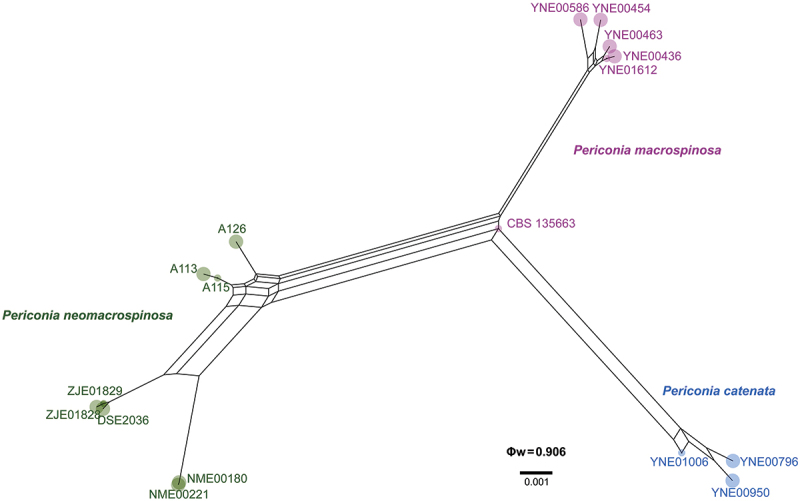
Split network of PHI test from the combined dataset for *Periconia macrospinosa* s.l., based on the LogDet transformation and the NeighborNet algorithm. PHI index test results (Φw > 0.05) indicate no statistically significant recombination within the datasets (LSU, ITS, *ACT*, *RPB2*, *TEF1*, and *TUB2*).

A notable case was observed in *P*. *macrospinosa* s.l., which was split into three distinct lineages in the multilocus analyses. Because strains assigned to *P*. *macrospinosa* s.l. formed a poorly resolved clade in the rDNA tree (Figure S4), yet were separated with strong support in other single-locus gene trees (Figures S5–S8), targeted species delimitation was undertaken. The dataset was restricted to *P*. *macrospinosa* s.l.; single-locus and concatenated alignments were generated; and split networks were inferred using LogDet transformation and NeighborNet algorithms to establish species boundaries. PHI tests indicated no evidence of recombination (Φw > 0.05) across loci: Φw = 0.906 for the concatenated LSU, ITS, *ACT*, *RPB2*, *TEF1*, and *TUB2* dataset (Figure 3), and Φw = 0.8332, 0.366, 0.3051, and 0.5314 for *ACT, RPB2*, *TEF1*, and *TUB2*, respectively (Figures S9–S12). These results corroborated three well-supported clades within *P. macrospinosa* s.l. By integrating molecular evidence with morphology, three lineages were recognised as *P. macrospinosa* s.s., *P. catenata*, and *P. neomacrospinosa*.

Beyond these redefined lineages, two additional, strongly supported clades (100% MLBP/1.00 BIPP) were formed by strains YNE01202, YNE01204, and YNE01208, and by strains YNE01205, YNE01210, and YNE01211, respectively, representing two putative new *Periconia* species. Furthermore, three endophytic strains collected in this study (NME00303, NME00313, and NME00323) clustered with the previously described *F. fueloephazae* in a distinct, strongly supported *Periconia* clade; to maintain genus monophyly, *F. fueloephazae* is proposed as *P*. *fueloephazae* comb. nov. In addition, five newly collected endophytic strains (YNE01674 and YNE01688; YNE00594 and YNE01142; YNE00457) clustered with *P. cynodontis*, *P. imperatae*, and *P. yunnanensis*, respectively, thereby confirming their identities as known species.

On the basis of combined phylogenetic and morphological evidence, the 28 *Periconiaceae* strains analysed here were distributed across 10 species (Figure 2): four known species (*P. byssoides*, *P. cynodontis*, *P. imperatae*, and *P. yunnanensis*), one new combination (*P*. *fueloephazae* comb. nov.), one sensu stricto taxon (*P*. *macrospinosa* s.s.), three new species (*P*. *catenata* sp. nov., *P*. *longibrachiatum* sp. nov., and *P*. *neomacrospinosa* sp. nov.), and one undescribed *Periconia* species due to the absence of sporulation.

### 3.3. Taxonomy

***Proxiconiothyrium*** C.L. Zhang & K.Y. Chen, gen. nov.

Fungal Names: FN 572681.

Etymology: The generic name refers to its phylogenetic and morphological proximity to *Coniothyrium*.

Endophytic within the leaves of *Oryza meyeriana* subsp. *granulata*. Sexual morph: Undetermined. Asexual morph: Mycelium hyaline to brown, septate, smooth. Conidiomata pycnidial, immersed or semi-immersed, globose or subglobose, dark brown to black, solitary or aggregated. Conidiophores absent. Conidiogenous cells lining the inner cavity, ampulliform, hyaline. Conidia aseptate, ovoid to ellipsoid, with obtuse apices, smooth, initially hyaline, pale brown when mature.

Type species: *Proxiconiothyrium yunnanense* C.L. Zhang & K.Y. Chen.

Notes: The strain YNE01575 from this study clustered with a previously misidentified strain (‘*Didymosphaeria*’ sp. ARM1124), forming a well-supported and distinct clade (D9, Figure 1, 100% MLBP/1.00 BIPP) closely related to the genus *Paraconiothyrium* (Figure 1). This relationship was consistently supported by single-locus phylogenetic analyses of the rDNA, *RPB2*, and *TEF1* loci (Figures S1–S3). The establishment of the new genus *Proxiconiothyrium* is necessary to classify this lineage. Morphologically, the conidia of this genus resemble those of *Paraconiothyrium*; however, *Paraconiothyrium* species occasionally possess a septum, whereas *Proxiconiothyrium* has aseptate conidia.

**Figure 4.**
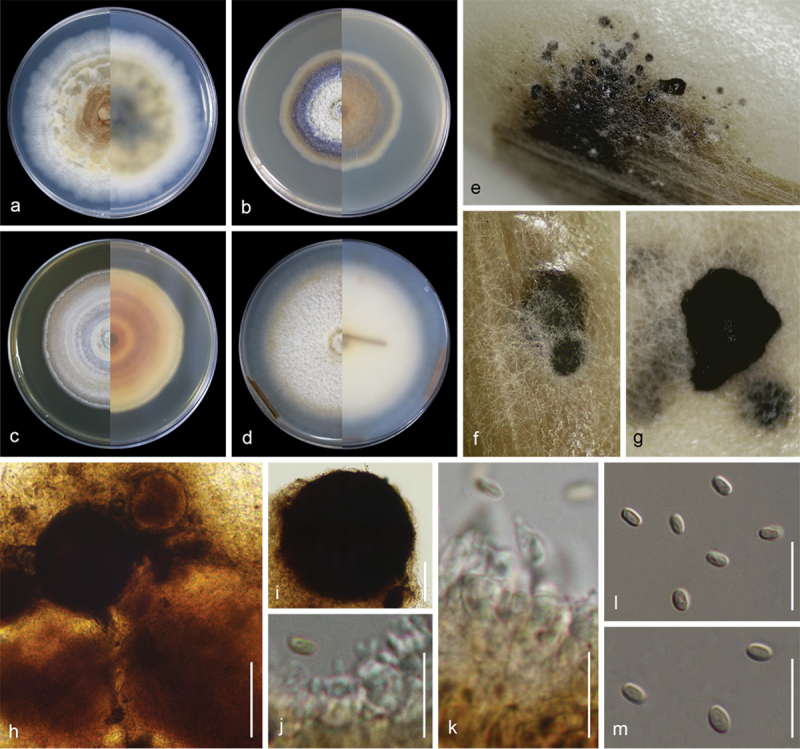
Morphological characterization of *Proxiconiothyrium yunnanense* (GDMCC 3.1186). (a–d) Upper and reverse views of colonies on different media (a: PDA; b: MMN; c: MEA; d: SNA). (e–g) Pycnidia on *Poaceae* leaves and SNA. (h, i) Pycnidia. (j, k) Conidiogenous cells. (l, m) Conidia. Scale bars: h, i = 100 μm; j–m = 10 μm.

***Proxiconiothyrium yunnanense*** C.L. Zhang & K.Y. Chen, sp. nov., Figure 4

Fungal Names: FN 572682.

Etymology: The specific epithet refers to the type location.

Sexual morph: Undetermined. Asexual morph: Conidiomata pycnidial, immersed or semi-immersed, globose or subglobose, up to 400 μm in diam., dark brown to black, solitary or aggregated. Conidiophores absent. Conidiogenous cells lining the inner cavity, ampulliform, hyaline, 4.5–7.5 × 2–3.5 μm. Conidia aseptate, ovoid to ellipsoid, 2.7–4 × 1.9–2.5 μm ($\overset{ˉ}{x}$ = 3.5 × 2.1 μm, *n* = 30; L/W ratio = 1.6), both ends obtuse, smooth, initially hyaline, pale brown when mature.

Culture characteristics: Colonies on PDA reaching 73 mm diam. after 21 d at 25 ℃, brown to greige with irregular white edges; reverse grey to greyish-yellow, white at the margin. Colonies on MMN medium sparse, surface slightly rough, dusty lavender to light taupe, greyish brown at the margin and pale grey in the centre, forming concentric rings; reverse brown to dark grey. Colonies on MEA flattened, greyish blue to beige, forming concentric rings; reverse rosy brown to cream. On SNA, colonies pale grey to light olive, cottony, with moderate aerial mycelium; reverse beige.

Material examined: China, Yunnan Province, Xishuangbanna, Naban River Watershed National Nature Reserve, isolated from the leaves of *Oryza meyeriana* subsp. *granulata*, October 2018, C.L. Zhang YNE01575 (Holotype GDMCC 3.1186, stored in a metabolically inactive state), ex-type living culture GDMCC 3.1186 = YNE01575.

Notes: Based on a sequence similarity search against the NCBI GenBank nucleotide database using the ITS sequence of strain YNE01575, the closest hits include ‘*Didymosphaeria*’ sp. ARM 1124 [GenBank no. PP277137; Identities = 510/510 (100%), Gaps = 0/510 (0%)], ‘*Coniothyrium juniperi*’ CBS 610.72 [GenBank no. MH860594; Identities = 504/521 (97%), Gaps = 9/521 (1%)], *Didymosphaeria variabile* CBS 121163 [GenBank no. NR_137006; Identities = 504/522 (97%), Gaps = 10/522 (1%)], ‘*Paraconiothyrium hakeae*’ CBS 142521 [GenBank no. NR_171729; Identities = 456/462 (99%), Gaps = 0/462 (0%)]. Morphologically, the conidia of this species resemble *Paraconiothyrium estuarinum* in shape but approximate those of *Didymosphaeria variabile* in size.

**Figure 5.**
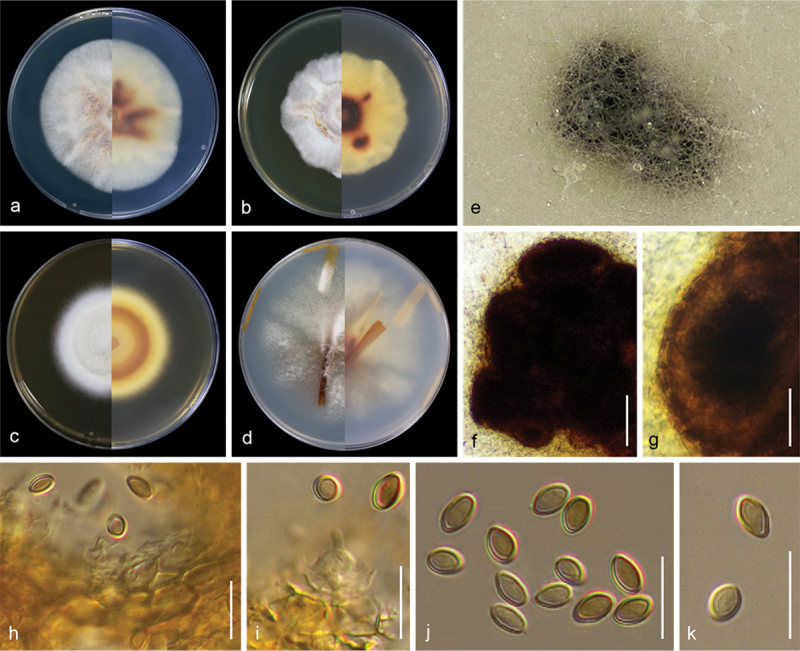
Morphological characterization of *Neptunomyces yunnanensis* (GDMCC 3.1182). (a–d) Upper and reverse views of colonies on different media (a: PDA; b: MMN; c: MEA; d: SNA). (e) Pycnidia covered by mycelium on SNA. (f, g) Pycnidia. (h, i) Conidiogenous cells and conidia. (j, k) Conidia. Scale bars: f = 100 μm; g = 20 μm; h–k = 10 μm.

***Neptunomyces yunnanensis*** C.L. Zhang & K.Y. Chen, sp. nov., Figure 5

Fungal Names: FN 572678.

Etymology: The specific epithet refers to the type location.

Sexual morph: Undetermined. Asexual morph: Conidiomata pycnidial, immersed, covered by dense mycelium, thick-walled, subglobose, up to 400 μm in diam., dark brown to black, solitary or aggregated. Conidiophores absent. Conidiogenous cells lining the inner cavity, ampulliform, phialidic, hyaline, 4.5–6 × 3.9–4.2 µm. Conidia aseptate, ellipsoid to fusoid, 3.9–6 × 2.6–3.9 μm ($\overset{ˉ}{x}$ = 5.2 × 3.8 μm, *n* = 30; L/W ratio = 1.6), smooth, initially hyaline and soon becoming golden brown.

Culture characteristics: Colonies on PDA reaching 64 mm diam. after 14 d at 25 ℃, pale brown to white, with aerial mycelium raised in the centre; reverse brown to pale greyish-yellow. Colonies on MMN produce sparse exudate droplets, pale greyish red to white, with irregular edges; reverse deep reddish-brown to beige-yellow. Colonies on MEA moderate density, white; reverse peru to beige-yellow, forming concentric rings. Colonies on SNA grey to white, with sparse aerial mycelium; reverse pale grey to greyish-yellow.

Material examined: China, Yunnan Province, Xishuangbanna, Naban River Watershed National Nature Reserve, isolated from the leaves of *Oryza meyeriana* subsp. *granulata*, October 2018, C.L. Zhang YNE00523 (Holotype GDMCC 3.1182, stored in a metabolically inactive state), ex-type living culture GDMCC 3.1182 = YNE00523.

Notes: Based on a sequence similarity search against the NCBI GenBank nucleotide database using the ITS sequence of strain YNE00523, the closest hits include *Neptunomyces litoralis* BRIP 75555a [GenBank no. NR_189984; Identities = 506/517 (98%), Gaps = 2/517 (0%)], *Neptunomyces juncicola* CBS 150790 [GenBank no. NR_197932; Identities = 500/519 (96%), Gaps = 7/519 (1%)], *Neptunomyces* sp. SFC102397 [GenBank no. MH374562; Identities = 517/517 (100%), Gaps = 0/517 (0%)], *Neptunomyces* sp. zzz714 [GenBank no. HQ696074; Identities = 517/517 (100%), Gaps = 0/517 (0%)]. In the multilocus phylogenetic tree (Figure 1), YNE00523 grouped with two unidentified *Neptunomyces* strains (SFC102397 and zzz714), forming a unique cluster (100% MLBP/1.00 BIPP) within *Neptunomyces* (Figure 1). Morphologically, this species can be differentiated from *Neptunomyces aureus* by its relatively broader conidia (5.2 × 3.8 μm vs. 7.0 × 2.7 μm in *Neptunomyces aureus*) (Gonçalves et al. 2019).

***Chlamydosphaeromyces*** C.L. Zhang & K.Y. Chen, gen. nov.

Fungal Names: FN 572675.

Etymology: The generic name refers to the chlamydospores that form spherical structures.

Sexual morph: Undetermined. Asexual morph: Mycelium predominantly hyaline, sometimes brown and septate. Chlamydospores hyaline to dark brown, intercalary or terminal, globose to subglobose. Abundant chlamydospores aggregate into spherical clusters, mostly submerged in the agar, dark brown to black.

Type species: *Chlamydosphaeromyces glomeratus* C.L. Zhang & K.Y. Chen.

Notes: *Pseudopithomyces chartarum* (syn. *Leptosphaerulina chartarum*), the type species of *Pseudopithomyces*, clusters with *Pseudopithomyces kunmingensis* and *Pseudopithomyces mori*, forming a distinct clade (D14, Figure 1). Two endophytes from this study, namely, YNE00904 and YNE00913, form a distinct clade (D32, Figure 1) with two previously misidentified strains (‘*Leptosphaerulina chartarum*’ C2-227 and ‘*Leptosphaerulina* cf. *chartarum*’ 72C), resulting in the introduction of a new genus and a new species (*Chlamydosphaeromyces glomeratus*) to accommodate them.

**Figure 6.**
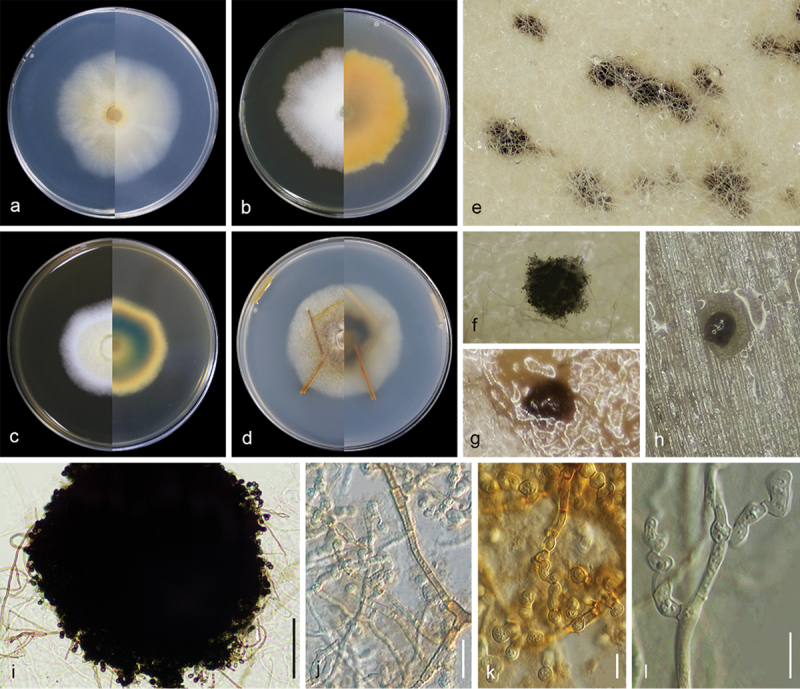
Morphological characterization of *Chlamydosphaeromyces glomeratus* (GDMCC 3.1185). (a–d) Upper and reverse views of colonies on different media (a: PDA; b: MMN; c: MEA; d: SNA). (e–h) Chlamydospores aggregating into clusters on SNA, MMN, MEA, and *Poaceae* leaves. (i) Chlamydospores aggregating into spherical cluster. (j–l) Chlamydospores. Scale bars: i = 100 μm; j = 20 μm; k, l = 10 μm.

***Chlamydosphaeromyces glomeratus*** C.L. Zhang & K.Y. Chen, sp. nov., Figure 6

Fungal Names: FN 572676.

Etymology: Species epithet ‘*glomeratus*’ denotes the globose aggregation of chlamydospores.

Sexual morph: Undetermined. Asexual morph: Most hyphae hyaline, with some brown and septate. Chlamydospores (5–9 μm in width) in hyphae were observed after 4 weeks on MEA, MMN, and SNA, hyaline to dark brown, and aggregated into dark brown to black spherical clusters.

Culture characteristics: Colonies on PDA reaching 52 mm diam. after 14 d at 25 °C, light taupe to white with sparse aerial mycelium; reverse pale greyish yellow. On MMN, colonies white, less fluffy, with a taupe marginal zone; reverse amber. Colonies on MEA pale yellow to white, hairy at the margin; reverse dark slate grey to light brown, forming concentric rings. On SNA, colonies pale grey at the margin and light brown to khaki in the centre; reverse dark grey to greyish yellow, with a pale grey marginal zone.

Material examined: China, Yunnan Province, Xishuangbanna, Naban River Watershed National Nature Reserve, isolated from the leaves of *Poaceae* sp., October 2018, C.L. Zhang YNE00913 (Holotype GDMCC 3.1185, stored in a metabolically inactive state), ex-type living culture GDMCC 3.1185 = YNE00913.

Notes: Based on a sequence similarity search against the NCBI GenBank nucleotide database using the ITS sequence of strain YNE00913, the closest hits include *Agrorhizomyces patris* CBS 151043 [GenBank no. NR_197907; Identities = 344/382 (90%), Gaps = 13/382 (3%)], *Dictyoarthrinium hydei* SQUCC 13296 [GenBank no. NR_173309; Identities = 374/430 (87%), Gaps = 21/430 (4%)], ‘*Leptosphaerulina chartarum’* strain C2-227 [GenBank no. KJ439067; Identities = 456/468 (97%), Gaps = 4/468 (0%)], ‘*Leptosphaerulina* cf. *chartarum*’ 72C [GenBank no. KF555232; Identities = 297/307 (97%), Gaps = 1/307 (0%)]. Phylogenetically, new collections (YNE00904 and YNE00913) clustered together, forming a distinct subclade in Clade D32 (Figure 1; 100% MLBP/1.00 BIPP), and are closely related to two previously misidentified strains (‘*Leptosphaerulina chartarum*’ C2-227 and ‘*Leptosphaerulina* cf. *chartarum*’ 72C). Morphologically, while no sporulating structures were induced, the strains of this species produced clustered chlamydospores on MEA, MMN, and SNA media (Figure 6).

***Paraconiothyrium zingiberacearum*** Tennakoon & S. Lumyong, in Tennakoon et al. *Frontiers in Microbiology* 13(101628): 16 (2023)

= *Paraconiothyrium marshiae* Y.P. Tan, Bishop-Hurley & R.G. Shivas, in Y.P. Tan & R.G. Shivas, *Index of Australian Fungi* 41: 16 (2024).

Notes: Phylogenetically, YNE00613 and YNE01332 from this study clustered with *Paraconiothyrium zingiberacearum* (NCYUCC 19-0230, type strain), and ‘*Paraconiothyrium marshiae*’ (BRIP 75785a, type strain), forming a distinct monophyletic subclade within *Paraconiothyrium* (D8, Figure 1) with robust support, indicating that they are the same species. *Paraconiothyrium zingiberacearum* Tennakoon & S. Lumyong [in Frontiers in Microbiology 13(101628): 16. 2023] has priority over *Paraconiothyrium marshiae* Y.P. Tan, Bishop-Hurley & R.G. Shivas (in Index of Australian Fungi 41: 16. 2024).

***Periconia macrospinosa*** Lefebvre & Aar.G. Johnson, in Lefebvre et al., *Mycologia* 41 (4): 417 (1949) Figure 7

**Figure 7.**
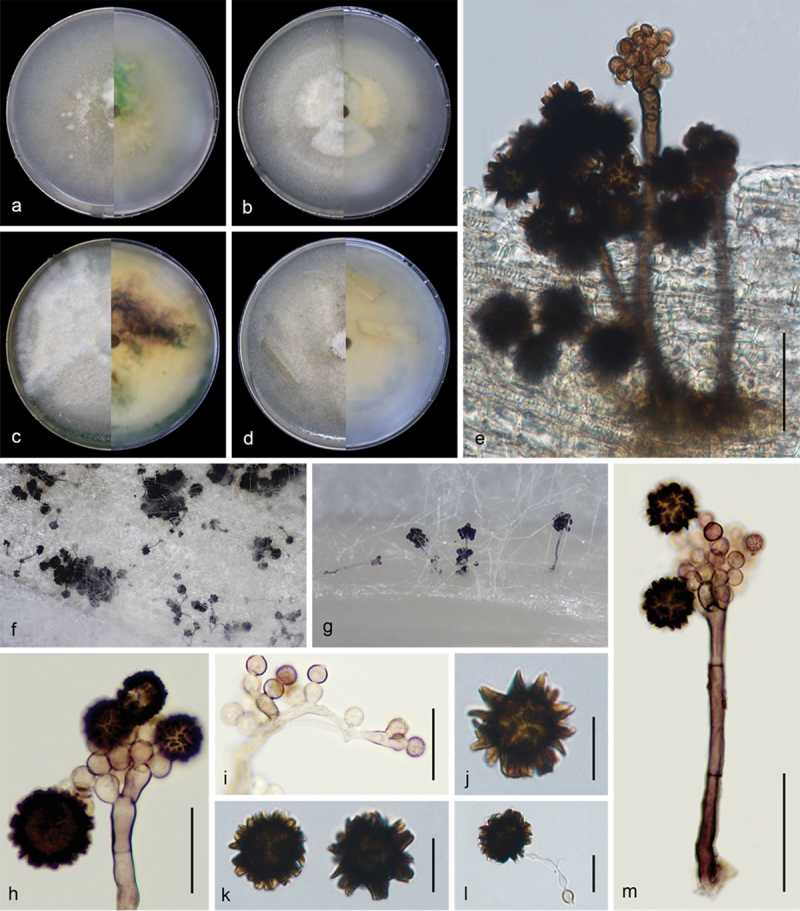
Morphological characterization of *Periconia macrospinosa* s.s. (YNE00586). (a–d) Upper and reverse views of colonies on different media (a: PDA; b: MMN; c: MEA; d: SNA). (e) Clumped conidiophores. (f, g) Conidia sporulated on medium. (h) Conidiogenous cells and conidia. (i) Conidiogenous cells. (j–l) Conidia. (m) Conidiophore with conidiogenous cells and conidia. Scale bars: e, m = 50 μm; h, i = 25 μm; j–l = 20 μm.

Index Fungorum number: IF289149; Facesoffungi number: FoF 289149.

Sexual morph: Undetermined. Asexual morph: Mycelium hyaline to pale brown, septate, smooth to verruculose, and hyphae 3–6 µm wide. Conidiophores arising singly from thickened hyphae, brown, septate, upright or slightly curved, up to 420 μm long; branched inside the head, 6.7–12 μm thick at the base, and 6–10 µm wide just below the head. Conidiogenous cells arising from the swollen apex of the conidiophore, spherical or ovoid, 7–12 × 5–9 μm, pale to mid brown, smooth, often accompanied by smaller secondary conidiogenous cells. Conidia spherical, echinulate, 18–35 μm diam., dark brown to black; the spines sometimes adhere closely together in groups, 2–7 µm long.

Culture characteristics: Colonies on PDA reaching 70.5 mm diam. after 10 d at 25 ℃, white to pale grey with sparse aerial mycelium; reverse pale cream at the margin and green in the centre. On MMN, colonies moderately dense, forming white mycelial turfs on the surface; reverse pale cream with white margin. Colonies on MEA similar to PDA but with abundant aerial mycelium; reverse pale yellow, some edges dull green with brown and greenish grey mixed in the centre. Colonies on SNA initially white and becoming pale grey over time; reverse cream with a white margin.

Additional material examined: China, Yunnan Province, Xishuangbanna, Naban River Watershed National Nature Reserve, isolated from the roots of *Oryza meyeriana* subsp. *granulata*, October 2016, C.L. Zhang YNE00586; ibid., isolated from the roots of *Oryza meyeriana* subsp. *granulata*, October 2016, C.L. Zhang YNE00436; ibid., isolated from the roots of *Oryza meyeriana* subsp. *granulata*, October 2016, C.L. Zhang YNE00454; ibid., isolated from the roots of *Oplismenus* sp., October 2016, C.L. Zhang YNE00463; ibid., isolated from the roots of *Poaceae* sp., October 2018, C.L. Zhang YNE01612.

Notes: Based on a sequence similarity search against the NCBI GenBank nucleotide database using the ITS sequence of strain YNE00586, the closest hits include *P. endophytica* ZHKUCC 23-0995 [GenBank no. OR995582; Identities = 451/463 (97%); Gaps = 4/463 (0%)], *P*. *ananasi* MFLUCC 21-0155 [GenBank no. NR_190247; Identities = 453/467 (97%); Gaps = 6/467 (1%)]. In the multilocus phylogenetic tree (Figure 2), YNE00586, along with several other new collections in this study (YNE00436, YNE00454, YNE00463, YNE01612, YNE01006, YNE00796, YNE00950, ZJE01828, ZJE01829, NME00180, NME00221), clustered together with six strains of *P*. *macrospinosa* s.l. (CBS 135663, DSE2036, TU41, A113, A115, A126), forming a distinct group closely related to *P*. *circinata*. Notably, YNE00586, YNE00436, YNE00454, YNE00463, YNE01612, and *P*. *macrospinosa* CBS 135663 constitute a well-supported clade (100% MLBP/1.00 BIPP) within this phylogenetic framework.

*Periconia macrospinosa* was initially established by Lefebvre et al. (1949) and can be readily distinguished from *P*. *circinata* by its straight conidiophores (not circinate) and larger conidia (18–35 μm diam.). Morphologically, strain YNE00586 in this study closely resembles *P*. *macrospinosa* described by Lefebvre et al. (1949), featuring larger conidia (18–32.5 μm diam.) with spiny protrusions, while some strains of other clades, such as A113, A115, and A126, displayed comparatively smaller conidial diameters (8.3–17.6 μm) (Fors et al. 2020). Therefore, although sequence data for the earliest *P*. *macrospinosa* are absent, this clade (including CBS 135663, YNE00586, YNE00436, YNE00454, YNE00463, and YNE01612) is designated as *P*. *macrospinosa* s.s. *Periconia macrospinosa* s.s. can be distinguished from the other two species (*P*. *catenata* and *P*. *neomacrospinosa*) by its capacity to produce green pigments in culture (Figure 7), along with its characteristically larger conidia bearing longer spines (see the notes on *P*. *catenata* and *P*. *neomacrospinosa*).

**Figure 8.**
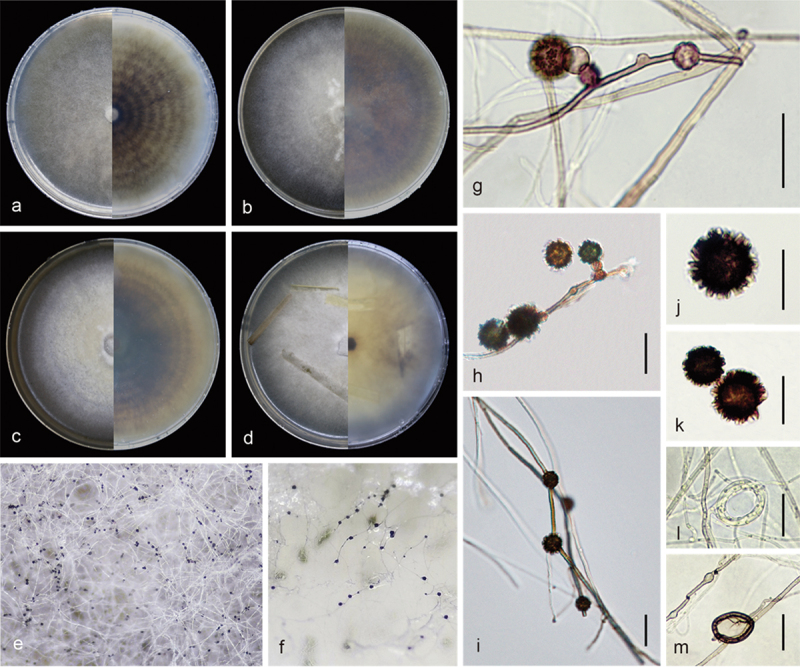
Morphological characterization of *Periconia catenata* (GDMCC 3.1041). (a–d) Upper and reverse views of colonies on different media (a: PDA; b: MMN; c: MEA; d: SNA). (e, f) Conidia sporulated on medium. (g–i) Conidiogenous cells and conidia on hyphae. (j, k) conidia. (l, m) Ring-like structures. Scale bars: g, l, m = 25 μm; h–k = 20 μm.

***Periconia catenata*** C.L. Zhang & K.Y. Chen, sp. nov., Figure 8

Fungal Names: FN 571878.

Etymology: Named after the production of catenate spores in hyphae.

Sexual morph: Undetermined. Asexual morph: Mycelium hyaline to light brown, often forming ring-like structures. Conidiophores not observed. Conidiogenous cells forming directly on centrally swollen, light brown hyphae, spherical to ovoid. Conidia catenate on the hyphae, spherical, verruculose to shortly echinulate, 14–22.5 μm ($\overset{ˉ}{x}$ = 18.7 μm, *n* = 30) in diam., olive gray initially and dark brown when mature; spines short and truncate at the apex, 1–3.8 μm long.

Culture characteristics: Colonies on PDA reaching 69.5 mm in diam. after 10 d at 25 ℃, olivaceous-gray with moderately dense aerial mycelium; reverse dark brown-grey with the formation of concentric rings. On both MMN and MEA, colonies pale gray; reverse brown to dark brown-gray, forming concentric rings. On SNA, colonies white to pale gray with moderately dense aerial mycelium; reverse pale cream at the margin and pale yellow in the centre.

Material examined: China, Yunnan Province, Xishuangbanna, Naban River Watershed National Nature Reserve, isolated from the roots of *Capillipedium* sp., Oct. 2016, C.L. Zhang (Holotype GDMCC 3.1041, stored in a metabolically inactive state), ex-type living culture GDMCC 3.1041 = YNE00950.

Additional material examined: China, Yunnan Province, Xishuangbanna, Naban River Watershed National Nature Reserve, isolated from the roots of *Capillipedium* sp., October 2016, C.L. Zhang YNE00796; ibid., isolated from the roots of *Poaceae* sp., October 2018, C.L. Zhang YNE01006.

Notes: Based on a sequence similarity search against the NCBI GenBank nucleotide database using the ITS sequence of strain YNE00950, the closest hits include *P. endophytica* ZHKUCC 23-0995 [GenBank no. OR995582; Identities = 450/463 (97%); Gaps = 4/463 (0%)], *P*. *ananasi* MFLUCC 21-0155 [GenBank no. NR_190247; Identities = 452/467 (97%); Gaps = 6/467 (1%)]. In our multilocus phylogenetic tree, YNE00950, along with YNE01006 and YNE00796, forms a unique cluster within the *P*. *macrospinosa* s.l. branch (100% MLBP/1.00 BIPP) and is closely related to *P*. *circinata* (Figure 2). Morphologically, the new strains can be differentiated from *P*. *circinata* and *P*. *ananasi* by the absence of conidiophores and by catenate conidia (Fors et al. 2020; Tian et al. 2022). In comparison to *P*. *macrospinosa* s.s., this species exhibits smaller conidia (14–22.5 μm vs. 18–35 μm in *P. macrospinosa* s.s.) and shorter spines (1–3.8 μm vs. 2–7 μm in *P. macrospinosa* s.s.) on the conidial surface. Thus, the new strains are identified as *P*. *catenata* sp. nov. based on phylogenetic analyses and morphological characteristics.

**Figure 9.**
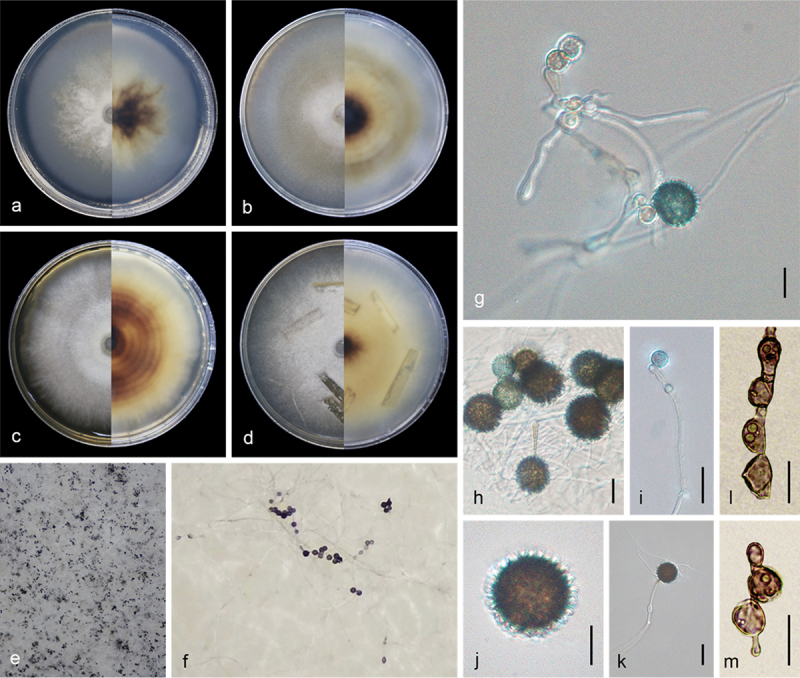
Morphological characterization of *Periconia neomacrospinosa* (GDMCC 3.1042). (a–d) Upper and reverse views of colonies on different media (a: PDA; b: MMN; c: MEA; d: SNA). (e, f) Conidia sporulated on medium. (g–i) Conidiogenous cells and conidia on hyphae. (j, k) Conidia. (l, m) Chlamydospores. Scale bars: g, h, j = 10 μm; i, l, k, m = 20 μm.

***Periconia neomacrospinosa*** C.L. Zhang & K.Y. Chen, sp. nov., Figure 9

Fungal Names: FN 571879.

Etymology: Named after its close phylogenetic relationship to *P*. *macrospinosa* s.s.

Sexual morph: Undetermined. Asexual morph: Mycelium mostly hyaline, forming catenate chlamydospore-like brown structures. Conidia solitary or catenate on the hyphae, sometimes gathering in clusters. Conidiophores either short [11–157 μm ($\overset{ˉ}{x}$ = 50.1 μm, *n* = 6) long] or absent. Conidiogenous cells forming on swollen hyphae, spherical, hyaline to pale olive. Conidia spherical, verruculose, 15.5–24 μm ($\overset{ˉ}{x}$ = 19 μm, *n* = 30) in diam., dark brown when mature; spines short and truncate at the apex, 1–3.3 μm long.

Culture characteristics: Colonies on PDA reaching 65 mm diam. after 14 d at 25 ℃, white to pale grey, flattened at the margin and cottony in the centre; reverse cream to brown-grey with a white marginal zone. On MMN, colonies white to pale khaki with the formation of concentric rings; reverse featuring four concentric rings of different colours-white, pale khaki, pale yellow, and dark brown, from the outermost to the innermost ring. Colonies on MEA hairy, pale grey at the margin and white in the centre; reverse brown to pale yellow, forming distinct concentric rings. On SNA, colonies cottony, pale grey at the margin and white in the centre; reverse pale yellow with a dark brown centre.

Material examined: China, Inner Mongolia, Xilinhaote, isolated from the roots of *Cleistogenes serotina*, Sept. 2016, C.L. Zhang (Holotype GDMCC 3.1042, stored in a metabolically inactive state), ex-type living culture GDMCC 3.1042 = NME00221.

Additional material examined: China, Inner Mongolia, Xilinhaote, isolated from the roots of *C*. *serotina*, Sept. 2016, C.L. Zhang, NME00180. China, Zhejiang Province, Baishanzu National Nature Reserve, isolated from the roots of *Miscanthus sinensis*, Oct. 2018, C.L. Zhang, ZJE01828; ibid., isolated from the roots of *M*. *sinensis*, Oct. 2018, C.L. Zhang, ZJE01829.

Notes: Based on a sequence similarity search against the NCBI GenBank nucleotide database using the ITS sequence of strain NME00221, the closest hits include *P. endophytica* ZHKUCC 23-0995 [GenBank no. OR995582; Identities = 451/463 (97%); Gaps = 4/463 (0%)]), *P*. *ananasi* MFLUCC 21-0155 [GenBank no. NR_190247; Identities = 453/467 (97%); Gaps = 6/467 (1%)]. Phylogenetically, our four new collections (ZJE01828, ZJE01829, NME00180, NME00221) cluster together with five *P*. *macrospinosa* s.l. strains (DSE2036, TU41, A113, A115, A126) with robust support (100% MLBP/1.00 BIPP) (Figure 2). Morphologically, this species can be distinguished from *P*. *macrospinosa* s.s. by smaller conidia (15.5–24 μm vs. 18–35 μm in *P*. *macrospinosa* s.s.) and shorter spines (1–3.3 μm vs. 2–7 μm in *P*. *macrospinosa* s.s.) on the conidial surface. However, morphological diversity was also observed within this clade. NME00221 predominantly produced conidia on swollen hyphae; DSE2036 featured solitary conidiophore stalks [as shown by Knapp et al. (2018)]; A113, A115, and A126 had either solitary or clustered conidiophore stalks (Fors et al. 2020); and our strain ZJE01829 encompassed all three of these characteristics. Ultimately, this species is designated as *P. neomacrospinosa* sp. nov.

**Figure 10.**
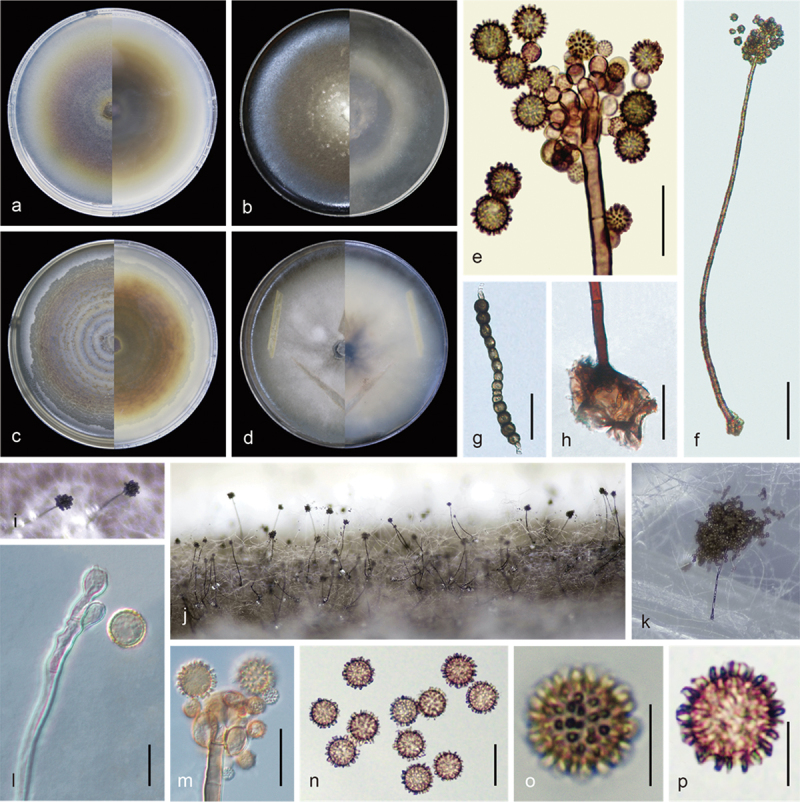
Morphological characterization of *Periconia longibrachiatum* (GDMCC 3.1043). (a–d) Upper and reverse views of colonies on different media (a: PDA; b: MMN; c: MEA; d: SNA). (e) Conidial head bearing conidiogenous cells and conidia. (f) Conidiophore with conidiogenous cells and conidia. (g) Bead-like dark structure. (h) Base of conidiophore. (i–k) Conidia sporulated on medium. (l, m) Conidiogenous cells. (n–p) Conidia. Scale bars: e = 25 μm; f = 100 μm; g–h = 50 μm; l–n = 20 μm; o–p = 10 μm.

***Periconia longibrachiatum*** C.L. Zhang & K.Y. Chen, sp. nov., Figure 10

Fungal Names: FN 571881.

Etymology: longibrachiatum, referring to the long conidiophores.

Sexual morph: Undetermined. Asexual morph: Mycelium mostly hyaline, with some brown and septate, forming catenate bead-like dark structures. Conidiophores brown, dark brown at the base, separate, upright or slightly curved, 310–655 μm ($\overset{ˉ}{x}$ = 422.2 μm, *n* = 3) long, branched inside the head, 6.7–10.2 μm ($\overset{ˉ}{x}$ = 8.2 μm, *n* = 3) wide. Conidiogenous cells arising from the swollen apex of the conidiophore, spherical or ovoid, 6–10.3 × 5.6–7.8 μm ($\overset{ˉ}{x}$ = 7.8 × 6.9 μm, *n* = 15), pale brown to brown, often accompanied by smaller secondary conidiogenous cells. Conidia spherical, echinulate, 10.5–16.8 μm ($\overset{ˉ}{x}$ = 13.7 μm, *n* = 30) in diam., golden brown to brown.

Culture characteristics: Colonies on PDA reaching 70.5 mm diam. after 10 d at 25 ℃, white to pale grey with sparse aerial mycelium; reverse pale cream at the margin and green in the centre. On MMN, colonies moderately dense, forming white mycelial turfs on the surface; reverse pale cream with a white margin. Colonies on MEA similar to PDA but with abundant aerial mycelium; reverse pale yellow, some edges dull green with brown and greenish grey mixed in the centre. Colonies on SNA initially white and become pale grey over time; reverse cream with white margin.

Material examined: China, Yunnan Province, Xishuangbanna, Naban River Watershed National Nature Reserve, isolated from the roots of *Poaceae* sp., Oct. 2018, C.L. Zhang YNE01208 (Holotype GDMCC 3.1043, stored in a metabolically inactive state), ex-type living culture GDMCC 3.1043 = YNE01208.

Additional material examined: China, Yunnan Province, Xishuangbanna, Naban River Watershed National Nature Reserve, isolated from the roots of *Poaceae* sp., October 2018, C.L. Zhang YNE01202; ibid., isolated from the roots of *Poaceae* sp., October 2018, C.L. Zhang YNE01204.

Notes: Based on a sequence similarity search against the NCBI GenBank nucleotide database using the ITS sequence of strain YNE01208, the closest hits include *P. endophytica* ZHKUCC 23-0995 [GenBank no. OR995582; Identities = 449/463 (97%); Gaps = 4/463 (0%)], *P*. *ananasi* MFLUCC 21-0155 [GenBank no. NR_190247; Identities = 450/467 (96%); Gaps = 6/467 (1%)]. In our multilocus phylogenetic tree, three new collections (YNE01202, YNE01204, YNE01208) form a distinct branch (Clade 31, 100% MLBP/1.00 BIPP), which is closely related to *P*. *macrospinosa* s.l. and *P*. *circinata* (Figure 2). Morphologically, this species can be distinguished from *P. macrospinosa* s.l. by conidiophore length. Conidiophores of *P*. *longibrachiatum* (310–655 μm) are longer than those of *P*. *macrospinosa* s.s. (≤ 420 μm) and *P*. *neomacrospinosa* (11–157 μm), whereas *P*. *catenata* entirely lacks conidiophores. Additionally, chlamydospores resembling those of *P*. *circinata* were observed in this species (Figure 10g), yet they can be differentiated by the morphology of conidiophore apices (*P*. *circinata* circinating at the apex) (Zhao et al. 2021). Thus, the strains (YNE01202, YNE01204, YNE01208) are identified as a new species, *P*. *longibrachiatum*.

***Periconia cynodontis*** Z.Hua Lu, P.W. Su & Maharachch., in Su et al., *Journal of Fungi* 9(3, no. 300): 12 (2023)

Index Fungorum number: IF847465; Facesoffungi number: FoF847465.

Sexual morph: Undetermined. Asexual morph: See Su et al. (2023).

Additional material examined: China, Yunnan Province, Xishuangbanna, Naban River Watershed National Nature Reserve, isolated from the leaf sheath of *Oplismenus* sp., October 2016, C.L. Zhang YNE00594; ibid., isolated from the stem of *Poaceae* sp., October 2018, C.L. Zhang YNE01142.

**Figure 11.**
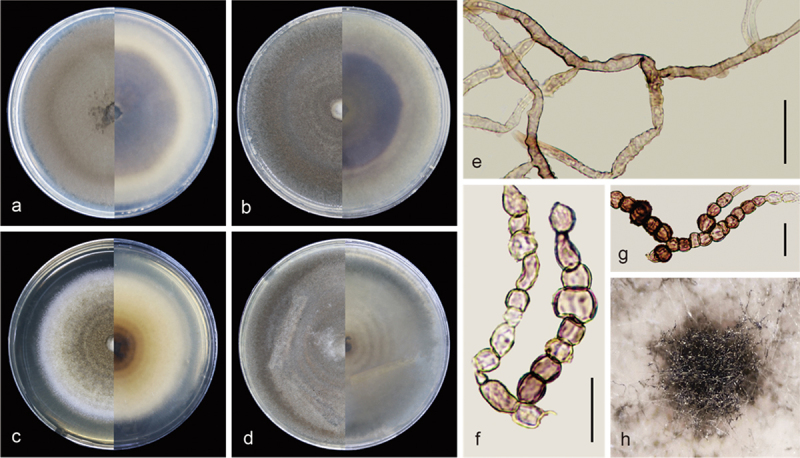
Morphological characterization of *Periconia cynodontis* (YNE00594). (a–d) Upper and reverse views of colonies on different media (a: PDA; b: MMN; c: MEA; d: SNA). (e) Hyphae with small bubble-like protrusions. (f, g) Chlamydospores. (h) Dark hyphae gathering into clusters on medium. Scale bars: e = 25 μm; f–g = 20 μm.

Notes: Based on a sequence similarity search against the NCBI GenBank nucleotide database using the ITS sequence of strain YNE00594, the closest hits include *P*. *cynodontis* CGMCC 3.23927 [GenBank no. NR_185795; Identities = 456/459 (99%); Gaps = 2/459 (0%)], *P. neominutissima* CBS 149514 [GenBank no. NR_189522; Identities = 405/432 (94%); Gaps = 4/432 (0%)]. In the multilocus phylogenetic tree (Figure 2), the new collections (YNE00594 and YNE01142) cluster with *P. cynodontis* strains (including the type CGMCC 3.23927), forming a distinct and well-supported clade. *Periconia cynodontis* was previously reported as saprobic on dead leaves of *Cynodon dactylon*, whereas strain YNE00594 was isolated as an endophyte from the leaf sheath of *Oplismenus* sp. Morphologically, the hyphae of this species are hyaline to brown, with small bubble-like protrusions on the surface, forming catenate chlamydospores (Figure 11). However, conidiophores, conidiogenous cells, and conidia were not observed in YNE00594 and YNE01142.

**Figure 12.**
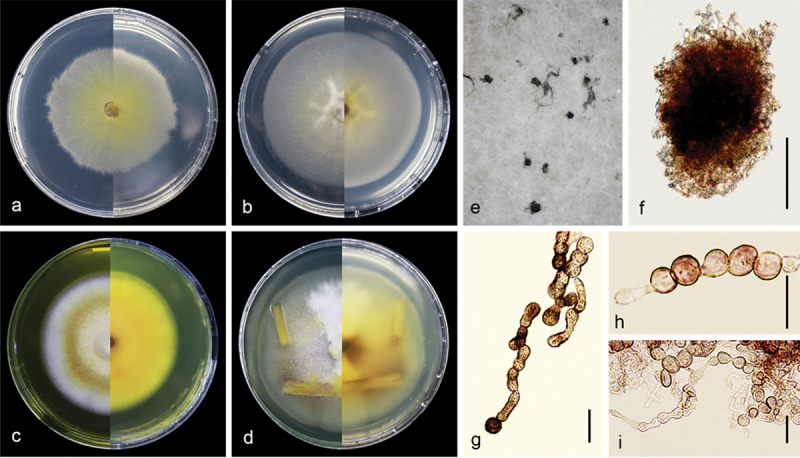
Morphological characterization of *Periconia fueloephazae* (NME00313). (a–d) Upper and reverse views of colonies on different media (a: PDA; b: MMN; c: MEA; d: SNA). (e, f) Dark hyphae gathering into clusters. (g–i) Chlamydospores. Scale bars: f = 50 μm; g–i = 20 μm.

***Periconia fueloephazae*** (D.G. Knapp, Kovács, J.Z. Groenew. & Crous), C.L. Zhang & K.Y. Chen, comb. nov., Figure 12

Basionym: *Flavomyces fueloephazae* D.G. Knapp, Kovács, J.Z. Groenew. & Crous, in Knapp et al., *Persoonia* 35: 93 (2015).

Index Fungorum number: IF 571884.

Sexual morph: Undetermined. Asexual morph: See Knapp et al. (2015).

Additional material examined: China, Inner Mongolia, Xilinhaote, isolated from the roots of *Cleistogenes squarrosa*, September 2016, C.L. Zhang, NME00313; ibid., isolated from the roots of *Cleistogenes squarrosa*, September 2016, C.L. Zhang, NME00303; ibid., isolated from the roots of *Cleistogenes squarrosa*, September 2016, C.L. Zhang, NME00323.

Notes: Based on a sequence similarity search against the NCBI GenBank nucleotide database using the ITS sequence of strain NME00313, the closest hits include *F*. *fueloephazae* CBS 135761 [GenBank no. NR_137960; Identities = 457/462 (99%); Gaps = 5/462 (1%)], *P*. *cynodontis* CGMCC 3.23927 [GenBank no. NR_185795; Identities = 400/423 (95%); Gaps = 1/423 (0%)]. The result of an LSU nucleotide BLAST indicated that the closest hits include *F*. *fueloephazae* CBS 135761 [GenBank no. NG_058131; Identities = 509/510 (99%); Gaps = 0/510 (0%)], *P*. *chimonanthi* KUMCC 20-0266 [GenBank no. NG_081512; Identities = 504/510 (99%); Gaps = 0/510 (0%)], *P. yangjiangensis* ZHKUCC 23-0997 [GenBank no. NG_243970; Identities = 502/510 (98%); Gaps = 0/510 (0%)], and *P*. *ananasi* MFLUCC 21-0155 [GenBank no. NG_243062; Identities = 498/507 (98%); Gaps = 0/507 (0%)]. Vu et al. (2019) proposed a classification threshold of 98.2% for the identification of filamentous fungal genera based on LSU barcodes. Therefore, it can be inferred that *F*. *fueloephazae* has a close relationship with *Periconia* species.

*Flavomyces fueloephazae* was introduced by Knapp et al. (2015) and can be readily distinguished by its capacity to produce yellow pigments on colonies. In the multilocus phylogenetic tree (Figure 2), the new collections (NME00303, NME00323, and NME00313) cluster with *F*. *fueloephazae* strains (including the type CBS 135761), forming a distinct and well-supported clade within the *Periconia* genus. Strain NME00313 in this study, similar to *F*. *fueloephazae* (CBS 135761) described by Knapp et al. (2015), was isolated as an endophyte from the roots of *Poaceae* and can produce yellow pigments (Figure 12a–d). Additionally, bead-like chlamydospores (Figure 12g–i) resembling those of *P*. *circinata* (Zhao et al. 2021) and *P*. *longibrachiatum* (Figure 10g) were observed. Therefore, *Flavomyces fueloephazae* is synonymised under *Periconia* as *P*. *fueloephazae* based on phylogenetic analyses and morphological characteristics.

***Periconia delonicis*** Jayasiri, E.B.G. Jones & K.D. Hyde, in Jayasiri et al., *Mycosphere* 10(1): 95 (2019)

= *Periconia arecacearum* S.N. Zhang, K.D. Hyde & Jian K. Liu, in Zhang et al., *Fungal Diversity* 127: 155 (2024).

Notes: Phylogenetically, *Periconia delonicis* strains [MFLUCC 17-2584 (type strain), MFLUCC 20-0235, and MFLU 23-0180] clustered with the *Periconia arecacearum* strain (MFLUCC 16-1387, type strain), forming a distinct monophyletic clade within *Periconia* with robust support (99% MLBP/1.00 BIPP), indicating that they are the same species. *Periconia delonicis* Jayasiri, E.B.G. Jones & K.D. Hyde [in Mycosphere 10(1): 95. 2019] has priority over *Periconia arecacearum* S.N. Zhang, K.D. Hyde & Jian K. Liu (in Fungal Diversity 127: 155. 2024).

## 4. Discussion

The classification and characterisation of endophytic fungi are fundamental for advancing our understanding of their diversity and evolutionary relationships (Knapp et al. 2015; Thambugala et al. 2017). In this study, 16 *Didymosphaeriaceae* and 28 *Periconiaceae* strains were examined, which were isolated as endophytes from *Poaceae* hosts. Analyses resulted in the proposal of 10 new taxa, including two new genera, six new species, one new combination, and one sensu stricto taxon.

*Didymosphaeriaceae* is a large and highly diverse family within *Pleosporales*. Prior to this study, 39 genera were recognised within *Didymosphaeriaceae* (Ariyawansa et al. 2014b; Maharachchikumbura et al. 2021b; Hyde et al. 2024; Pem et al. 2024). However, taxonomic uncertainties in *Didymosphaeriaceae* continue to persist, primarily due to limited molecular data in comparison to morphological studies (Ariyawansa et al. 2014a; Maharachchikumbura et al. 2021b). Many species still lack multilocus sequence data beyond ITS and LSU, which makes it challenging to robustly resolve their taxonomic boundaries. In this study, using combined multilocus sequence data (SSU, LSU, ITS, *RPB2*, *TEF1*), two novel endophytic genera were proposed within the family. For instance, strain YNE01575 clustered with a previously misidentified strain (‘*Didymosphaeria*’ sp. ARM1124); these two strains shared 100% ITS sequence identity and together formed a distinct clade (D9, Figure 1), for which the new genus *Proxiconiothyrium* was proposed. Morphologically, this fungus is characterised by globose to subglobose pycnidia, ampulliform conidiogenous cells, and aseptate conidia. Nevertheless, our understanding of this lineage remains incomplete, as only two strains have so far been assigned to the proposed genus. Notably, ARM 1124 is represented in GenBank (accession no. PP277137) only by ITS sequence data and lacks both a morphological description and multilocus sequence information. Therefore, it is imperative to extend sampling and thoroughly characterise additional strains to accurately delineate phylogenetic relationships and better understand their morphological variation.

The genus *Paraconiothyrium* (*Didymosphaeriaceae*, *Pleosporales*) was introduced by Verkley et al. (2014) with *Paraconiothyrium estuarinum* designated as the type species. Species of *Paraconiothyrium* occur as pathogens, saprobes, or endophytes on a range of hosts in *Fabaceae*, *Lycopodiaceae*, *Magnoliaceae*, *Poaceae*, *Rosaceae*, and *Rubiaceae* (Boonmee et al. 2021; Pem et al. 2024). Ariyawansa et al. (2020) demonstrated the polyphyletic nature of *Paraconiothyrium* within *Didymosphaeriaceae* based on LSU and SSU sequences. Consequently, some species have now been transferred to related genera such as *Didymosphaeria*, *Paracamarosporium*, *Paraphaeosphaeria*, and *Pseudocamarosporium* (Pem et al. 2024). Phylogenetic analyses in this study revealed three misidentified strains: ‘*Paraconiothyrium hakeae*’ CBS142521 grouped with *Didymosphaeria* (Clade D7, Figure 1), while ‘*Paraconiothyrium*’ spp. DS860 and DS1329, and ‘*Neokalmusia deguarnae*’ BRIP 75884a clustered with NME00265 in a distinct clade (D16, Figure 1), representing an undescribed genus. Remarkably, all four currently recognised strains within this clade lack observable reproductive structures. Strain NME00265 only exhibited mycelial features despite extensive culturing attempts on different media under varying conditions. However, these strains share remarkable ecological consistency, being exclusively isolated from *Poaceae* roots across geographically disparate regions (China, Australia, and the United States) (Table S1). This suggests a potential obligate endophytic lifestyle that may constrain morphological expression. Such phylogenetic-ecological congruence finds precedent in the endophytic genus *Muscodor*, where the morphological characteristics are limited to sterile mycelium frequently intertwined into rope-like strands (Chen et al. 2019; Samarakoon et al. 2020). Although morphological paucity presents taxonomic challenges, the robust phylogenetic divergence (100% MLBP/1.00 BIPP), coupled with a conserved ecological niche, provides compelling evidence for the genus-level delimitation from *Neokalmusia* and related genera. Phylogenetic analyses also revealed that the new collections YNE00904 and YNE00913 formed a robust clade (D32, Figure 1) with two root endophytic strains previously misidentified as ‘*Leptosphaerulina chartarum’* (C2-227 and 72C) (Figure 1), prompting the proposal of the novel genus *Chlamydosphaeromyces* to accommodate them. Additionally, seven new strains from this study were assigned to two known *Paraconiothyrium* species: five strains (YNE00964–YNE00966, YNE00973) clustered with *Para*. *bishopiae*, and two (YNE00613, YNE01332) with *Para*. *zingiberacearum*.

*Periconia* was historically placed in *Massarinaceae* (Zhang et al. 2009, 2012; Hyde et al. 2013), but Tanaka et al. (2015) reclassified this genus and its relatives into the resurrected family *Periconiaceae*. Consistent clustering of *Noosia*, *Bambusistroma*, and *Flavomyces* with species of *Periconia* (Knapp et al. 2015, 2019; Tanaka et al. 2015; Crous et al. 2018; Hyde et al. 2018; Phookamsak et al. 2019; Tian et al. 2022) led to *Periconia* being considered polyphyletic. However, recent studies synonymised *Bambusistroma* and *Noosia* with *Periconia* based on morphology and multilocus phylogeny (Yang et al. 2022). *Flavomyces fueloephazae*, described as a root endophyte in grasses (Knapp et al. 2015, 2019), was also shown to cluster within the *Periconia* lineage in subsequent analyses (Hongsanan et al. 2020; Tian et al. 2022; Yang et al. 2022). Nevertheless, its taxonomic status remained unresolved due to a lack of detailed morphology and additional materials. In this study, multilocus phylogeny (SSU, LSU, ITS, *ACT*, *RPB2*, *TEF1*, and *TUB2*) revealed distinct, well-supported clades within *Periconia*, with *F*. *fueloephazae* forming a distinct well-supported clade. Although the reproductive structure remains undescribed, bead-like chlamydospores similar to those in *P*. *circinata* (Zhao et al. 2021) and *P*. *longibrachiatum* (this study) were observed as well. Thus, the synonymisation of *Flavomyces fueloephazae* under *Periconia*, as *P*. *fueloephazae*, is proposed, emphasising the monophyly of the genus.

*Periconia macrospinosa* is a dominant dark septate endophyte (DSE) in grasses (Su et al. 2010; Knapp et al. 2012, 2019; Mandyam et al. 2012; Jumpponen et al. 2017; Rudgers et al. 2022), known for enhancing plant resistance to abiotic stress and aiding nutrient assimilation in host plants (Gaber et al. 2020, 2023; Moghaddam et al. 2022). Comparative genomic analysis of *P*. *macrospinosa* and other DSEs provides valuable insights into its ecological role (Knapp et al. 2018). However, some studies note morphological variability in *P. macrospinosa*, especially in conidial size, despite high phylogenetic similarity (Lefebvre et al. 1949; Ellis 1968; Mandyam et al. 2010; Knapp et al. 2018; Fors et al. 2020). Clarifying its taxonomic status is crucial for ecological and evolutionary studies. Multilocus phylogenetic analyses showed that the *P*. *macrospinosa* s.l. split into three distinct lineages, suggesting potential new taxa. Morphological variations were also observed among these lineages. Given the limitations of phylogeny and morphology alone in species delimitation, split network analysis and PHI tests were employed on both individual gene sequences and concatenated multilocus datasets to examine the *P*. *macrospinosa* s.l. lineage. Split network analysis allowed for the detection of genetic recombination events and the assessment of sequence divergence among strains (Bruen et al. 2006; Huson and Bryant 2006; Laurence et al. 2014; Chethana et al. 2021). The results revealed three network branches, with no statistically significant recombination being detected within the lineage.

Lücking et al. (2020) proposed an integrative taxonomy framework, combining phylogenetic, phenotypic, and reproductive biological evidence for reliable fungal classification. This approach is particularly relevant since morphological species recognition is sometimes inconclusive, as individuals of a species may exhibit subtle or cryptic differences (Cronquist 1978; Chethana et al. 2021). Mandyam et al. (2010) described both macro- and micromorphology of *P*. *macrospinosa*, and the present study builds on this by suggesting that cryptic species may exist. It is proposed that the branch comprising YNE00586, YNE00436, YNE00454, YNE00463, YNE01612, and CBS 135663 ‒ closely matching the earliest description (Lefebvre et al. 1949) ‒ should be retained as *P*. *macrospinosa* s.s. The two other clades are designated as new species, *P*. *catenata* and *P*. *neomacrospinosa*. Morphologically, *P*. *macrospinosa* s.s. can be distinguished from *P*. *catenata* and *P*. *neomacrospinosa* by its larger conidia bearing longer spines, along with the capacity to produce green pigments in culture. The conidia of *P*. *catenata* and *P*. *neomacrospinosa* are similar, but the conidiogenous cells of *P*. *catenata* form directly on the swollen hyphae, whereas *P*. *neomacrospinosa* often has distinct conidiophores.

Within *P*. *neomacrospinosa*, morphological differences were observed among strains from different geographical regions (e.g., A113, A115, and A126 from São Paulo, Brazil, versus NME00180 and NME00221 from Inner Mongolia, China; ZJE01828 and ZJE01829 from Zhejiang, China). Such morphological and genetic variation is likely shaped by geographic isolation, a well-known driver of speciation (Mayr 1963). Therefore, an integrative approach, considering both molecular and morphological data, is critical for accurate species delimitation. Furthermore, following the recommendations of Haelewaters et al. (2018), a conservative approach is advocated when different delimitation methods yield inconsistent results, refraining from recognising taxa as new species unless they are well supported as distinct evolutionary lineages.

*Pleosporales* is predominant among endophytic fungi associated with *Poaceae* plants in diverse habitats (Liu et al. 2021; Rudgers et al. 2022). However, reports of new endophytic taxa within this order have remained sporadic (Knapp et al. 2015; Thambugala et al. 2017; Pintye and Knapp 2021; Romero-Jiménez et al. 2022). In summary, the discovery of new *Massarineae* endophytes in this study advances our knowledge of *Pleosporales* diversity, helps to redefine current taxonomic boundaries, and provides insights into the ecological roles and biogeographical distribution of these fungi.

## Supplementary Material

Supplementary_Materials_final_version_Clean.docx
