# Supplementary material for: Exploring the dominant endophytic pleosporalean fungi in Poaceae plants: taxonomic novelties within the suborder Massarineae
Source: Mycology. 2025 Dec 6;17(2):481–508. doi: 10.1080/21501203.2025.2569933 (PMC13267030; doi:10.1080/21501203.2025.2569933)
Supplement: Supplementary_Materials_final_version_Clean.docx [file TMYC_A_2569933_SM9125.docx]

**Supplementary Files**

**Supplementary Tables**

**Table S1.** Strains used in the phylogenetic analyses of *Didymosphaeriaceae* and their GenBank accession numbers.

| **Species** | **Strain** | **Locality** | **Host and substrate** | ***SSU*** | ***LSU*** | ***ITS*** | ***RPB2*** | ***TEF1*** | **Genome** |
| --- | --- | --- | --- | --- | --- | --- | --- | --- | --- |
| *Byssothecium circinans* (outgroup) | CBS 675.92 | ̶ | ̶ | GU205235 | GU205217 | OM337536 | Genome | Genome | GCA_010015675.1 |
| *Massarina eburnea* (outgroup) | CBS 473.64 | ̶ | ̶ | GU296170 | GU301840 | OM337528 | GU371732 | GU349040 | GCA_010093635.1 |
| *Agrorhizomyces patris* | BBT16 | Hungary: Martonvásár | Healthy roots of wheat | PP264972 | PP265004 | PP264950 | ̶ | PP273252 | ̶ |
| *Agrorhizomyces patris* | CBS 151043 = BBT01(T) | Hungary: Martonvásár | Healthy roots of wheat | PP264967 | NG_243992 | NR_197907 | ̶ | PP273247 | ̶ |
| *Alloconiothyrium aptrootii* | CBS 980.95 (T) | Papua New Guinea, Central Province | Soil | ̶ | JX496234 | JX496121 | ̶ | ̶ | ̶ |
| *Alloconiothyrium aptrootii* | CBS 981.95 | Papua New Guinea: Central Province | Soil | ̶ | JX496235 | JX496122 | ̶ | ̶ | ̶ |
| *Austropleospora keteleeriae* | MFLUCC 18-1551, KUMCC 18-0217 (T) | China: Yunnan | Decaying cone of *Keteleeria fortunei* (*Pinaceae*) | MK347910 | NG_070075 | NR_163349 | MK434909 | MK360045 | ̶ |
| *Austropleospora ochracea* | KUMCC 20-0020 (T) | China: Guizhou | Dead twigs of an undetermined host | NG_081371 | MT799860 | MT799859 | ̶ | MT872714 | ̶ |
| *Bimuria novae-zelandiae* | CBS 107.79 = AFTOL-ID 931 (T) | ̶ | ̶ | NG_061017 | NG_058623 | NR_159620 | Genome | Genome | Bimuria novae-zelandiae CBS 107.79 v1.0 |
| *Bimuria omanensis* | SQUCC 15280 (T) | Oman: Jebel Akhdar | Decaying leaves of unidentified plant |  | MT271820 | MT274326 | ̶ | MT279046 | ̶ |
| *Chromolaenicola chiangraiensis* | MFLUCC 17-1493 (T) | Thailand: Chiang Rai | Dead stems of *Chromolaena odorata* (*Asteraceae*) | MN325011 | NG_070944 | MN325017 | MN335655 | MN335650 | ̶ |
| *Chromolaenicola nanensis* | MFLUCC 17-1473 (T) | Thailand: Chiang Rai | Dead stems of *Chromolaena odorata* (*Asteraceae*) | MN325009 | NG_070942 | MN325015 | MN335653 | MN335648 | ̶ |
| ‘*Coniothyrium juniperi*’ | CBS 610.72 (T) | ̶ | ̶ | ̶ | MH872291 | MH860594 | ̶ | ̶ | ̶ |
| *Cylindroaseptospora leucaenae* | MFLUCC 17-2424, KUMCC 18-0226 (T) | Thailand: Chiang Rai | Decaying pod of *Leucaena* sp. (*Fabaceae*) | MK347856 | NG_066310 | NR_163333 | ̶ | MK360047 | ̶ |
| *Deniquelata barringtoniae* | MFLUCC 11-0422 (T) | Thailand: Chiang Rai | Leaf of *Barringtonia asiatica* (Lecythidaceae) | JX254656 | NG_042696 | NR_111779 | ̶ | ̶ | ̶ |

**Table S1.** Continued.

| **Species** | **Strain** | **Locality** | **Host and substrate** | ***SSU*** | ***LSU*** | ***ITS*** | ***RPB2*** | ***TEF1*** | **Genome** |
| --- | --- | --- | --- | --- | --- | --- | --- | --- | --- |
| *Deniquelata barringtoniae* | MFLUCC 16-0271 = KUMCC 16-0161 | Thailand: Prachuap Khiri Khan | *Pandanus* sp. (*Pandanaceae*) |  | MH260291 | MH275059 | MH412753 | MH412766 | ̶ |
| *Deniquelata quercina* | ABRIICC 10068 = 20SA (T) | Iran: Ilam | Branches of *Quercus brantii* (*Fagaceae*) | MH316155 | MH316157 | MH316153 | ̶ | ̶ | ̶ |
| *Dictyoarthrinium hydei* | SQUCC 13296 (T) | ̶ | ̶ | MW077161 | ̶ | MW077145 | ̶ | MW075771 | ̶ |
| *Dictyoarthrinium musae* | MFLUCC 20-0105 (T) | ̶ | ̶ | MT482326 | MT482320 | MT482323 | ̶ | ̶ | ̶ |
| *Didymocrea sadasivanii* | CBS 438.65 (T) | ̶ | ̶ | DQ384066 | DQ384103 | MH858658 | ̶ | ̶ | ̶ |
| *Didymosphaeria brasiliense* | CBS 100299 (T) | Brazil: Patrocinio-Minas Gerais | Fruit of *Coffea arabica* (*Rubiaceae*) | AY642523 | NG_070605 | NR_163552 | ̶ | ̶ | ̶ |
| *Didymosphaeria rubi-ulmifolii* | MFLUCC 14-0023 (T) | Italy: Forlì-Cesena | Dead branch of *Rubus ulmifolius* (*Rosaceae*) | NG_063557 | KJ436586 | ̶ | ̶ | ̶ | ̶ |
| *Didymosphaeria rubi-ulmifolii* | MFLUCC 14-0024 (T) | Italy: Forlì-Cesena | Dead branch of *Rubus ulmifolius* (*Rosaceae*) | KJ436587 | KJ436585 | ̶ | ̶ | ̶ | ̶ |
| *Didymosphaeria rubi-ulmifolii* | MFLUCC 16-1000 |  |  | MT226672 | MT214555 | MT310602 | ̶ | MT394734 | ̶ |
| *Didymosphaeria variabile* | CBS 121163 = STE-U 6311 (T) | South Africa: Western Cape | Discoloured wood in trunk of *Actinidia chinensis* (*Actinidiaceae*) | NG_064914 | ̶ | NR_137006 | ̶ | ̶ | ̶ |
| *Didymosphaeria variabile* | CBS 121754 = STE-U 6313 | South Africa: Western Cape | Small reddish brown V-shaped necrosis under cracked bark of *Prunus salicina* (*Rosaceae*) | ̶ | JX496144 | JX496031 | ̶ | ̶ | ̶ |
| *Didymosphaeria variabile* | CBS120014 = ER1386 | Italy: Latina | Discoloured wood in trunk of *Actinidia chinensis* (*Actinidiaceae*) | ̶ | JX496139 | JX496026 | ̶ | ̶ | ̶ |
| ‘*Paraconiothyrium hakeae*’ | CBS 142521, CPC 27651 | Australia: Western Australia | *Hakea* sp. (*Proteaceae*) | ̶ | KY979809 | NR_171729 | KY979847 | ̶ | ̶ |
| *Chlamydosphaeromyces* sp. (as *Leptosphaerulina* cf *chartarum*) | 72C | Brazil | Roots of *Combretum lanceolatum* (*Combretaceae*) | ̶ | ̶ | KF555232 | ̶ | ̶ | ̶ |
| *Chlamydosphaeromyces* sp. (as *Leptosphaerulina chartarum*) | C2-227 | Brazil | Roots of *Cyperus esculentus* (*Cyperaceae*) | ̶ | ̶ | KJ439067 | ̶ | ̶ | ̶ |
| *Chlamydosphaeromyces graminearum* | YNE00904 | China: Yunnan | Healthy roots of *Poaceae* plant | ̶ | NMDCN0007RIR | NMDCN0007RIL | NMDCN0007RJK | NMDCN0007RJ8 | ̶ |
| *Chlamydosphaeromyces graminearum* | YNE00913 | China: Yunnan | Healthy leaves of *Poaceae* plant | ̶ | NMDCN0007RIS | NMDCN0007RIM | NMDCN0007RJL | NMDCN0007RJ9 | ̶ |

**Table S1.** Continued.

| **Species** | **Strain** | **Locality** | **Host and substrate** | ***SSU*** | ***LSU*** | ***ITS*** | ***RPB2*** | ***TEF1*** | **Genome** |
| --- | --- | --- | --- | --- | --- | --- | --- | --- | --- |
| *Kalmusia ebuli* | CBS 123120 (T) | ̶ | ̶ | JN851818 | JN644073 | KF796674 | ̶ | ̶ | ̶ |
| *Kalmusia longispora* | CBS 824.84 (T) | Manitoba | *Arceuthobium pusillum* (*Santalaceae*) | ̶ | MH873526 | NR_153979 | ̶ | ̶ | ̶ |
| *Kalmusia variispora* | CBS 121517 | ̶ | ̶ | ̶ | MH874668 | MH863113 | ̶ | ̶ | ̶ |
| *Kalmusibambusa triseptata* | MFLUCC 13–0232, KUMCC 16–0183 (T) | Thailand: Chiang Rai | Living culms of bamboo (*Poaceae*) | KY682696 | KY682695 | KY682697 | ̶ | ̶ | ̶ |
| *Karstenula rhodostoma* | CBS 690.94 | Sweden | Dead stems | GU296154 | GU301821 | OM337539 | Genome | Genome | *Karstenula rhodostoma* CBS 690.94 v1.0 |
| *Karstenula rhodostoma* | CBS 691.94 | ̶ | ̶ | AB797241 | AB807531 |  | ̶ | AB808506 | ̶ |
| *Laburnicola centaureae* | MFLUCC 13-0601 (T) | Italy: Forlì-Cesena | Dead hanging branches of *Centaurea* sp. (*Asteraceae*) | KU743193 | KU743192 | KX274239 | ̶ | KU743212 | ̶ |
| *Laburnicola hawksworthii* | MFLUCC 13-0602 (T) | Italy: Forlì-Cesena | Dead stem of *Laburnum* sp. (*Fabaceae*) | KU743196 | KU743195 | KU743194 | ̶ | ̶ | ̶ |
| *Laburnicola muriformis* | MFLUCC 16-0290 (T) | Italy: Forlì-Cesena | Dead branch of *Laburnum anagyroides* (*Fabaceae*) | KU743199 | KU743198 | KU743197 | ̶ | KU743213 | ̶ |
| *Laburnicola radiciphila* | F6B1 = DSM112862 (T) | Hungary: Kiskunság | Root of *Festuca vaginata* (*Poaceae*) | ON876670 | ON870566 | NR_187029 | ̶ | ON892832 | ̶ |
| *Laburnicola radiciphila* | NME00334 | China: Inner Mongolia | Healthy roots of *Cleistogenes* sp. (*Poaceae*) | ̶ | NMDCN0007RIO | NMDCN0007RIJ | ̶ | NMDCN0007RJ5 | ̶ |
| *Laburnicola rhizohalophila* | CGMCC 8756= JP-R-44 (T) | China: Shandong | Surface-sterilized roots of *Suaeda salsa* (*Amaranthaceae*) | ̶ | KJ125523 | KJ125522 | KJ125524 | KJ125525 | ̶ |
| *Letendraea cordylinicola* | MFLUCC 11-0148 (T) | Thailand: Mae Jai | Dead leaves of *Cordyline* sp. (*Asparagaceae*) | NG_068362 | NG_059530 | NR_154118 | ̶ | ̶ | ̶ |
| *Letendraea eurotioides* | CBS 212.31 | ̶ | ̶ | ̶ | AY787935 | ̶ | ̶ | ̶ | ̶ |
| *Montagnula chromolaenicola* | MFLUCC 17-1469 (T) | Thailand: Mae Hong Son | Dead stems of *Chromolaena odorata* (*Asteraceae*) | NG_070157 | NG_070948 | NR_168866 | MT235809 | MT235773 | ̶ |
| *Montagnula chromolaenicola* | YNE01157 | China: Yunnan | Healthy stems of *Poaceae* plant | ̶ | NMDCN0007RJ0 | NMDCN0007RIK | NMDCN0007RJP | NMDCN0007RJD | ̶ |
| *Montagnula graminicola* | MFLUCC 13-0352 (T) | Italy: Forlì-Cesena | Dead stem of grass (*Poaceae*) | KM658316 | KM658315 | KM658314 | ̶ | ̶ | ̶ |
| *Montagnula saikhuensis* | MFLUCC 16-0315 (T) | Thailand: Prachuap Khiri Khan | Dead stem of *Citrus* sp. (*Rutaceae*) | KU743211 | KU743210 | KU743209 | ̶ | ̶ | ̶ |

**Table S1.** Continued.

| **Species** | **Strain** | **Locality** | **Host and substrate** | ***SSU*** | ***LSU*** | ***ITS*** | ***RPB2*** | ***TEF1*** | **Genome** |
| --- | --- | --- | --- | --- | --- | --- | --- | --- | --- |
| *Montagnula thailandica* | MFLUCC 17-1508 (T) | Thailand: Chiang Mai | Dead stems of *Chromolaena odorata* (*Asteraceae*) | NG_070158 | NG_070949 | MT214352 | MT235810 | MT235774 | ̶ |
| *Neokalmusia brevispora* | CBS 120248 = JCM 13543 = MAFF 239276 | Japan: Fukushima | Dead twigs of *Sasa* sp. (*Poaceae*) | ̶ | MH874633 | MH863078 | AB539099 | AB539112 | ̶ |
| *Neokalmusia brevispora* | NBRC 106240 (T) | Japan: Hokkaido | Dead twigs of *Sasa kurilensis* (*Poaceae*) | AB524460 | AB524601 | NR_154262 | AB539100 | AB539113 | ̶ |
| ‘*Paraconiothyrium* sp.’ | DS1329 | USA | Roots of *Bouteloua eriopoda* (*Poaceae*) | ̶ | ̶ | MK808285 | ̶ | ̶ | ̶ |
| ‘*Paraconiothyrium* sp.' | DS860 | USA | Roots of *Schizachyrium scoparium* (*Poaceae*) | ̶ | ̶ | MK808950 | ̶ | ̶ | ̶ |
| *Didymosphaeriaceae* sp. | NME00265 | China: Inner Mongolia | Healthy roots of *Agropyron* sp. (*Poaceae*) | ̶ | NMDCN0007RIN | NMDCN0007RI7 | NMDCN0007RJH | NMDCN0007RJ4 | ̶ |
| *'Neokalmusia deguarnae*’ | BRIP 75884a (T) | Australia: Queensland | *Panicum* sp. (*Poaceae*) | ̶ | PP707922 | NR_198784 | ̶ | ̶ | ̶ |
| *Neptunomyces aureus* | CMG 10A = MUM 19.38 (T) | Portugal: Ria de Aveiro | *Gracilaria gracilis* (*Gracilariaceae*) | ̶ | ̶ | MK912119 | ̶ | MK947998 | ̶ |
| *Neptunomyces juncicola* | CBS 150790 = CPC 45436 | Netherlands: North Holland | *Juncus maritimus* (Juncaceae) | ̶ | NG_244054 | NR_197932 | ̶ | PP780627 | ̶ |
| *Neokalmusia jonahhulmei* | KUMCC 21-0818 (T) | China: Yunnan |  | NG_148908 | ON007039 | NR_182587 | ON009137 | ON009133 | ̶ |
| *Neokalmusia kunmingensis* | KUMCC 18-0120 (T) | China: Yunnan | Dead bamboo culms (*Poaceae*) | MK079887 | MK079889 | MK079886 | ̶ | MK070172 | ̶ |
| *Neptunomyces litoralis* | BRIP 75555a | Australia: New South Wales | sand | ̶ | NG_242141 | NR_189984 | ̶ | ̶ | ̶ |
| *Neptunomyces* sp. | 80227 | ̶ | ̶ | ̶ | ̶ | OR760523 | ̶ | ̶ | ̶ |
| *Neptunomyces* sp. | LME4O1_7 | Costa Rica | *Coffea arabica* (*Rubiaceae*) | ̶ | ̶ | OR296983 | ̶ | ̶ | ̶ |
| *Neptunomyces* sp. | YNE01044 | China: Yunnan | Healthy stems of *Poaceae* plant | ̶ | NMDCN0007RIV | NMDCN0007RII | NMDCN0007RJO | NMDCN0007RJC | ̶ |
| *Neptunomyces yunnanensis* | SFC102397 | ̶ | ̶ | ̶ | ̶ | MH374562 | ̶ | ̶ | ̶ |
| *Neptunomyces yunnanensis* | zzz714 | China: Guangxi | Moso bamboo seeds (*Poaceae*) | ̶ | ̶ | HQ696074 | ̶ | ̶ | ̶ |
| *Neptunomyces yunnanensis* | YNE00523 | China: Yunnan | Healthy leaves of *Oryza meyeriana* subsp. *granulata* (*Poaceae*) | ̶ | NMDCN0007RIP | NMDCN0007RIH | NMDCN0007RJI | NMDCN0007RJ6 |  |

**Table S1.** Continued.

| **Species** | **Strain** | **Locality** | **Host and substrate** | ***SSU*** | ***LSU*** | ***ITS*** | ***RPB2*** | ***TEF1*** | **Genome** |
| --- | --- | --- | --- | --- | --- | --- | --- | --- | --- |
| *Proxiconiothyrium yunnanense* (as *Didymosphaeria* sp.) | ARM 1124 | Brazil: Araripina | ̶ | ̶ | ̶ | PP277137 | ̶ | ̶ | ̶ |
| *Proxiconiothyrium yunnanense* | YNE01575 | China: Yunnan | Healthy leaves of *Oryza meyeriana* subsp. *granulata* (*Poaceae*) | ̶ | NMDCN0007RJ2 | NMDCN0007RIF | NMDCN0007RJR | NMDCN0007RJF | ̶ |
| *Paracamarosporium fagi* | CPC 24890, CBS 140008 | ̶ | ̶ | ̶ | NG_070630 | NR_154318 | ̶ | ̶ | ̶ |
| *Paracamarosporium hawaiiense* | CBS 120025 | South Africa: Western Cape | Stems of *Psoralea pinnata* (*Fabaceae*) | EU295655 | JX496140 | JX496027 | ̶ | ̶ | ̶ |
| *Paracamarosporium psoraleae* | CPC 21632 =CBS 136628 (T) | South Africa: Western Cape | Stems of *Psoralea pinnata* (*Fabaceae*) | ̶ | KF777199 | KF777143 | ̶ | ̶ | ̶ |
| *Paraconiothyrium bishopiae* | BRIP 72437b |  |  | ̶ | OP598066 | NR_182624.1 | ̶ | ̶ | ̶ |
| *Paraconiothyrium bishopiae* | YNE00964 | China: Yunnan | Healthy roots of *Poaceae* plant | ̶ | NMDCN0007RIT | NMDCN0007RIA | NMDCN0007RJM | NMDCN0007RJA | ̶ |
| *Paraconiothyrium bishopiae* | YNE00965 | China: Yunnan | Healthy roots of *Poaceae* plant | ̶ | ̶ | NMDCN0007RIB | ̶ | ̶ | ̶ |
| *Paraconiothyrium bishopiae* | YNE00966 | China: Yunnan | Healthy roots of *Poaceae* plant | ̶ | ̶ | NMDCN0007RIC | ̶ | ̶ | ̶ |
| *Paraconiothyrium bishopiae* | YNE00973 | China: Yunnan | Healthy roots of *Poaceae* plant | ̶ | ̶ | NMDCN0007RID | ̶ | ̶ | ̶ |
| *Paraconiothyrium bishopiae* | YNE00974 | China: Yunnan | Healthy roots of *Poaceae* plant | ̶ | NMDCN0007RIU | NMDCN0007RIE | NMDCN0007RJN | NMDCN0007RJB | ̶ |
| *Paraconiothyrium cyclothyrioides* | CBS 972.95 | Papua New Guinea: Central Province | Soil | AY642524 | JX496232 | JX496119 | ̶ | ̶ | ̶ |
| *Paraconiothyrium estuarinum* | CBS 109850=CCT6596 | Brazil: São Paulo State | Estuarine sediment polluted with industrial discharges | AY642522 | MH874432 | MH862842 | LT854937 | ̶ | ̶ |
| *Paraconiothyrium iridis* | CPC 36281 = CBS 146036 (T) | Ukraine: Chernihiv region | Living leaves of *Iris pseudacorus* (*Iridaceae*) | ̶ | NG_074423 | NR_170059 | MT223695 | ̶ | ̶ |
| ‘*Paraconiothyrium marshiae*’ | BRIP 75785a | ̶ | ̶ | ̶ | PQ047740 | PQ061115 | ̶ | ̶ | ̶ |
| *Paraconiothyrium thysanolaenae* | MFLUCC 10-0550 (T) | Thailand: Chiang Mai | Dead stem of *Thysanolaena maxima* (*Poaceae*) | KP753959 | KP744496 | KP744453 | ̶ | ̶ | ̶ |
| *Paraconiothyrium zingiberacearum* | NCYUCC 19-0230 | ̶ | ̶ | ̶ | ON117304 | ON117286 | ̶ | ̶ | ̶ |
| *Paraconiothyrium zingiberacearum* | YNE00613 | China: Yunnan | Healthy stems of *Hyparrhenia* sp. (*Poaceae*) | ̶ | NMDCN0007RIQ | NMDCN0007RI8 | NMDCN0007RJJ | NMDCN0007RJ7 | ̶ |

**Table S1.** Continued.

| **Species** | **Strain** | **Locality** | **Host and substrate** | ***SSU*** | ***LSU*** | ***ITS*** | ***RPB2*** | ***TEF1*** | **Genome** |
| --- | --- | --- | --- | --- | --- | --- | --- | --- | --- |
| *Paraconiothyrium zingiberacearum* | YNE01332 | China: Yunnan | Healthy roots of *Poaceae* plant | ̶ | NMDCN0007RJ1 | NMDCN0007RI9 | NMDCN0007RJQ | NMDCN0007RJE | ̶ |
| *Paramassariosphaeria anthostomoides* | CBS 615.86 | ̶ | ̶ | GU205246 | MH873693 | MH862005 | ̶ | ̶ | ̶ |
| *Paramassariosphaeria clematidicola* | MFLUCC 16-0172 (T) | Italy: Forlì-Cesena | Dead stem of *Clematis vitalba* (*Ranunculaceae*) | KU743208 | KU743207 | KU743206 | ̶ | ̶ | ̶ |
| *Paraphaeosphaeria michotii* | CBS 340.86 | ̶ | Leaf of *Phragmites australis* (*Poaceae*) | ̶ | JX496192 | JX496079 | ̶ | ̶ | ̶ |
| *Paraphaeosphaeria michotii* | MAFF 243861 | Japan: Tochigi | Dead leaves of *Typha latifolia* (*Typhaceae*) | AB797269 | AB807559 | AB809639 | ̶ | AB808535 | ̶ |
| *Paraphaeosphaeria minitans* | CBS 859.71 | USA: California | Sclerotia of *Sclerotinia sclerotiorum* | ̶ | JX496229 | JX496116 | ̶ | ̶ | ̶ |
| *Paraphaeosphaeria neglecta* | CBS 124078 (T) | Italy: Latina | Wood of *Actinidia chinensis* (*Actinidiaceae*) | ̶ | JX496152 | JX496039 | ̶ | ̶ | ̶ |
| *Paraphaeosphaeria neglecta* | CBS 611.72 | ̶ | ̶ | ̶ | ̶ | MH860595 | ̶ | ̶ | ̶ |
| *Paraphaeosphaeria neglecta* | ZJE01696 | China: Zhejiang | Healthy roots of *Miscanthus* sp. (*Poaceae*) | ̶ | NMDCN0007RJ3 | NMDCN0007RIG | NMDCN0007RJS | NMDCN0007RJG | ̶ |
| *Paraphaeosphaeria sardoa* | CBS 501.71 (T) | Italy: Sardinia | Dead leaves of *Smilax aspera* (*Smilacaceae*) | ̶ | JX496207 | JX496094 | ̶ | ̶ | ̶ |
| *Phaeodothis mori* | MFLUCC 18-1634 | ̶ | ̶ | MN356452 | MN356450 | MN356454 | ̶ | MN364867 | ̶ |
| *Phaeodothis winteri* | CBS 182.58 | ̶ | ̶ | GU296183 | GU301857 |  | ̶ | DQ677917 | ̶ |
| *Pleoardoris graminearum* | DS1613 | USA: New Mexico | ̶ | MT707495 | MT707521 |  | ̶ | ̶ | ̶ |
| *Pleoardoris graminearum* | DS304 (T) | USA: Texas | Roots of *Bouteloua gracilis* (*Poaceae*) | MT707488 | MT707514 |  | ̶ | ̶ | ̶ |
| *Pleoardoris graminearum* | DS334 | USA: Texas | ̶ | MT707489 | MT707515 |  | ̶ | ̶ | ̶ |
| *Pseudocamarosporium corni* | MFLUCC 13-0541 = ICMP 20369 = GUCC 0010 (T) | Italy: Arezzo Province | Dead branch of *Cornus sanguinea* (*Cornaceae*) | KJ819946 | KJ813279 | KJ747048 | ̶ | ̶ | ̶ |
| *Pseudocamarosporium propinquum* | MFLUCC 13-0544 = ICMP 20371 = GUCC 0013 (T) | France: Rouen | Branch of *Salix vitellina* (*Salicaceae*) | KJ819949 | KJ813280 | KJ747049 | ̶ | ̶ | ̶ |
| *Pseudocamarosporium pteleae* | MFLUCC 17-0724 (T) | USA: California | Leaves of *Eucalyptus* sp. (*Myrtaceae*) | MG829166 | MG829061 | NR_157536 | ̶ | MG829233 | ̶ |
| *Pseudocamarosporium ulmi-minoris* | MFLUCC 17-0671 (T) | USA: California | Leaves of *Eucalyptus* sp. (*Myrtaceae*) | MG829167 | MG829062 | NR_157537 | ̶ | ̶ | ̶ |
| *Pseudodidymocyrtis lobariellae* | A.F.25130 | ̶ | ̶ | MT674676 | MT153989 | MT153960 | ̶ | ̶ | ̶ |

**Table S1.** Continued.

| **Species** | **Strain** | **Locality** | **Host and substrate** | ***SSU*** | ***LSU*** | ***ITS*** | ***RPB2*** | ***TEF1*** | **Genome** |
| --- | --- | --- | --- | --- | --- | --- | --- | --- | --- |
| *Pseudopithomyces chartarum* | MUCL 15905 | Belgium | Unknown substrate | ̶ | LK936383 | LK936375 | LK936439 | ̶ | ̶ |
| *Pseudopithomyces kunmingensis* | MFLUCC 17-0314 = KUMCC 16-0222 | China: Yunnan | Stems of unidentified grass | MF173606 | MF173605 | MF173607 | ̶ | ̶ | ̶ |
| *Pseudopithomyces mori* | MFLUCC 18-1630, KUMCC 19-0130 (T) | Taiwan: Chiayi | Dead leaves of *Morus australis* (*Moraceae*) | MW079343 | MW063214 | MW063153 | ̶ | MW183777 | ̶ |
| *Spegazzinia bromeliacearum* | URM 8084 (T) | Brazil: Pernambuco state | Endophyte from leaves of *Tilandsia catimbauensis* (*Bromeliaceae*) | ̶ | NG_242469 | NR_191084 | ̶ | ̶ | ̶ |
| *Spegazzinia radermacherae* | MFLUCC 17-2285, KUMCC 18-0297 (T) | Thailand: Chiang Rai | Fallen pod of *Radermachera sinica* (*Bignoniaceae*) | MK347848 | MK347957 | MK347740 | MK434893 | MK360088 | ̶ |
| *Spegazzinia tessarthra* | MAFF 243875 | Japan: Aomori | Balsa wood | ̶ | ̶ | LC757459 | ̶ | ̶ | ̶ |
| *Tremateia camporesii* | MFLU 19-2109 | China: Guizhou | Dead branch | MN473050 | MN473056 | MN473061 | ̶ | MN481602 | ̶ |
| *Tremateia chiangraiensis* | MFLUCC 17-1428 (T) | Thailand: Chiang Rai | Dead stems of *Chromolaena odorata* (*Asteraceae*) | NG_070159 | NG_068709 | NR_168867 | MT235813 | MT235775 | ̶ |
| *Verrucoconiothyrium nitidae* | CBS 119209, CMW 19988 | South Africa | Dead twigs of *Leucadendron salignum* (*Proteaceae*) | ̶ | EU552112 | EU552112 | ̶ | ̶ | ̶ |
| *Vicosamyces venturisporus* | CDA1494 | Brazil: Minas Gerais | Leaves of *Eugenia* sp. (*Myrtaceae*) | ̶ | MF802828 | MF802825 | ̶ | ̶ | ̶ |
| *Vicosamyces venturisporus* | CDA1495 | Brazil: Minas Gerais | Leaves of *Eugenia* sp. (*Myrtaceae*) | ̶ | MF802829 | MF802826 | ̶ | ̶ | ̶ |
| *Xenocamarosporium acaciae* | CPC 24755 = CBS 139895 (T) | Malaysia: Sabah | Leaf spots of *Acacia mangium* (*Leguminosae*) | ̶ | NG_058163 | NR_137982 | ̶ | ̶ | ̶ |
| *Xenocamarosporium acaciae* | MFLUCC 17-2432 | ̶ | ̶ | MK347873 | MK347983 | MK347766 | ̶ | MK360093 | ̶ |
| *Xenocamarosporium acaciae* | ZHKUCC 22-0203 | ̶ | ̶ | OP297790 | OP297776 | OP297806 | ̶ | OP321575 | ̶ |

Notes: (1) Newly generated sequences are indicated in red. (2) For genomic loci, accession numbers beginning with “NMDCXXXXXXXX” are deposited in the National Microbiology Data Center (NMDC), while all other accession numbers are deposited in National Center for Biotechnology Information (NCBI) GenBank. (3) For whole genome, accession numbers beginning with “GCA_XXXXXXXXX.X” are deposited in NCBI GenBank; all other genome accession numbers are deposited in the JGI Genome Database.

**Table S2.** Strains used in the phylogenetic analyses of *Periconiaceae* and their GenBank accession numbers.

| **Species** | **Specimen/culture** | **Locality** | **Host and substrate** | **SSU** | **LSU** | **ITS** | ***ACT*** | ***RPB2*** | ***TEF1*** | ***TUB2*** | **Genome** |
| --- | --- | --- | --- | --- | --- | --- | --- | --- | --- | --- | --- |
| *Byssothecium circinans* (outgroup) | CBS 675.92 | ̶ | ̶ | GU205235 | GU205217 | OM337536 | Genome | Genome | GU349061 | Genome | GCA_010015675.1 |
| *Helminthosporium solani* (outgroup) | CBS 640.85 | ̶ | ̶ | Genome | Genome | Genome | Genome | Genome | Genome | Genome | GCA_022560085.1 |
| *Periconia algeriana* | CBS 321.79 | Algeria | ̶ | ̶ | MH872979 | MH861212 | ̶ | ̶ | ̶ | ̶ | ̶ |
| *Periconia alishanica* | KUMCC 19-0174 | China: Taiwan | Dead leaves of Morus australis (Moraceae) | ̶ | MW063231 | MW063167 | ̶ | ̶ | MW183792 | ̶ | ̶ |
| *Periconia alishanica* | MFLUCC 19-0145 (T) | China: Taiwan | Dead leaves of Ficus septica (Moraceae) | ̶ | MW063229 | MW063165 | ̶ | ̶ | MW183790 | ̶ | ̶ |
| *Periconia alishanica* | NCYUCC 19-0186 | China: Taiwan | Dead leaves of Macaranga tanarius (Euphorbiaceae) | ̶ | MW063230 | MW063166 | ̶ | ̶ | MW183791 | ̶ | ̶ |
| *Periconia ananasi* | KUMCC 21-0470 | Thailand: Chiang Rai | Dead leaves of Ananas comosus (Bromeliaceae) | OL979226 | OL985955 | OM102539 | ̶ | ̶ | OM007977 | ̶ | ̶ |
| *Periconia ananasi* | MFLUCC 21-0155 (T) | Thailand: Chiang Rai | Dead leaves of Ananas comosus (Bromeliaceae) | OL606142 | NG_243062 | NR_190247 | ̶ | ̶ | OL912946 | ̶ | ̶ |
| *Periconia aquatica* | MFLUCC 16-0912 (T) | China: Yunnan | Submerged wood | ̶ | KY794705 | NR_158841 | ̶ | ̶ | KY814760 | ̶ | ̶ |
| *Periconia artemisiae* | C75 | China: Yunnan | Canopy air | ̶ | ̶ | MK304380 | ̶ | ̶ | ̶ | ̶ | ̶ |
| *Periconia artemisiae* | G1782 | ̶ | ̶ | ̶ | ̶ | MK247789 | ̶ | ̶ | ̶ | ̶ | ̶ |
| *Periconia artemisiae* | KUMCC 20-0265 (T) | China: Yunnan | Dead branches of Artemisia caruifolia (Asteraceae) | ̶ | MW448571 | MW448657 | ̶ | ̶ | MW460898 | ̶ | ̶ |
| *Periconia atropurpurea* | CBS 381.55 | Mozambique | ̶ | ̶ | MH869061 | MH857524 | ̶ | ̶ | ̶ | ̶ | ̶ |

**Table S2.** Continued.

| **Species** | **Specimen/culture** | **Locality** | **Host and substrate** | **SSU** | **LSU** | **ITS** | ***ACT*** | ***RPB2*** | ***TEF1*** | ***TUB2*** | **Genome** |
| --- | --- | --- | --- | --- | --- | --- | --- | --- | --- | --- | --- |
| *Periconia banksiae* | CBS 1295265 (T) | Australia: Queensland | Leaves of Banksia aemula (Proteaceae) | ̶ | NG_064279 | NR_190907 | ̶ | ̶ | ̶ | ̶ | ̶ |
| *Periconia byssoides* | MFLUCC 17-2292 | Thailand: Ko Larn island | Decaying pod of Peltophorum sp. (Fabaceae) | MK347858 | MK347968 | MK347751 | ̶ | MK434886 | MK360069 | ̶ | ̶ |
| *Periconia byssoides* | MFLUCC 18-1553 | China: Yunnan | Decaying cone of Magnolia grandiflora (Magnoliaceae) | MK347914 | MK348025 | MK347806 | ̶ | MK434858 | MK360068 | ̶ | ̶ |
| *Periconia byssoides* | YNE00527 | China: Yunnan | Leaves of Oryza meyeriana subsp. granulata (Poaceae) | ̶ | PP106220 | PP106188 | PP112496 | PP112524 | PP112549 | PP112574 | ̶ |
| *Periconia byssoides* | YNE00806 | China: Yunnan | Leaves of Pseudechinolaena sp. (Poaceae) | ̶ | PP106224 | PP106189 | PP112497 | PP112525 | PP112548 | PP112575 | ̶ |
| *Periconia caespitosa* | LAMIC 110/165 (T) | Brazil: Ceará | Decaying leaves of dicotyledonous plant | ̶ | MH051907 | MH051906 | ̶ | ̶ | ̶ | ̶ | ̶ |
| *Periconia calamagrostidicola* | CBS 150887 | Netherlands: Utrecht | Old leaves of Calamagrostis arenaria (Poaceae) | ̶ | NG_244051 | NR_197928 | ̶ | ̶ | ̶ | ̶ | ̶ |
| *Periconia catenata* | YNE00796 | China: Yunnan | Roots of Capillipedium sp. (Poaceae) | ̶ | PP106223 | PP106200 | ̶ | PP112510 | PP112538 | PP112563 | ̶ |
| *Periconia catenata* | GDMCC 3.10415 (T) | China: Yunnan | Roots of Capillipedium sp. (Poaceae) | ̶ | PP106225 | PP106201 | PP112483 | PP112512 | PP112539 | PP112564 | ̶ |
| *Periconia catenata* | YNE01006 | China: Yunnan | Roots of Poaceae sp. | ̶ | PP106226 | PP106202 | PP112484 | PP112511 | PP112540 | PP112565 | ̶ |
| *Periconia celtidis* | MFLUCC 20-01725 (T) | China: Taiwan | Dead leaves of Celtis tetrandra (Cannabaceae) | ̶ | NG_079543 | NR_174830 | ̶ | ̶ | ̶ | ̶ | ̶ |

**Table S2.** Continued.

| **Species** | **Specimen/culture** | **Locality** | **Host and substrate** | **SSU** | **LSU** | **ITS** | ***ACT*** | ***RPB2*** | ***TEF1*** | ***TUB2*** | **Genome** |
| --- | --- | --- | --- | --- | --- | --- | --- | --- | --- | --- | --- |
| *Periconia celtidis* | NCYUCC 19-0314 | China: Taiwan | Dead leaves of Macaranga tanarius (Euphorbiaceae) | ̶ | MW063227 | MW063163 | ̶ | ̶ | ̶ | ̶ | ̶ |
| *Periconia chengduensis* | CGMCC 3.239305 (T) | China: Sichuan | Dead culms of Pennisetum purpureum (Poaceae) | NG_242918 | NG_229079 | NR_185802 | ̶ | OP961469 | OP961453 | ̶ | ̶ |
| *Periconia chengduensis* | UESTCC 22.0140 | China: Sichuan | Dead culms of Miscanthus sinensis (Poaceae) | OP956046 | OP956002 | OP955977 | ̶ | OP961465 | OP961443 | ̶ | ̶ |
| *Periconia chengduensis* | UESTCC 22.0141 | China: Sichuan | Dead culms of Imperata cylindrica (Poaceae) | OP956041 | OP955997 | OP955972 | ̶ | ̶ | OP961438 | ̶ | ̶ |
| *Periconia chengduensis* | UESTCC 22.0142 | China: Sichuan | Dead culms of Phragmites australis (Poaceae) | OP956047 | OP956003 | OP955978 | ̶ | ̶ | OP961444 | ̶ | ̶ |
| *Periconia chengduensis* | UESTCC 22.0143 | China: Sichuan | Dead culms of Neyraudia reynaudiana (Poaceae) | OP956050 | OP956006 | OP955981 | ̶ | OP961466 | OP961447 | ̶ | ̶ |
| *Periconia chiangraiensis* | KUMCC 21-04715 (T) | Thailand: Chiang Rai | Dead leaves of Ananas comosus (Bromeliaceae) | OL979227 | OL985956 | OM102540 | ̶ | ̶ | OM007978 | ̶ | ̶ |
| *Periconia chiangraiensis* | MFLUCC 21-01645 (T) | Thailand: Chiang Rai | Dead leaves of Ananas comosus (Bromeliaceae) | OL606143 | OL606154 | OL753686 | ̶ | ̶ | OL912947 | ̶ | ̶ |
| *Periconia chimonanthi* | KUMCC 20-02665 (T) | China: Yunnan | Dead branches of Chimonanthi praecox (Calycanthaceae) | NG_081406 | NG_081512 | NR_176752 | ̶ | ̶ | MW460897 | ̶ | ̶ |

**Table S2.** Continued.

| **Species** | **Specimen/culture** | **Locality** | **Host and substrate** | **SSU** | **LSU** | **ITS** | ***ACT*** | ***RPB2*** | ***TEF1*** | ***TUB2*** | **Genome** |
| --- | --- | --- | --- | --- | --- | --- | --- | --- | --- | --- | --- |
| *Periconia chimonanthi* | UESTCC 22.0133 | China: Sichuan | Dead leaves of Arundo donax (Poaceae) | OP956033 | OP955989 | OP955964 | ̶ | OP961455 | OP961430 | ̶ | ̶ |
| *Periconia circinata* | CBS 263.37 | UK: Rothamsted | Soil | ̶ | MH867413 | MW810265 | ̶ | ̶ | MW735660 | MZ073941 | ̶ |
| *Periconia circinata* | CBS 414.50 | Portugal | Cereals (Poaceae) | ̶ | MH868210 | MH856694 | ̶ | ̶ | ̶ | MZ073904 | ̶ |
| *Periconia citlaltepetlensis* | IOM 325319.15 (T) | Mexico: Puebla | High altitude volcanic glacier | ̶ | MT625978 | MH890645 | ̶ | ̶ | ̶ | ̶ | ̶ |
| *Periconia citlaltepetlensis* | IOM 325319.25 (T) | Mexico: Puebla | High altitude volcanic glacier | ̶ | MT649216 | MT649221 | ̶ | ̶ | ̶ | ̶ | ̶ |
| *Periconia cookei* | GZCC21-0203 | China: Guizhou | Decaying inforescence of Trachycarpus fortunei (Arecaceae) | PP639223 | PP621091 | PP592463 | ̶ | PP780259 | PP828796 | ̶ | ̶ |
| *Periconia cookei* | MFLUCC 17-1399 | China: Yunnan | Submerged wood | ̶ | MG333493 | MN535816 | ̶ | ̶ | MG438279 | ̶ | ̶ |
| *Periconia cortaderiae* | MFLUCC 15-0451 | Thailand: Chiang Rai | Dead stems and leaves of Cortaderia sp. (Poaceae) | KX986346 | KX954403 | KX965734 | ̶ | ̶ | KY429208 | ̶ | ̶ |
| *Periconia cortaderiae* | MFLUCC 15-0453 | Thailand: Chiang Rai | Dead stems and leaves of Cortaderia sp. (Poaceae) | ̶ | KX954402 | KX965733 | ̶ | ̶ | KY320574 | ̶ | ̶ |
| *Periconia cortaderiae* | MFLUCC 15-04575 (T) | Thailand: Chiang Rai | Dead stems and leaves of Cortaderia sp. (Poaceae) | NG_068373 | NG_068238 | NR_165853 | ̶ | ̶ | KY310703 | ̶ | ̶ |
| *Periconia cortaderiae* | MFLUCC 20-0236 | Thailand: Nan | Dead leaves of Musa sp. (Musaceae) | MW406969 | MW406971 | MW406973 | ̶ | ̶ | MW422156 | ̶ | ̶ |
| *Periconia cynodontis* | CGMCC 3.239275 (T) | China: Sichuan | Dead leaves of Cynodon dactylon (Poaceae) | NG_242912 | NG_229070 | NR_185795 | ̶ | OP961460 | OP961434 | ̶ | ̶ |

**Table S2.** Continued.

| **Species** | **Specimen/culture** | **Locality** | **Host and substrate** | **SSU** | **LSU** | **ITS** | ***ACT*** | ***RPB2*** | ***TEF1*** | ***TUB2*** | **Genome** |
| --- | --- | --- | --- | --- | --- | --- | --- | --- | --- | --- | --- |
| *Periconia cynodontis* | YNE00594 | China: Yunnan | Leaf sheaths of Oplismenus sp. (Poaceae) | ̶ | PP106222 | PP106183 | PP112490 | PP112518 | PP112546 | PP112571 | ̶ |
| *Periconia cynodontis* | YNE01142 | China: Yunnan | Stems of Poaceae sp. | ̶ | PP106227 | PP106184 | PP112491 | PP112519 | PP112547 | PP112572 | ̶ |
| *Periconia cyperacearum* | CBS 1444345 (T) | Australia: New South Wales | Leaves of Cyperaceae sp. | ̶ | NG_064549 | NR_160357 | ̶ | ̶ | ̶ | ̶ | ̶ |
| *Periconia cyperacearum* | S219 | China: Guizhou | *Metagentiana rhodantha* (Gentianaceae) | ̶ | ̶ | MT576436 | ̶ | ̶ | ̶ | ̶ | ̶ |
| *Periconia cyperacearum* | S308 | China: Guizhou | *Metagentiana rhodantha* (Gentianaceae) | ̶ | ̶ | MT576437 | ̶ | ̶ | ̶ | ̶ | ̶ |
| *Periconia cyperacearum* | S321 | China: Guizhou | *Metagentiana rhodantha* (Gentianaceae) | ̶ | ̶ | MT576438 | ̶ | ̶ | ̶ | ̶ | ̶ |
| *Periconia delonicis* | MFLU 23-0180 | Thailand: Chiang Rai | Dead leaves of Cocos nucifera (Arecaceae) | OR458345 | OR438826 | OR438355 | ̶ | ̶ | OR500317 | ̶ | ̶ |
| *Periconia delonicis* | MFLUCC 17-25845 (T) | Thailand: Chiang Rai | Decaying pod of Delonix regia (Fabaceae) | NG_065770 | NG_068611 | ̶ | ̶ | MK434901 | MK360071 | ̶ | ̶ |
| *Periconia delonicis* | MFLUCC 20-0235 | Thailand: Chiang Rai | Dead leaf of Musa sp. (Musaceae) | ̶ | MW406970 | MW406972 | ̶ | ̶ | MW422155 | ̶ | ̶ |
| *Periconia delonicis* (as Periconia arecacearum) | MFLUCC 16-13875 (T) | Thailand: Chiang Rai | Fallen decaying Leaves of Cocos nucifera (Arecaceae) | PP639222 | PP621090 | PP592462 | ̶ | ̶ | PP828795 | ̶ | ̶ |
| *Periconia dicranopteridis* | HKAS 1299165 (T) | China: Guizhou | Roots of Dicranopteris pedata | PV138560 | PV138587 | PV138731 | ̶ | ̶ | PV177119 | ̶ | ̶ |

**Table S2.** Continued.

| **Species** | **Specimen/culture** | **Locality** | **Host and substrate** | **SSU** | **LSU** | **ITS** | ***ACT*** | ***RPB2*** | ***TEF1*** | ***TUB2*** | **Genome** |
| --- | --- | --- | --- | --- | --- | --- | --- | --- | --- | --- | --- |
| *Periconia dicranopteridis* | 4893 | Papua New Guinea | Ficus pungens | ̶ | ̶ | KR015891 | ̶ | ̶ | ̶ | ̶ | ̶ |
| *Periconia didymosporum* | MFLU 15-0058 | Thailand: Chiang Rai | Decaying culms of bamboo (Poaceae) | KP761738 | KP761731 | KP761734 | ̶ | KP761721 | KP761728 | ̶ | ̶ |
| *Periconia didymosporum* | MFLUCC 13-08625 (T) | Thailand: Chiang Rai | Decaying culms of bamboo (Poaceae) | NG_081379 | NG_081448 | NR_176693 | ̶ | KP761720 | KP761727 | ̶ | ̶ |
| *Periconia digitata* | CBS 510.77 | Kuwait | Salt-marsh mud | AB797271 | AB807561 | LC014584 | ̶ | ̶ | AB808537 | ̶ | ̶ |
| *Periconia digitata* | CNCM I-4278 | ̶ | ̶ | Genome | Genome | Genome | Genome | Genome | Genome | Genome | GCA_948474695.1 |
| *Periconia dujuanhuensis* | KUNCC 23-134825 (T) | China: Yunnan | Submerged decaying wood | ̶ | PP189907 | PQ340469 | ̶ | ̶ | PQ456957 | ̶ | ̶ |
| *Periconia elaeidis* | MFLUCC 17-00875 (T) | Thailand: Phrae | Decaying Elaeis guineensis (Arecaceae) | MH108551 | MH108552 | MG742713 | ̶ | ̶ | ̶ | ̶ | ̶ |
| *Periconia endophytica* | ZHKUCC 23-09955 (T) | China: Guangdong | Healthy leaves of Wurfbainia villosa (Zingiberaceae) | NG_243017 | NG_243969 | OR995582 | ̶ | ̶ | PP025968 | ̶ | ̶ |
| *Periconia endophytica* | ZHKUCC 23-0996 | China: Guangdong | Healthy leaves of Wurfbainia villosa (Zingiberaceae) | PP277723 | OR995589 | OR995583 | ̶ | ̶ | PP025969 | ̶ | ̶ |
| *Periconia epilithographicola* | CBS 1440175 (T) | Costa Rica | 19th century art lamina | ̶ | ̶ | NR_157477 | MF422179 | ̶ | ̶ | ̶ | ̶ |
| *Periconia epilithographicola* | MFLUCC 21-0153 | Thailand: Chiang Rai | Dead leaves of Ananas comosus (Bromeliaceae) | OL606144 | OL606155 | OL753687 | ̶ | ̶ | OL912948 | ̶ | ̶ |
| *Periconia festucae* | CGMCC 3.239295 (T) | China: Sichuan | Dead culms of Poaceae sp. | NG_242916 | NG_229077 | NR_185800 | ̶ | OP961463 | OP961439 | ̶ | ̶ |
| *Periconia festucae* | KNY31 | Kenya | Root of tree tomato | ̶ | ̶ | OQ953761 | ̶ | ̶ | ̶ | ̶ | ̶ |

**Table S2.** Continued.

| **Species** | **Specimen/culture** | **Locality** | **Host and substrate** | **SSU** | **LSU** | **ITS** | ***ACT*** | ***RPB2*** | ***TEF1*** | ***TUB2*** | **Genome** |
| --- | --- | --- | --- | --- | --- | --- | --- | --- | --- | --- | --- |
| *Periconia festucae* | R45 | Kenya | Barber shops in informal settlements | ̶ | ̶ | MT420640 | ̶ | ̶ | ̶ | ̶ | ̶ |
| *Periconia floridana* | CBS 1508845 (T) | USA: Florida | Outside air | ̶ | NG_244042 | NR_197917 | ̶ | ̶ | ̶ | ̶ | ̶ |
| *Periconia floridana* | CSB_F100 | Kenya | Brachiaria sp. | ̶ | ̶ | KU574713 | ̶ | ̶ | ̶ | ̶ | ̶ |
| *Periconia fueloephazae* | CBS 135664 | Hungary: Fülöpháza | Roots of Festuca vaginata (Poaceae) | KP184081 | KP184039 | KP184000 | KP184116 | ̶ | ̶ | ̶ | ̶ |
| *Periconia fueloephazae* | CBS 1357615 (T) | Hungary: Fülöpháza | Roots of Festuca vaginata (Poaceae) | NG_061191 | NG_058131 | NR_137960 | KP184118 | ̶ | ̶ | ̶ | ̶ |
| *Periconia fueloephazae* | NME00303 | China: Inner Mongolia | Roots of Cleistogenes squarrosa (Poaceae) | ̶ | PP106213 | PP106185 | PP112473 | PP112498 | PP112526 | PP112579 | ̶ |
| *Periconia fueloephazae* | NME00313 | China: Inner Mongolia | Roots of Cleistogenes squarrosa (Poaceae) | ̶ | PP106214 | PP106186 | PP112471 | PP112499 | PP112527 | PP112580 | ̶ |
| *Periconia fueloephazae* | NME00323 | China: Inner Mongolia | Roots of Cleistogenes squarrosa (Poaceae) | ̶ | PP106215 | PP106187 | PP112472 | PP112500 | PP112528 | PP112581 | ̶ |
| *Periconia genistae* | CBS 322.79 | Algeria | ̶ | ̶ | MH872980 | MH861213 | ̶ | ̶ | ̶ | ̶ | ̶ |
| *Periconia homothallica* | CBS 1396985 (T) | Japan: Yamagata | Dead leaves of Phragmites japonica (Poaceae) | NG_064851 | NG_059397 | NR_153446 | ̶ | ̶ | AB808541 | ̶ | ̶ |
| *Periconia homothallica* | LSU0157 | USA: Maryland | Diseased leaves of Phragmites australis (Poaceae) | MT000325 | MT000486 | MT000387 | ̶ | ̶ | ̶ | ̶ | ̶ |
| *Periconia hongheensis* | KUNCC 23-13549 | China: Yunnan | Submerged decaying wood | ̶ | PP189904 | PQ340466 | ̶ | ̶ | ̶ | ̶ | ̶ |
| *Periconia hongheensis* | KUNCC 23-135505 (T) | China: Yunnan | Submerged decaying wood | ̶ | PP189905 | PQ340467 | ̶ | ̶ | PQ456958 | ̶ | ̶ |
| *Periconia hydei* | ZHKUCC 24-21015 (T) | China: Yunnan | Submerged decaying twigs of Stipa capillata | PV437586 | PV436640 | PV434171 | ̶ | ̶ | PV588047 | ̶ | ̶ |

**Table S2.** Continued.

| **Species** | **Specimen/culture** | **Locality** | **Host and substrate** | **SSU** | **LSU** | **ITS** | ***ACT*** | ***RPB2*** | ***TEF1*** | ***TUB2*** | **Genome** |
| --- | --- | --- | --- | --- | --- | --- | --- | --- | --- | --- | --- |
| *Periconia hydei* | ZHKUCC 24-2102 | China: Yunnan | Submerged decaying twigs of Stipa capillata | PV437587 | PV436641 | PV434172 | ̶ | ̶ | PV588048 | ̶ | ̶ |
| *Periconia igniaria* | CBS 298.66 | South Africa | - | ̶ | MH870438 | MH858798 | ̶ | ̶ | ̶ | ̶ | ̶ |
| *Periconia igniaria* | CBS 583.66 | South Africa | - | ̶ | MH870553 | MH858888 | ̶ | ̶ | ̶ | ̶ | ̶ |
| *Periconia igniaria* | CBS 845.96 | Papua New Guinea | Bamboo (Poaceae) | AB797277 | AB807567 | LC014586 | ̶ | GU371793 | AB808543 | ̶ | ̶ |
| *Periconia imperatae* | CGMCC 3.239315 (T) | China: Sichuan | Dead leaves and culms of Imperata cylindrica (Poaceae) | NG_242917 | NG_229078 | NR_185801 | ̶ | OP961467 | OP961450 | ̶ | ̶ |
| *Periconia imperatae* | UESTCC 22.0145 | China: Sichuan | Dead leaves and culms of Imperata cylindrica (Poaceae) | OP956048 | OP956004 | OP955979 | ̶ | ̶ | OP961445 | ̶ | ̶ |
| *Periconia imperatae* | UESTCC 22.0146 | China: Sichuan | Dead culms of Neyraudia reynaudiana (Poaceae) | OP956052 | OP956008 | OP955983 | ̶ | ̶ | OP961449 | ̶ | ̶ |
| *Periconia imperatae* | YNE00457 | China: Yunnan | Roots of Eleusine indica (Poaceae) | ̶ | PP106218 | PP106190 | PP112492 | PP112523 | PP112550 | PP112573 | ̶ |
| *Periconia kunmingensis* | MFLUCC 18-06795 (T) | China: Yunnan | Dead rachis of fern | ̶ | MH892399 | MH892346 | ̶ | OR547996 | MH908963 | ̶ | ̶ |
| *Periconia lateralis* | CBS 292.36 | USA | - | ̶ | MH867311 | MH855804 | ̶ | ̶ | ̶ | ̶ | ̶ |
| *Periconia linzhiensis* | HKAS 144527 | China: Tibet | Bamboo | PQ675369 | PQ675409 | PQ684990 | ̶ | ̶ | PQ671466 | ̶ | ̶ |
| *Periconia linzhiensis* | KUNCC25-191235 (T) | China: Tibet | Bamboo | PQ675368 | PQ675408 | PQ684989 | ̶ | ̶ | PQ671465 | ̶ | ̶ |
| *Periconia longibrachiatum* | YNE01202 | China: Yunnan | Roots of Poaceae sp. | ̶ | PP106228 | PP106203 | PP112485 | PP112513 | PP112541 | PP112566 | ̶ |
| *Periconia longibrachiatum* | YNE01204 | China: Yunnan | Roots of Poaceae sp. | ̶ | PP106229 | PP106204 | PP112487 | PP112514 | PP112542 | PP112567 | ̶ |

**Table S2.** Continued.

| **Species** | **Specimen/culture** | **Locality** | **Host and substrate** | **SSU** | **LSU** | **ITS** | ***ACT*** | ***RPB2*** | ***TEF1*** | ***TUB2*** | **Genome** |
| --- | --- | --- | --- | --- | --- | --- | --- | --- | --- | --- | --- |
| *Periconia longibrachiatum* | GDMCC 3.10435 (T) | China: Yunnan | Roots of Poaceae sp. | ̶ | PP106231 | PP106205 | PP112486 | PP112515 | PP112543 | PP112568 | ̶ |
| *Periconia macrospinosa* | CBS 135663 | Hungary: Fülöpháza | Roots of Festuca vaginata (Poaceae) | KP184080 | KP184038 | KP183999 | KP184117 | ̶ | ̶ | ̶ | ̶ |
| *Periconia macrospinosa* | YNE00436 | China: Yunnan | Roots of Oryza meyeriana subsp. granulata (Poaceae) | ̶ | PP106216 | PP106193 | PP112478 | PP112505 | PP112533 | PP112558 | ̶ |
| *Periconia macrospinosa* | YNE00454 | China: Yunnan | Roots of Oryza meyeriana subsp. granulata (Poaceae) | ̶ | PP106217 | PP106194 | PP112481 | PP112508 | PP112537 | PP112559 | ̶ |
| *Periconia macrospinosa* | YNE00463 | China: Yunnan | Roots of Oplismenus sp. (*Poaceae*) | ̶ | PP106219 | PP106195 | PP112480 | PP112507 | PP112534 | PP112560 | ̶ |
| *Periconia macrospinosa* | YNE00586 | China: Yunnan | Roots of Oryza meyeriana subsp. granulata (Poaceae) | ̶ | PP106221 | PP106196 | PP112482 | PP112509 | PP112535 | PP112561 | ̶ |
| *Periconia macrospinosa* | YNE01612 | China: Yunnan | Roots of Poaceae sp. | ̶ | PP106234 | PP106197 | PP112479 | PP112506 | PP112536 | PP112562 | ̶ |
| *Periconia minutissima* | MFLUCC 15-0245 | China: Yunnan | Submerged wood | ̶ | KY794707 | KY794703 | ̶ | ̶ | ̶ | ̶ | ̶ |
| *Periconia motuoensis* | KUNCC24-179245 (T) | China: Tibet | Dead sheaths of Poaceae | PQ438116 | PQ438054 | PQ373177 | ̶ | ̶ | PQ661220 | ̶ | ̶ |
| *Periconia motuoensis* | KUNCC24-17925 | China: Tibet | Dead sheaths of Poaceae | PQ438117 | PQ438055 | PQ373178 | ̶ | ̶ | PQ661221 | ̶ | ̶ |
| *Periconia muchuanensis* | CGMCC 3.255995 (T) | China: Sichuan | Dead branches of bamboo (Poaceae) | PQ066546 | PQ067698 | PQ067783 | ̶ | ̶ | PQ278553 | ̶ | ̶ |
| *Periconia muchuanensis* | UESTCC 23.02605 (T) | China: Sichuan | Dead branches of bamboo (Poaceae) | ̶ | PQ067700 | PQ067785 | ̶ | ̶ | ̶ | ̶ | ̶ |
| *Periconia neobrittanica* | CBS 1460625 (T) | USA: California | Leaves of Melaleuca styphelioides × lanceolata | ̶ | NG_068342 | NR_166344 | ̶ | ̶ | ̶ | ̶ | ̶ |

**Table S2.** Continued.

| **Species** | **Specimen/culture** | **Locality** | **Host and substrate** | **SSU** | **LSU** | **ITS** | ***ACT*** | ***RPB2*** | ***TEF1*** | ***TUB2*** | **Genome** |
| --- | --- | --- | --- | --- | --- | --- | --- | --- | --- | --- | --- |
| *Periconia neobrittanica* | TJU_JAN40 | China: Tianjin | Air | ̶ | ̶ | OM237152 | ̶ | ̶ | ̶ | ̶ | ̶ |
| *Periconia neomacrospinosa* | A113 | Brazil: São Paulo | Roots of sugarcane (Poaceae) | ̶ | MT683277 | MT683263 | MT757140 | ̶ | MT793766 | MT820145 | ̶ |
| *Periconia neomacrospinosa* | A115 | Brazil: São Paulo | Roots of sugarcane (Poaceae) | ̶ | MT683278 | MT683264 | MT757142 | ̶ | MT793768 | MT820146 | ̶ |
| *Periconia neomacrospinosa* | A126 | Brazil: São Paulo | Roots of sugarcane (Poaceae) | ̶ | MT683285 | MT683274 | MT757151 | ̶ | MT793777 | MT820153 | ̶ |
| *Periconia neomacrospinosa* | DSE2036 | Hungary: Fülöpháza | Roots of Festuca vaginata (Poaceae) | ̶ | Genome | OM337552 | Genome | Genome | Genome | Genome | GCA_003073855.1 |
| *Periconia neomacrospinosa* | NME00180 | China: Inner Mongolia | Roots of Cleistogenes serotina (Poaceae) | ̶ | PP106211 | PP106191 | PP112474 | PP112501 | PP112529 | PP112554 | ̶ |
| *Periconia neomacrospinosa* | GDMCC 3.10425 (T) | China: Inner Mongolia | Roots of Cleistogenes serotina (Poaceae) | ̶ | PP106212 | PP106192 | PP112475 | PP112502 | PP112530 | PP112555 | ̶ |
| *Periconia neomacrospinosa* | TU41 | Mongolian: Nalaikh | Roots of Stipa krylovii (Poaceae) | ̶ | MN515279 | MN537704 | ̶ | ̶ | MN535276 | ̶ | ̶ |
| *Periconia neomacrospinosa* | ZJE01828 | China: Zhejiang | Roots of Miscanthus sinensis (Poaceae) | ̶ | PP106237 | PP106198 | PP112477 | PP112504 | PP112531 | PP112556 | ̶ |
| *Periconia neomacrospinosa* | ZJE01829 | China: Zhejiang | Roots of Miscanthus sinensis (Poaceae) | ̶ | PP106238 | PP106199 | PP112476 | PP112503 | PP112532 | PP112557 | ̶ |
| *Periconia neominutissima* | CBS 1495145 (T) | France: Normandy | Leaves of Poaceae sp. | ̶ | NG_242103 | NR_189522 | ̶ | ̶ | ̶ | ̶ | ̶ |
| *Periconia neominutissima* | 10b | ̶ | Arthropod cadaver | ̶ | ̶ | KX394553 | ̶ | ̶ | ̶ | ̶ | ̶ |
| *Periconia palmicola* | MFLUCC 14-04005 (T) | Thailand: Chiang Rai | Dead fallen leaves of Trachycarpus fortunei (Arecaceae) | MN648319 | NG_068917 | ̶ | ̶ | ̶ | MN821070 | ̶ | ̶ |

**Table S2.** Continued.

| **Species** | **Specimen/culture** | **Locality** | **Host and substrate** | **SSU** | **LSU** | **ITS** | ***ACT*** | ***RPB2*** | ***TEF1*** | ***TUB2*** | **Genome** |
| --- | --- | --- | --- | --- | --- | --- | --- | --- | --- | --- | --- |
| *Periconia penniseti* | CGMCC 3.239285 (T) | China: Sichuan | Dead culms of Pennisetum sp. (Poaceae) | NG_242915 | NG_229076 | NR_185799 | ̶ | OP961462 | OP961437 | ̶ | ̶ |
| *Periconia philadelphiana* | CBS 1496815 (T) | USA: Philadelphia | HVAC coil swab | ̶ | NG_243245 | NR_190971 | ̶ | ̶ | ̶ | ̶ | ̶ |
| *Periconia prolifica* | CBS 209.645 (T) | USA: California | - | ̶ | MH870050 | NR_160097 | ̶ | ̶ | ̶ | ̶ | ̶ |
| *Periconia prolifica* (as Periconiella saccharicola) | CBS 900.69 | Brazil | - | ̶ | ̶ | MH859476 | ̶ | ̶ | ̶ | ̶ | ̶ |
| *Periconia pseudobyssoides* | DUCC 0850 | China: Yunnan | Submerged wood | ̶ | MG333494 | MG333491 | ̶ | ̶ | MG438280 | ̶ | ̶ |
| *Periconia pseudobyssoides* | MAFF 243868 | Japan: Aomori | *Rodgersia podophylla* (Saxifragaceae) | AB797278 | AB807568 | LC014587 | ̶ | ̶ | AB808544 | ̶ | ̶ |
| *Periconia pseudobyssoides* | MAFF 243874 | Japan: Iwate | *Berchemia floribunda* (Rhamnaceae) | AB797270 | AB807560 | LC014588 | ̶ | ̶ | AB808536 | ̶ | ̶ |
| *Periconia pseudobyssoides* | SICAUCC 23-0045 | China: Sichuan | Dead petiole of Juglans regia | PP003822 | PP057954 | PP060661 | ̶ | ̶ | PP061140 | ̶ | ̶ |
| *Periconia pseudodigitata* | CBS 1396995 (T) | Japan: Hokkaido | *Phragmites australis* (Poaceae) | NG_064850 | NG_059396 | NR_153490 | ̶ | ̶ | AB808540 | ̶ | ̶ |
| *Periconia pseudodigitata* | JCM 13164 | Japan: Aomori | *Phragmites australis* (Poaceae) | AB797272 | AB807562 | LC014589 | ̶ | ̶ | AB808538 | ̶ | ̶ |
| *Periconia pseudodigitata* | TJU_NOV4 | China: Tianjin | Air | ̶ | ̶ | OM237062 | ̶ | ̶ | ̶ | ̶ | ̶ |
| *Periconia sahariana* | CBS 320.79 | Algeria | - | ̶ | MH872978 | MH861211 | ̶ | ̶ | ̶ | ̶ | ̶ |
| *Periconia salina* | MFLU 19-12355 (T) | UK: Carmarthenshire | Unidentified bark | MN017912 | MN017846 | MN047086 | ̶ | ̶ | ̶ | ̶ | ̶ |

**Table S2.** Continued.

| **Species** | **Specimen/culture** | **Locality** | **Host and substrate** | **SSU** | **LSU** | **ITS** | ***ACT*** | ***RPB2*** | ***TEF1*** | ***TUB2*** | **Genome** |
| --- | --- | --- | --- | --- | --- | --- | --- | --- | --- | --- | --- |
| *Periconia shannanensis* | KUNCC24-177845 (T) | China: Sichuan | Dead Artemisia | PP968558 | PP968555 | PP968552 | ̶ | ̶ | PQ226770 | ̶ | ̶ |
| *Periconia shannanensis* | KUNCC24-17786 | China: Tibet | Dead Artemisia | PP968559 | PP968556 | PP968553 | ̶ | ̶ | PQ226771 | ̶ | ̶ |
| *Periconia sichuanensis* | CGMCC 3.255985 (T) | China: Sichuan | Dead branches of bamboo (Poaceae) | PQ066545 | PQ067697 | PQ067867 | ̶ | ̶ | PQ278551 | ̶ | ̶ |
| *Periconia sichuanensis* | UESTCC 23.02595 (T) | China: Sichuan | Dead branches of bamboo (Poaceae) | ̶ | PQ067699 | PQ067869 | ̶ | ̶ | PQ278552 | ̶ | ̶ |
| *Periconia sp.* | YNE01205 | China: Yunnan | Roots of Poaceae sp. | ̶ | PP106230 | PP106208 | PP112493 | PP112520 | PP112551 | PP112576 | ̶ |
| *Periconia sp.* | YNE01210 | China: Yunnan | Roots of Poaceae sp. | ̶ | PP106232 | PP106210 | PP112495 | PP112522 | PP112553 | PP112577 | ̶ |
| *Periconia sp.* | YNE01211 | China: Yunnan | Roots of Poaceae sp. | ̶ | PP106233 | PP106209 | PP112494 | PP112521 | PP112552 | PP112578 | ̶ |
| *Periconia* sp. | R9002 | China: Dalian | Feces of Homo sapiens | Genome | Genome | Genome | Genome | Genome | Genome | Genome | GCA_023627715.1 |
| *Periconia spodiopogonis* | CGMCC 3.239325 (T) | China: Sichuan | Dead culms of Spodiopogon ludingensis (Poaceae) | NG_242914 | NG_229075 | NR_185798 | ̶ | OP961454 | OP961429 | ̶ | ̶ |
| *Periconia spodiopogonis* | HKAS 135635 | China: Tibet | Bamboo | PQ675370 | ̶ | PP952765 | ̶ | ̶ | ̶ | ̶ | ̶ |
| *Periconia submersa* | MFLUCC 16-10985 (T) | China: Yunnan | Submerged wood | ̶ | KY794706 | KY794702 | ̶ | ̶ | KY814761 | ̶ | ̶ |
| *Periconia thailandica* | MFLUCC 17-00655 (T) | Thailand: Kamphaeng | Decaying bamboo (Poaceae) | KY753889 | KY753888 | NR_189802 | ̶ | ̶ | ̶ | ̶ | ̶ |
| *Periconia thailandica* | SICAUCC 23-0046 | China: Sichuan | Dead twigs of Juglans regia | PP003823 | PP057955 | PP060662 | ̶ | ̶ | PP061141 | ̶ | ̶ |
| *Periconia thysanolaenae* | KUMCC 20-02625 (T) | China: Yunnan | Dead culms of Thysanolaena latifolia (Poaceae) | NG_081407 | NG_081511 | NR_176751 | ̶ | ̶ | MW460896 | ̶ | ̶ |
| *Periconia thysanolaenae* | KUNCC 23-13119 | China: Yunnan | Unknown submerged decaying wood | ̶ | PV536359 | PQ845910 | ̶ | ̶ | ̶ | ̶ | ̶ |

**Table S2.** Continued.

| **Species** | **Specimen/culture** | **Locality** | **Host and substrate** | **SSU** | **LSU** | **ITS** | ***ACT*** | ***RPB2*** | ***TEF1*** | ***TUB2*** | **Genome** |
| --- | --- | --- | --- | --- | --- | --- | --- | --- | --- | --- | --- |
| *Periconia variicolor* | CBS 1203745 (T) | USA: Puerto Rico | Water of saltern | ̶ | ̶ | DQ336713 | ̶ | ̶ | ̶ | ̶ | ̶ |
| *Periconia variicolor* | S217 | China: Guizhou | *Metagentiana rhodantha* (Gentianaceae) | ̶ | ̶ | MT576434 | ̶ | ̶ | ̶ | ̶ | ̶ |
| *Periconia verrucosa* | MFLUCC 17-21585 (T) | Belgium: Flemish Brabant | Dead stems of Clematis viticella (Ranunculaceae) | NG_070659 | MT214572 | NR_171873 | ̶ | ̶ | MT394631 | ̶ | ̶ |
| *Periconia verrucosa* | UESTCC 22.0136 | China: Sichuan | Dead leaves of Stipa tenuissima (Poaceae) | OP956035 | OP955991 | OP955966 | ̶ | OP961457 | OP961432 | ̶ | ̶ |
| *Periconia verrucosa* | UESTCC 22.0149 | China: Sichuan | Dead culms of Phyllostachys nigra (Poaceae) | OP956045 | OP956001 | OP955976 | ̶ | OP961464 | OP961442 | ̶ | ̶ |
| *Periconia wurfbainiae* | ZHKUCC 23-09995 (T) | China: Guangdong | Dead stems of Wurfbainia villosa (Zingiberaceae) | NG_243019 | NG_243971 | OR995586 | ̶ | ̶ | PP025972 | ̶ | ̶ |
| *Periconia wurfbainiae* | ZHKUCC 23-1000 | China: Guangdong | Dead stems of Wurfbainia villosa (Zingiberaceae) | PP277727 | OR995593 | OR995587 | ̶ | ̶ | PP025973 | ̶ | ̶ |
| *Periconia yangjiangensis* | ZHKUCC 23-09975 (T) | China: Guangdong | Dead stems of Wurfbainia villosa (Zingiberaceae) | NG_243018 | NG_243970 | OR995584 | ̶ | ̶ | PP025970 | ̶ | ̶ |
| *Periconia yangjiangensis* | ZHKUCC 23-0998 | China: Guangdong | Dead stems of Wurfbainia villosa (Zingiberaceae) | PP277725 | OR995591 | OR995585 | ̶ | ̶ | PP025971 | ̶ | ̶ |
| *Periconia yantingensis* | SICAUCC 23-00475 (T) | China: Sichuan | Dead twigs of Juglans regia | PP003824 | PP057956 | PP060663 | ̶ | ̶ | PP061142 | ̶ | ̶ |

**Table S2.** Continued.

| **Species** | **Specimen/culture** | **Locality** | **Host and substrate** | **SSU** | **LSU** | **ITS** | ***ACT*** | ***RPB2*** | ***TEF1*** | ***TUB2*** | **Genome** |
| --- | --- | --- | --- | --- | --- | --- | --- | --- | --- | --- | --- |
| *Periconia yantingensis* | SICAUCC 23-0147 | China: Sichuan | Dead twigs of Juglans regia | PP853378 | PP826165 | PP844875 | ̶ | ̶ | PP850053 | ̶ | ̶ |
| *Periconia yunnanensis* | YNE01674 | China: Yunnan | Leaves of Oryza meyeriana subsp. granulata (Poaceae) | ̶ | PP106235 | PP106206 | PP112488 | PP112516 | PP112544 | PP112569 | ̶ |
| *Periconia yunnanensis* | YNE01688 | China: Yunnan | Leaves of Oryza meyeriana subsp. granulata (Poaceae) | ̶ | PP106236 | PP106207 | PP112489 | PP112517 | PP112545 | PP112570 | ̶ |
| *Periconia yunnanensis* | KUNCC 23-142595 (T) | China: Yunnan | Submerged decaying wood | ̶ | PP189906 | PQ340468 | ̶ | ̶ | PQ456960 | ̶ | ̶ |
| *Periconia yunnanensis* | KUNCC 23-16910 | China: Yunnan | Submerged decaying wood | ̶ | PP189925 | PQ607755 | ̶ | ̶ | PQ456959 | ̶ | ̶ |

Notes: (1) Newly generated sequences are indicated in red. (2) For genomic loci, accession numbers are deposited in National Center for Biotechnology Information (NCBI) GenBank. (3) For whole genome, accession numbers beginning with “GCA_XXXXXXXXX.X” are deposited in NCBI GenBank.

**Supplementary Figures**


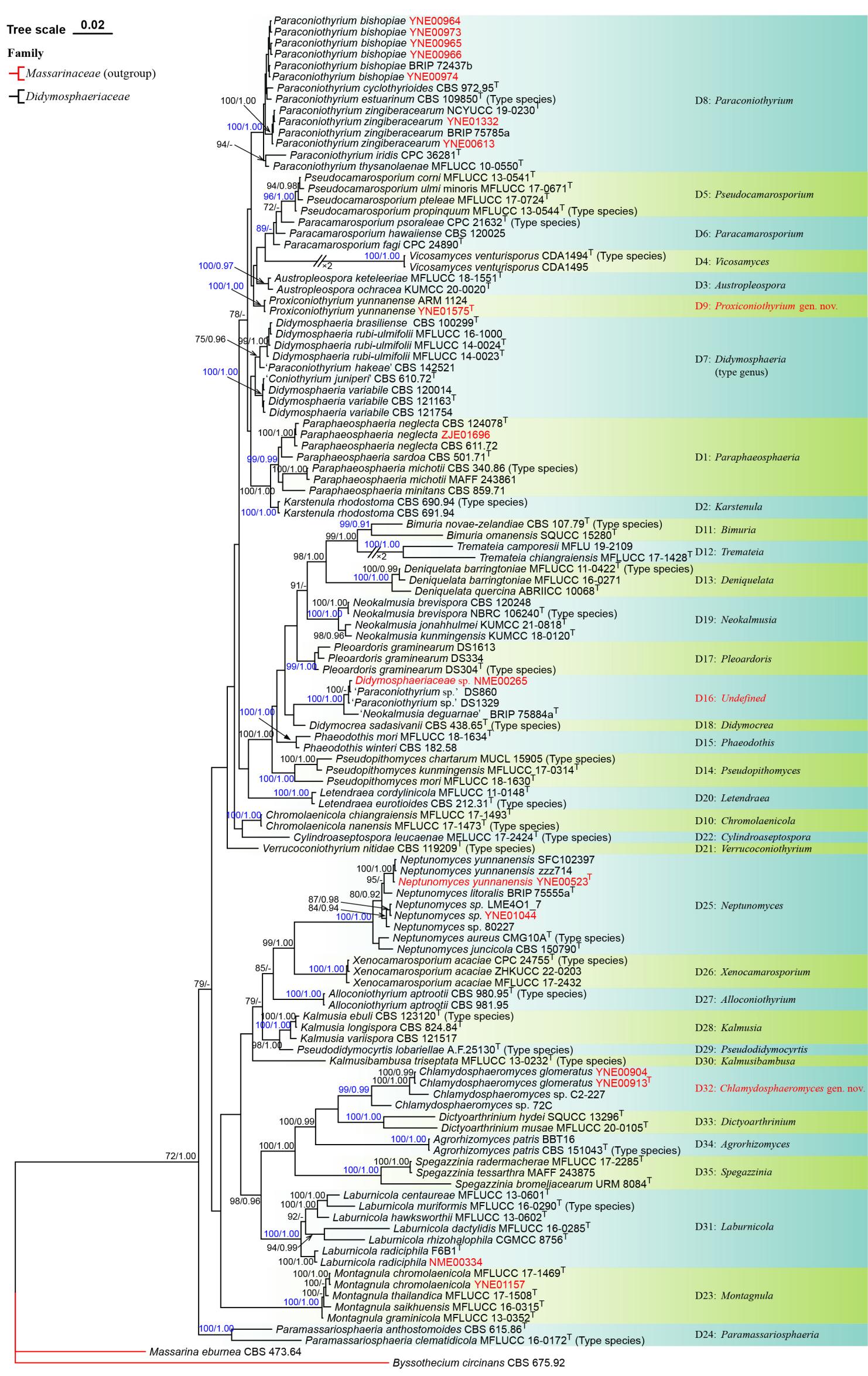


**Figure S1.** Phylogenetic tree of *Didymosphaeriaceae* inferred from rDNA. MLBP ≥ 70% and BIPP ≥ 0.90 are presented above the branch leading to that node (MLBP/BIBP). Strains in this study are indicated in red and the support values for each distinct major clade are displayed in blue. Holotype and ex-type strains are denoted by superscript T, and type species of the corresponding genus are marked in parentheses.


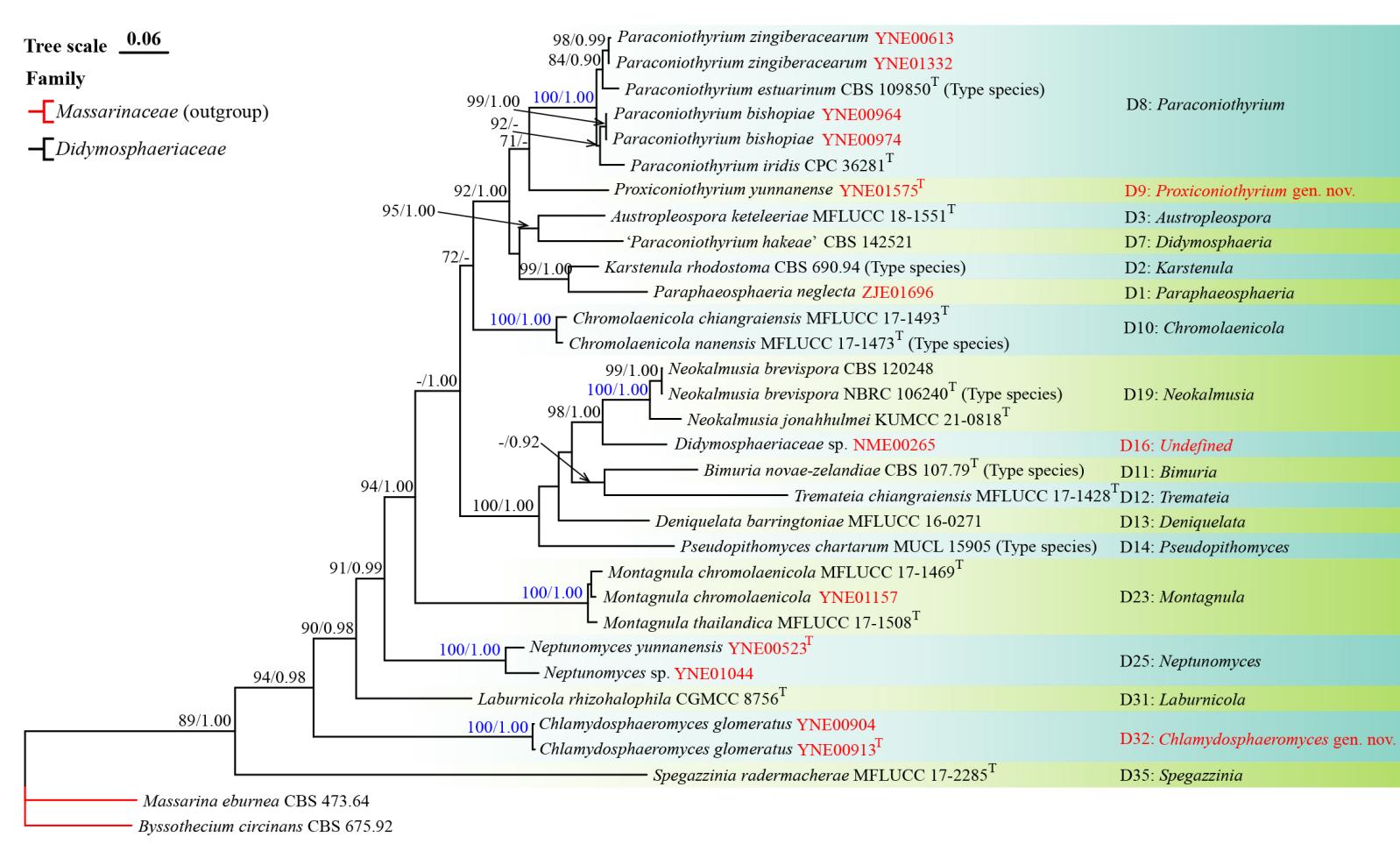


**Figure S2.** Phylogenetic tree of *Didymosphaeriaceae* inferred from *RPB2* sequence dataset. MLBP ≥ 70 % and BIPP ≥ 0.90 are presented above the branch leading to that node (MLBP/BIBP). Strains in this study are indicated in red and the support values for each distinct major clade are displayed in blue. Holotype and ex-type strains are denoted by superscript T, and type species of the corresponding genus are marked in parentheses.


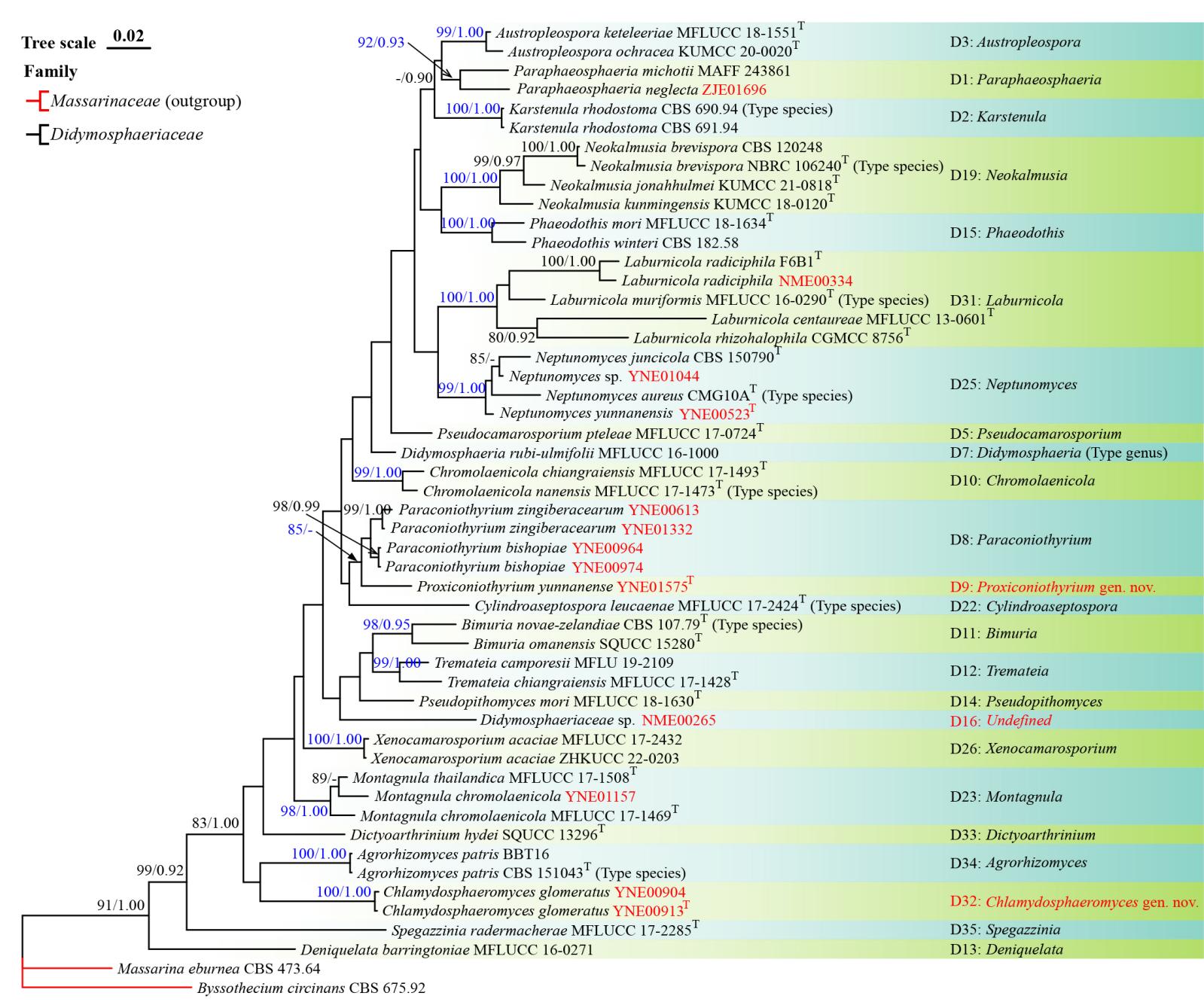


**Figure S3.** Phylogenetic tree of *Didymosphaeriaceae* inferred from *TEF1* sequence dataset. MLBP ≥ 70% and BIPP ≥ 0.90 are presented above the branch leading to that node (MLBP/BIBP). Strains in this study are indicated in red and the support values for each distinct major clade are displayed in blue. Holotype and ex-type strains are denoted by superscript T, and type species of the corresponding genus are marked in parentheses.


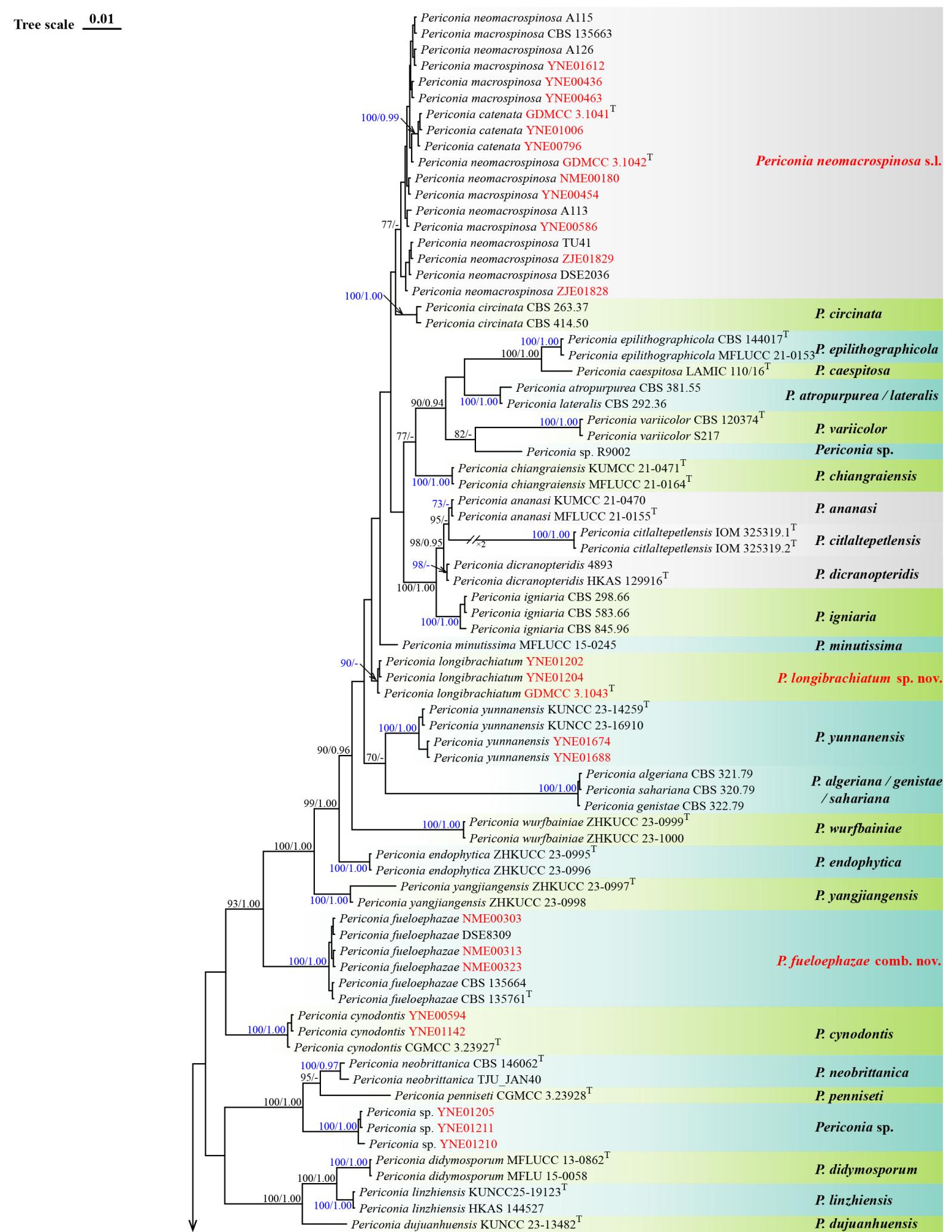


**Figure S4.** Phylogenetic tree of *Periconiaceae* inferred from rDNA. MLBP ≥ 70% and BIPP ≥ 0.90 are presented above the branch leading to that node (MLBP/BIBP). Strains in this study are indicated in red and the support values for each distinct major clade are displayed in blue. Holotype and ex-type strains are denoted by superscript T.


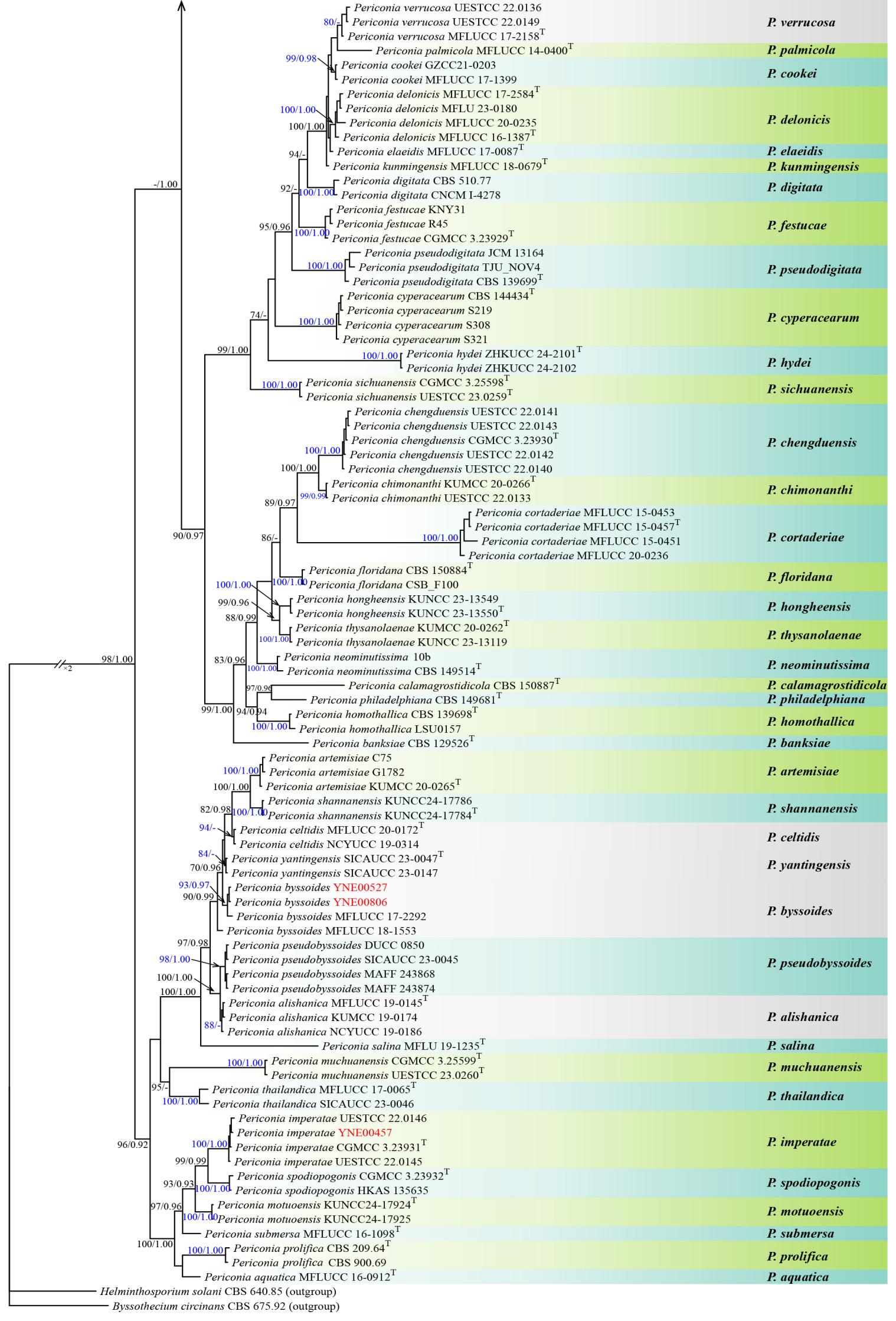


**Figure S4.** (Continued).


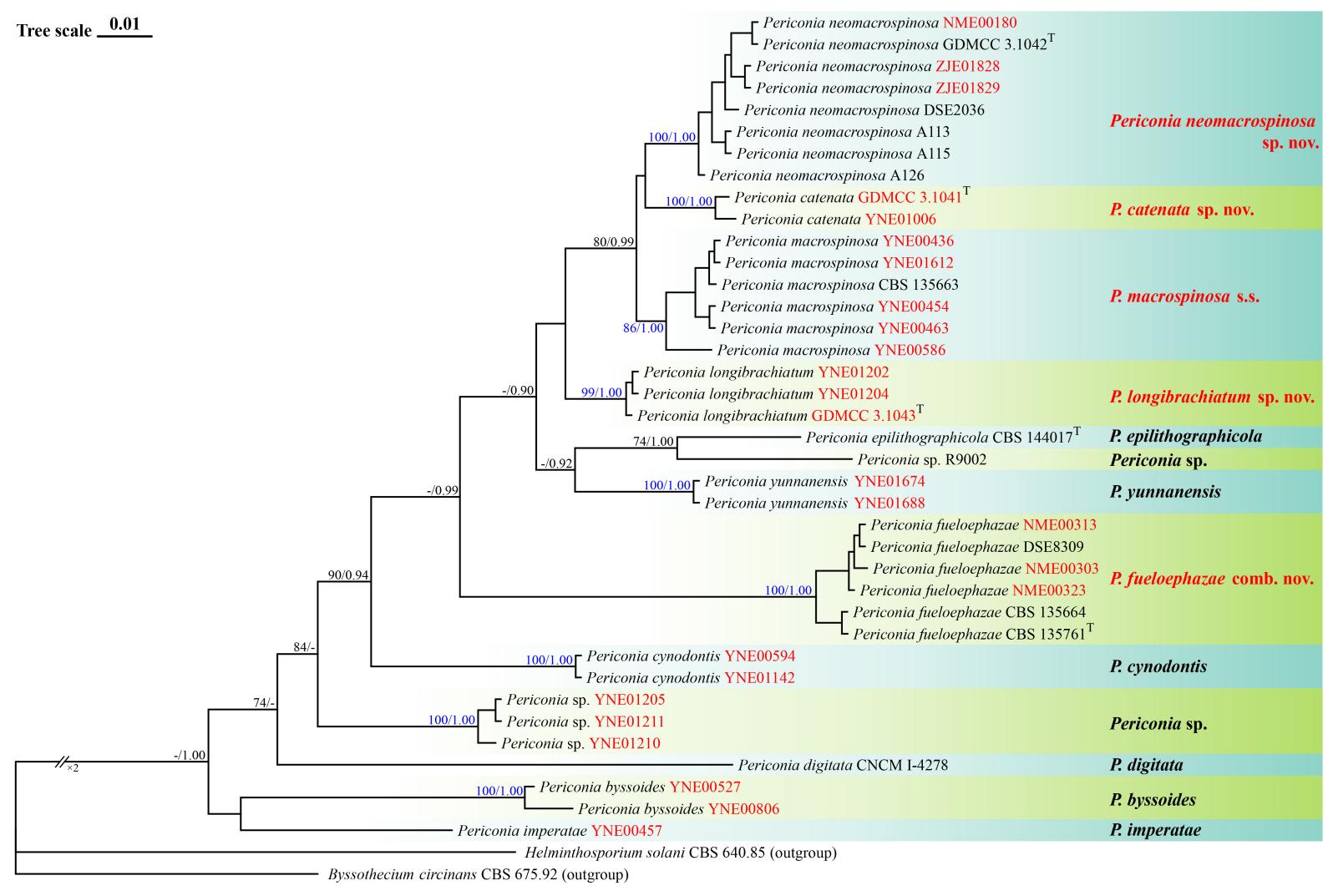


**Figure S5.** Phylogenetic tree of *Periconiaceae* inferred from *ACT* sequence dataset. MLBP ≥ 70% and BIPP ≥ 0.90 are presented above the branch leading to that node (MLBP/BIBP). Strains in this study are indicated in red and the support values for each distinct major clade are displayed in blue. Holotype and ex-type strains are denoted by superscript T.


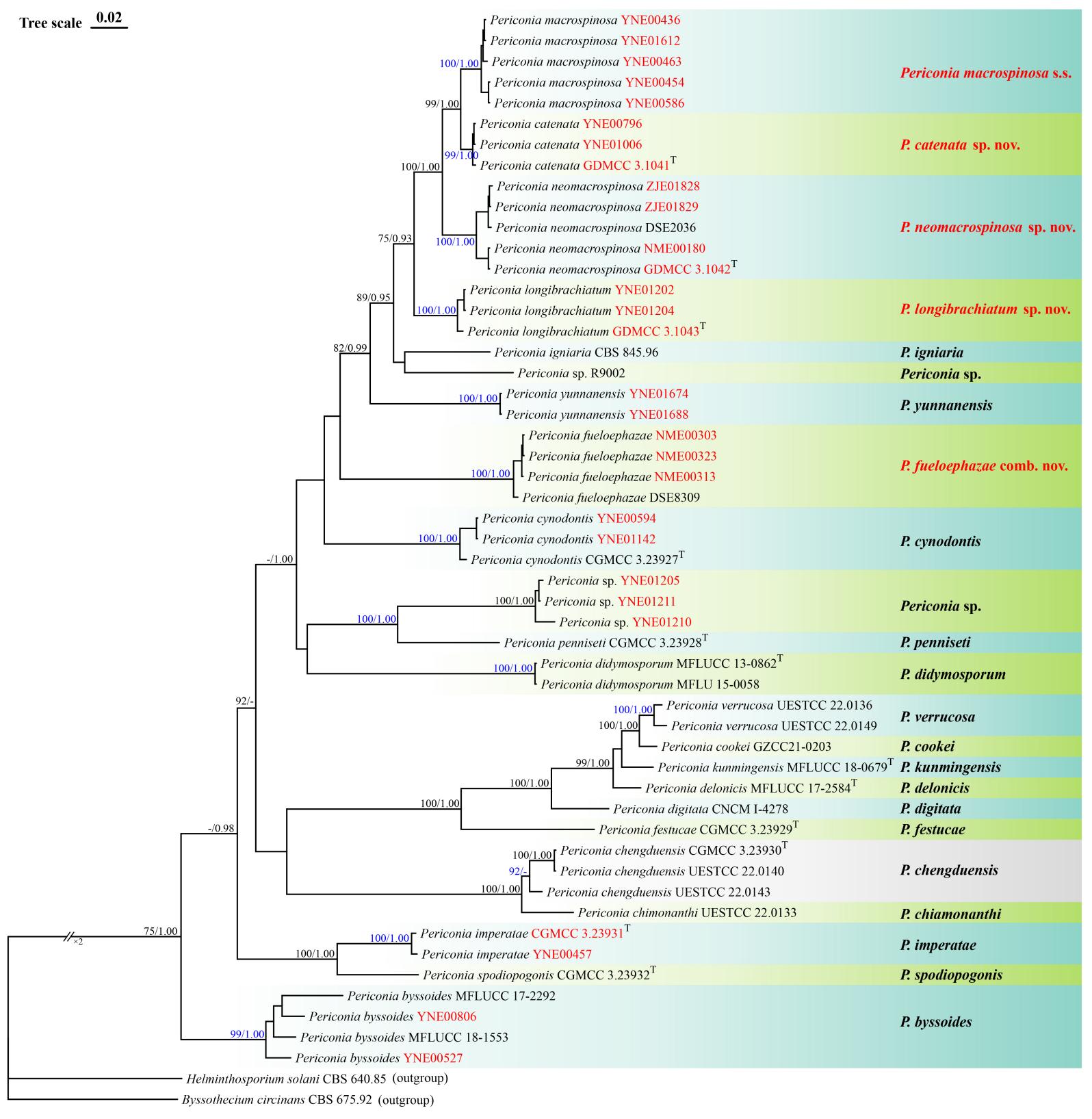


**Figure S6.** Phylogenetic tree of *Periconiaceae* inferred from *RPB2* sequence dataset. MLBP ≥ 70% and BIPP ≥ 0.90 are presented above the branch leading to that node (MLBP/BIBP). Strains in this study are indicated in red and the support values for each distinct major clade are displayed in blue. Holotype and ex-type strains are denoted by superscript T.


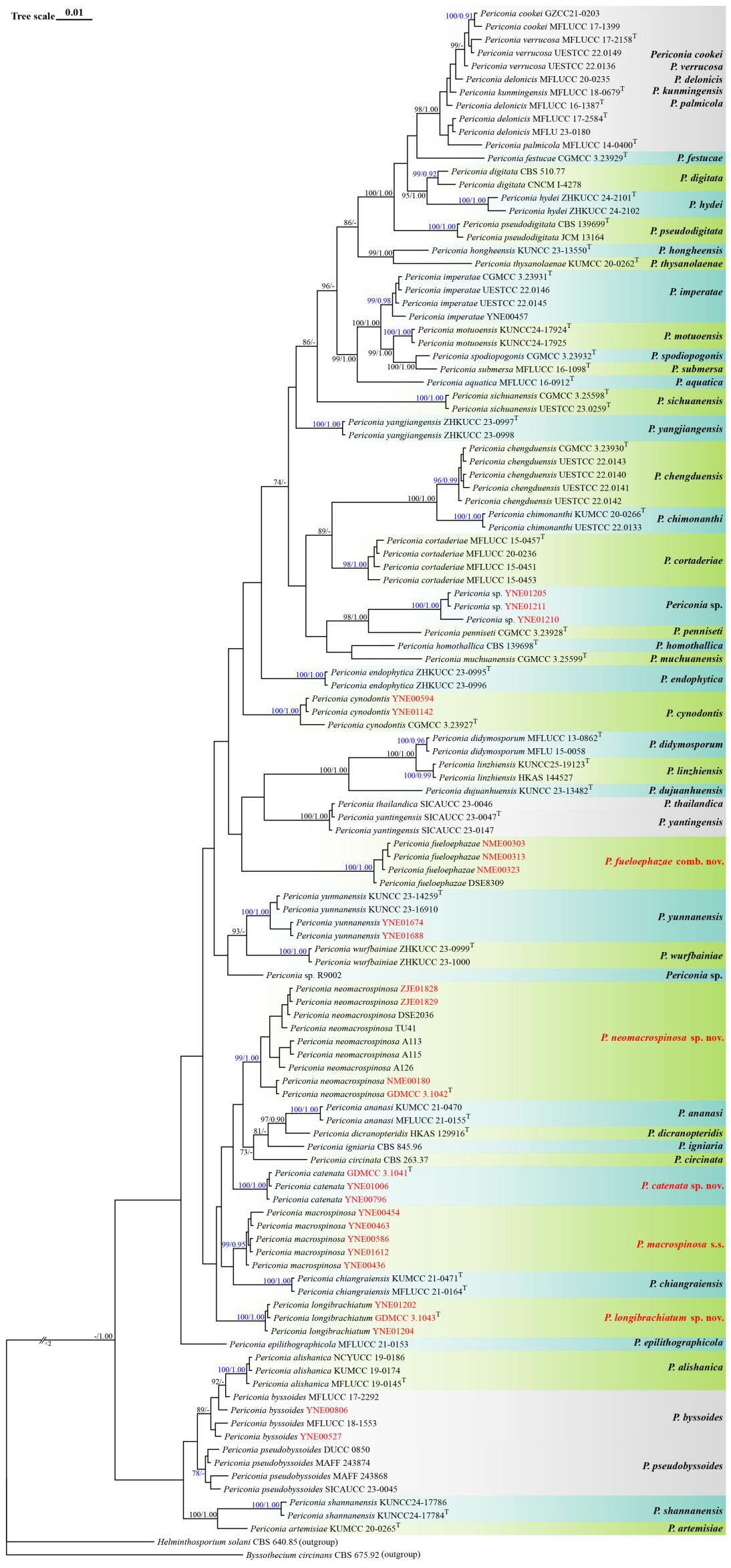


**Figure S7.** Phylogenetic tree of *Periconiaceae* inferred from *TEF1* sequence dataset. MLBP ≥ 70% and BIPP ≥ 0.90 are presented above the branch leading to that node (MLBP/BIBP). Strains in this study are indicated in red and the support values for each distinct major clade are displayed in blue. Holotype and ex-type strains are denoted by superscript T.


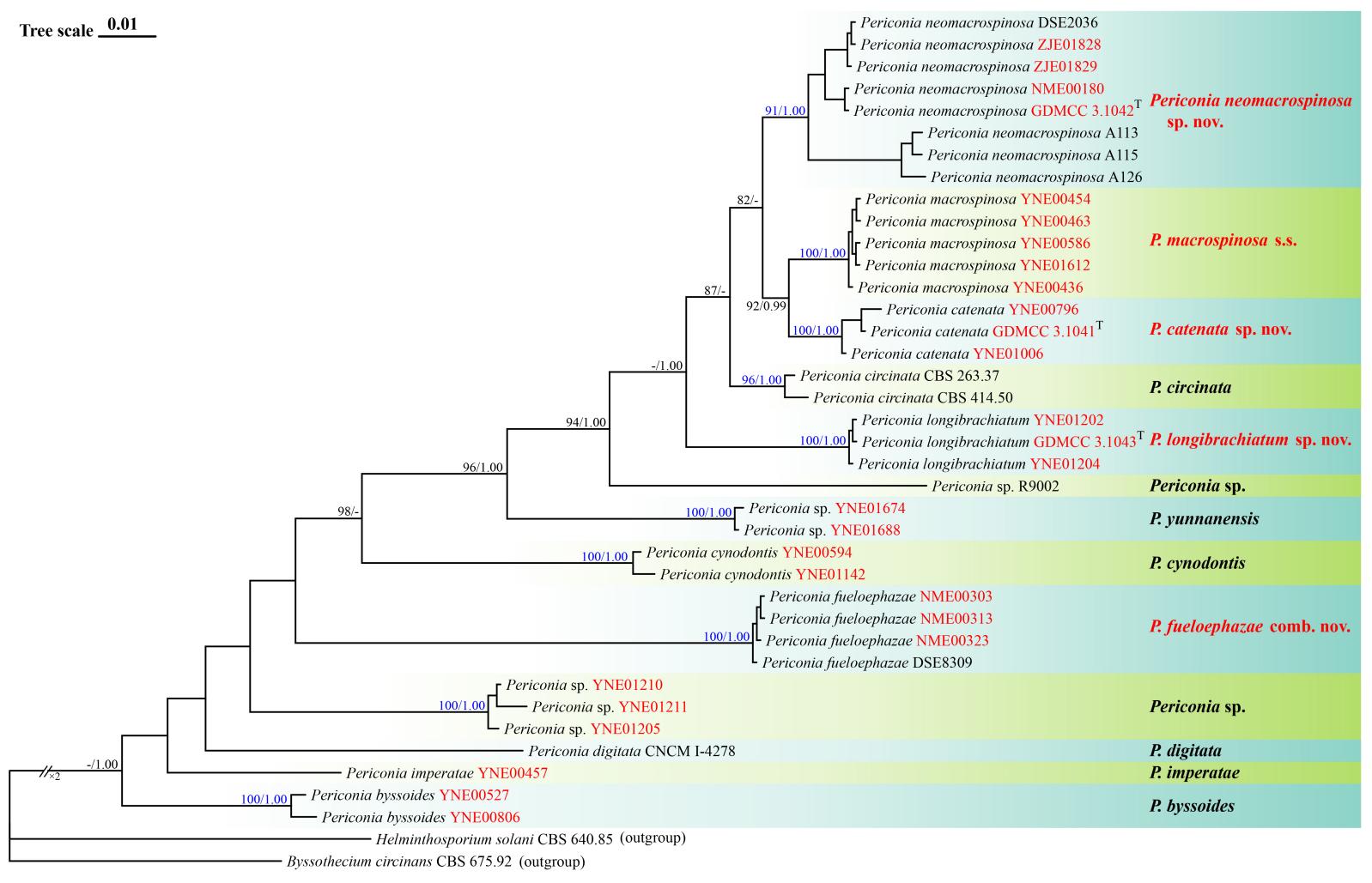


**Figure S8.** Phylogenetic tree of *Periconiaceae* inferred from *TUB2* sequence dataset. MLBP ≥ 70% and BIPP ≥ 0.90 are presented above the branch leading to that node (MLBP/BIBP). Strains in this study are indicated in red and the support values for each distinct major clade are displayed in blue. Holotype and ex-type strains are denoted by superscript T.


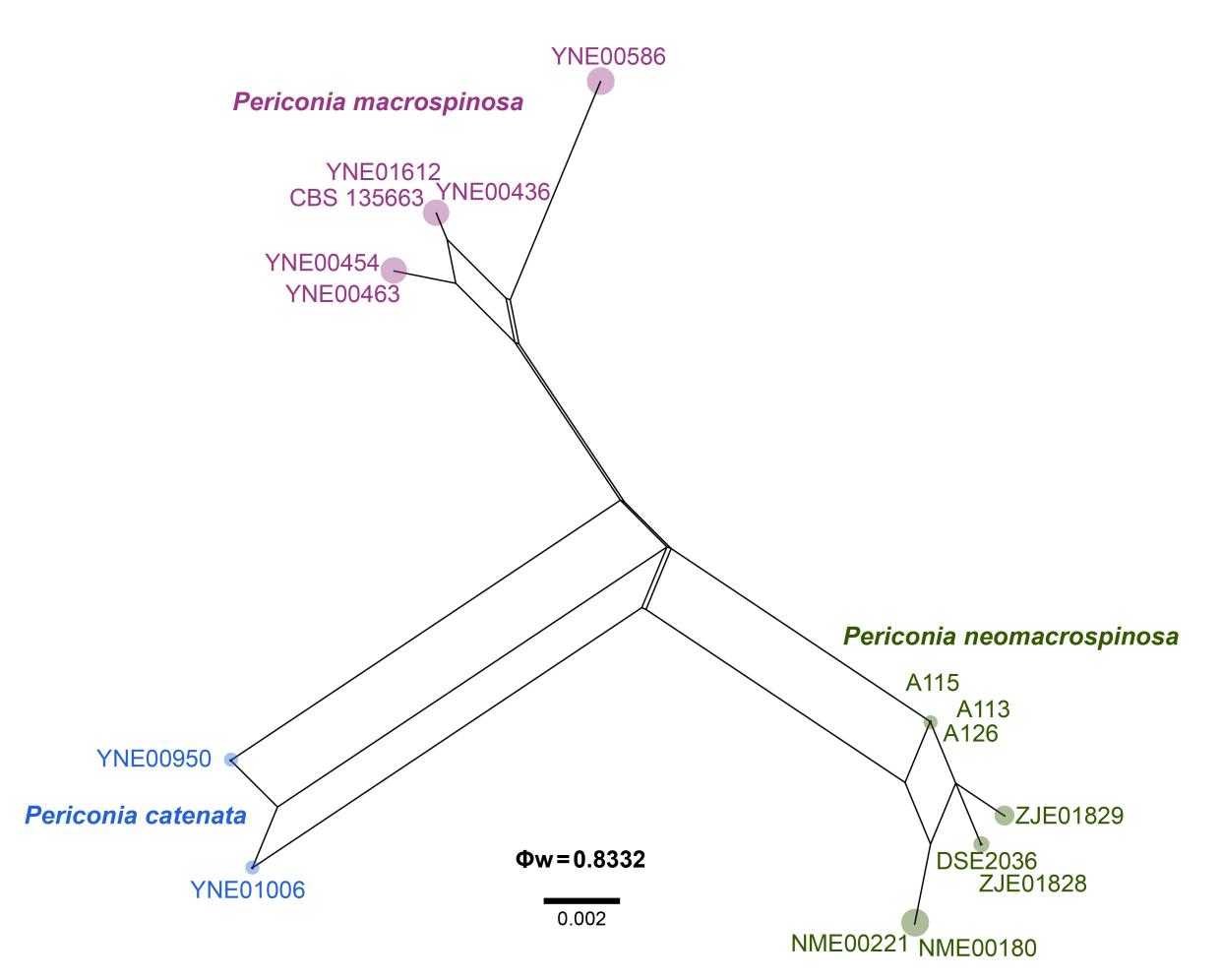


**Figure S9.** Split network of PHI test from the *ACT* sequences for *Periconia macrospinosa* s.l., based on LogDet transformation and NeighborNet algorithm. A PHI index test result (Φw > 0.05) indicates no statistically significant recombination within the dataset.


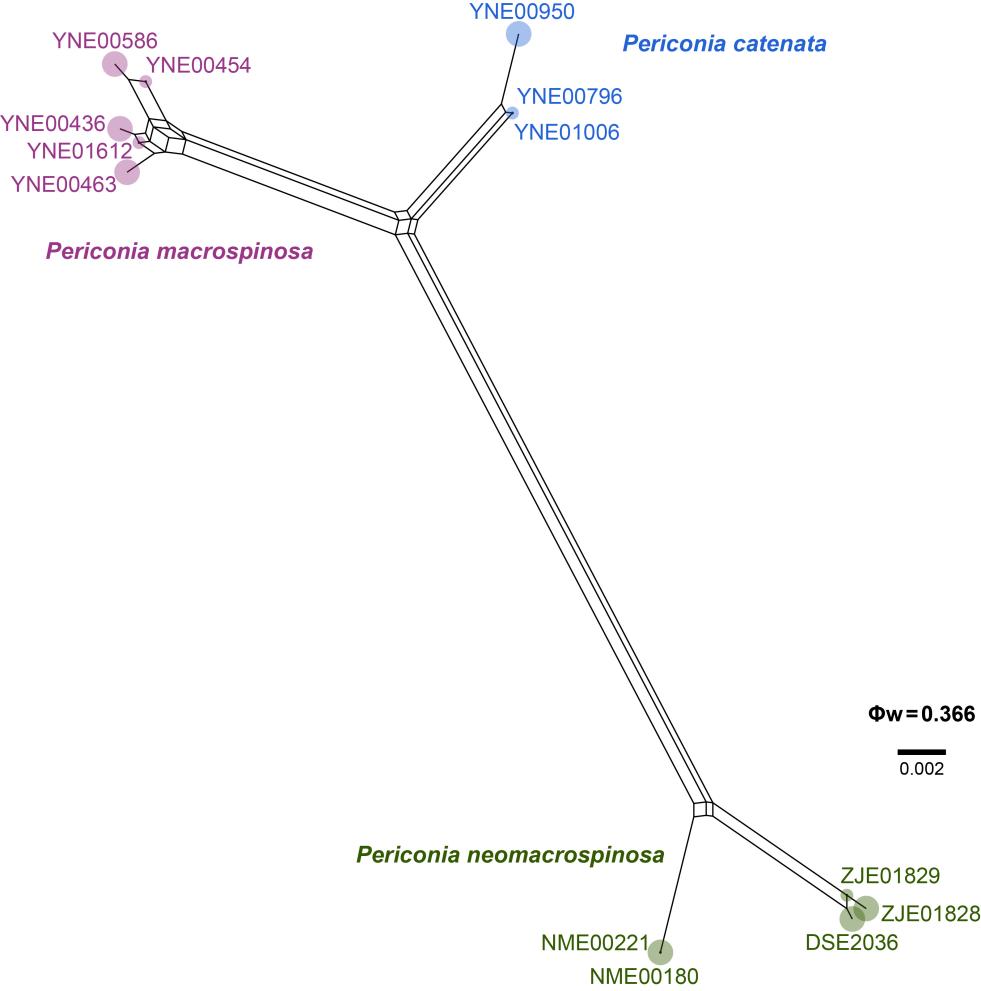


**Figure S10.** Split network of PHI test from the *RPB2* sequences for *Periconia macrospinosa* s.l., based on LogDet transformation and NeighborNet algorithm. A PHI index test result (Φw > 0.05) indicates no statistically significant recombination within the dataset.


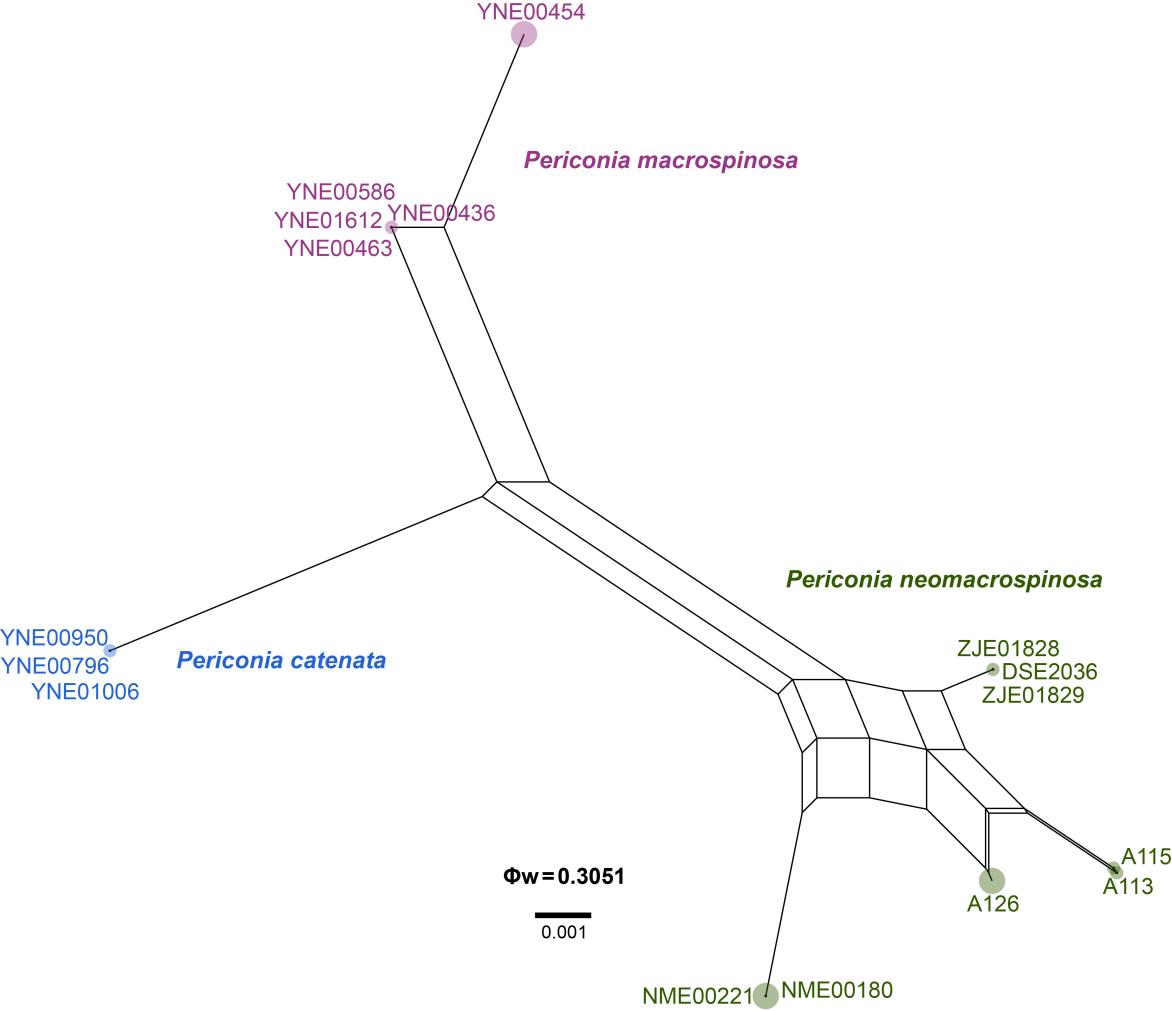


**Figure S11.** Split network of PHI test from the *TEF1* sequences for *Periconia* *macrospinosa* s.l., based on LogDet transformation and NeighborNet algorithm. A PHI index test result (Φw > 0.05) indicates no statistically significant recombination within the dataset.


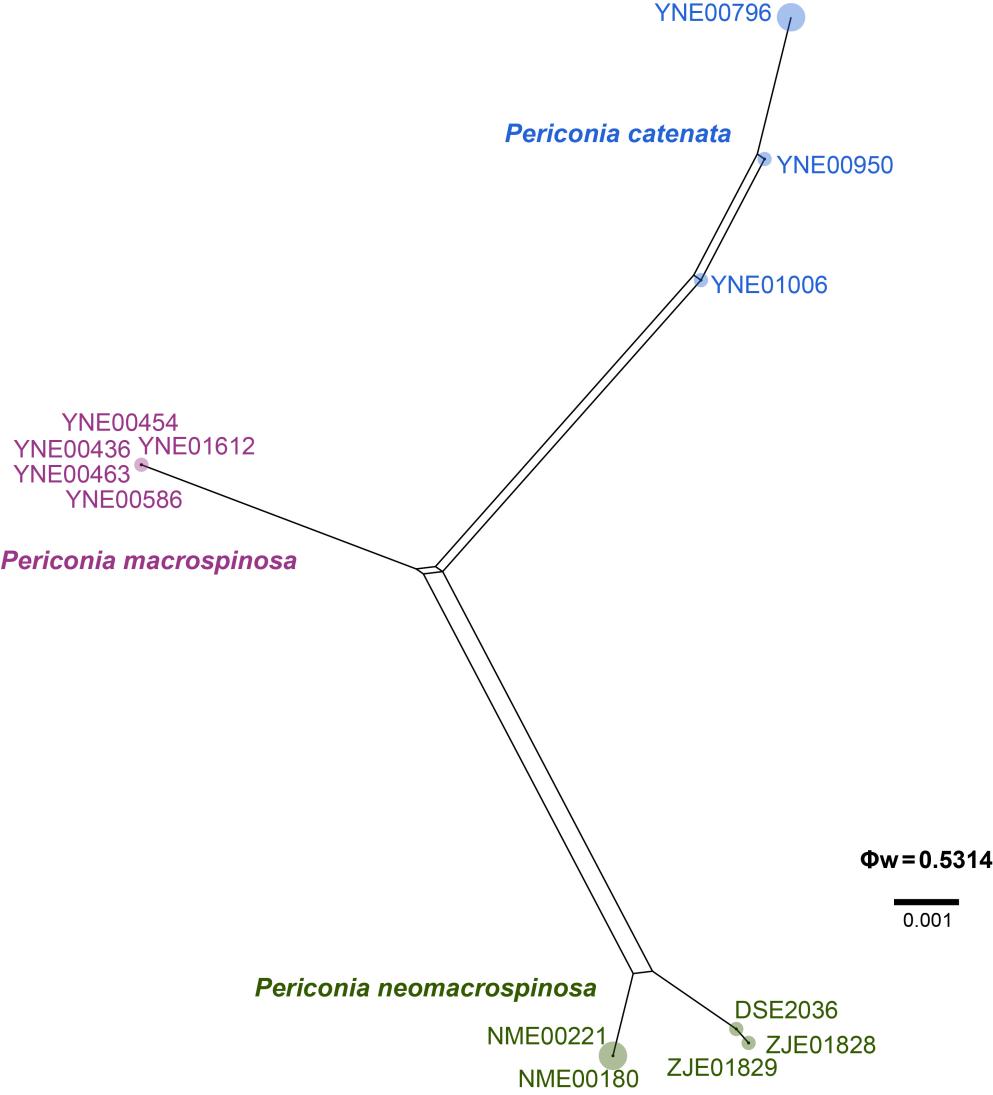


**Figure S12.** Split network of PHI test from the *TUB2* sequences for *Periconia* *macrospinosa* s.l., based on LogDet transformation and NeighborNet algorithm. A PHI index test result (Φw > 0.05) indicates no statistically significant recombination within the dataset.
